# Comparative Genomics of the Sigatoka Disease Complex on Banana Suggests a Link between Parallel Evolutionary Changes in *Pseudocercospora fijiensis* and *Pseudocercospora eumusae* and Increased Virulence on the Banana Host

**DOI:** 10.1371/journal.pgen.1005904

**Published:** 2016-08-11

**Authors:** Ti-Cheng Chang, Anthony Salvucci, Pedro W. Crous, Ioannis Stergiopoulos

**Affiliations:** 1 Department of Plant Pathology, University of California Davis, Davis, California, United States of America; 2 CBS-KNAW Fungal Biodiversity Centre, Utrecht, The Netherlands; CSIRO, AUSTRALIA

## Abstract

The Sigatoka disease complex, caused by the closely-related Dothideomycete fungi *Pseudocercospora musae* (yellow sigatoka), *Pseudocercospora eumusae* (eumusae leaf spot), and *Pseudocercospora fijiensis* (black sigatoka), is currently the most devastating disease on banana worldwide. The three species emerged on bananas from a recent common ancestor and show clear differences in virulence, with *P*. *eumusae* and *P*. *fijiensis* considered the most aggressive. In order to understand the genomic modifications associated with shifts in the species virulence spectra after speciation, and to identify their pathogenic core that can be exploited in disease management programs, we have sequenced and analyzed the genomes of *P*. *eumusae* and *P*. *musae* and compared them with the available genome sequence of *P*. *fijiensis*. Comparative analysis of genome architectures revealed significant differences in genome size, mainly due to different rates of LTR retrotransposon proliferation. Still, gene counts remained relatively equal and in the range of other Dothideomycetes. Phylogenetic reconstruction based on a set of 46 conserved single-copy genes strongly supported an earlier evolutionary radiation of *P*. *fijiensis* from *P*. *musae* and *P*. *eumusae*. However, pairwise analyses of gene content indicated that the more virulent *P*. *eumusae* and *P*. *fijiensis* share complementary patterns of expansions and contractions in core gene families related to metabolism and enzymatic degradation of plant cell walls, suggesting that the evolution of virulence in these two pathogens has, to some extent, been facilitated by convergent changes in metabolic pathways associated with nutrient acquisition and assimilation. In spite of their common ancestry and shared host-specificity, the three species retain fairly dissimilar repertoires of effector proteins, suggesting that they likely evolved different strategies for manipulating the host immune system. Finally, 234 gene families, including seven putative effectors, were exclusively present in the three Sigatoka species, and could thus be related to adaptation to the banana host.

## Author Summary

Understanding the evolutionary and genomic changes involved in the emergence of new pathogens and shifts in virulence spectra is vital for deciphering the biological process of disease emergence and for designing new and effective disease control methods. In this study, we employed comparative genomics in order to examine the nature, diversity, and extent of genomic modifications associated with changes in virulence among *Pseudocercospora musae*, *Pseudocercospora eumusae*, and *Pseudocercospora fijiensis*, the main constituents of the Sigatoka disease complex on banana, currently one of the most destructive diseases on banana worldwide. Our comparative genome analyses have highlighted the role of pathoadaptive changes in virulence associated genes, such as those encoding for effectors, in shaping the underlying differences in virulence spectra among the three species, and also revealed that changes in the size of gene families associated with nutrient acquisition and assimilation are more respectful of the species virulence profiles rather than their evolutionary relationships. Thus, we posit that next to species-specific evolutionary adaptations in virulence-associated genes, the increase in virulence of *P*. *eumusae* and *P*. *fijiensis* has been driven by convergent evolution in metabolic pathways that likely facilitate a higher efficiency of nutrient acquisition, uptake, and utilization.

## Introduction

Bananas and plantains (*Musa* spp.*)* are amongst the world's top five staple food crops, as approximately 100 million tons of bananas are produced annually in nearly 120 countries in tropical and subtropical regions [1]. However, bananas are prone to many diseases that can severely reduce production, and thus pose a threat to global food security. The problem is intensified by the very narrow genetic basis of currently cultivated banana varieties, as most are sterile triploid hybrids (AAA, AAB, ABB) between the wild species *Musa acuminata* (A genome) and *Musa balbisiana* (B genome). This includes desert bananas (AAA) of the Cavendish-subgroup, cooking bananas (AAA or ABB), and nearly all plantain landraces (AAB) [2].

Currently, the so-called *“Sigatoka disease complex”* is one of the most destructive diseases in banana worldwide, reducing yields by more than 50% [3, 4]. The socio-economic impact of the disease is much higher in small farming communities in sub-Saharan Africa, Southeast Asia, and Latin America that depend almost exclusively on the banana crop for their survival. Therefore, managing this disease is of urgent importance and is currently under critical public review for humanitarian, biosafety, and environmental reasons [1, 4].

**Fig 1:**
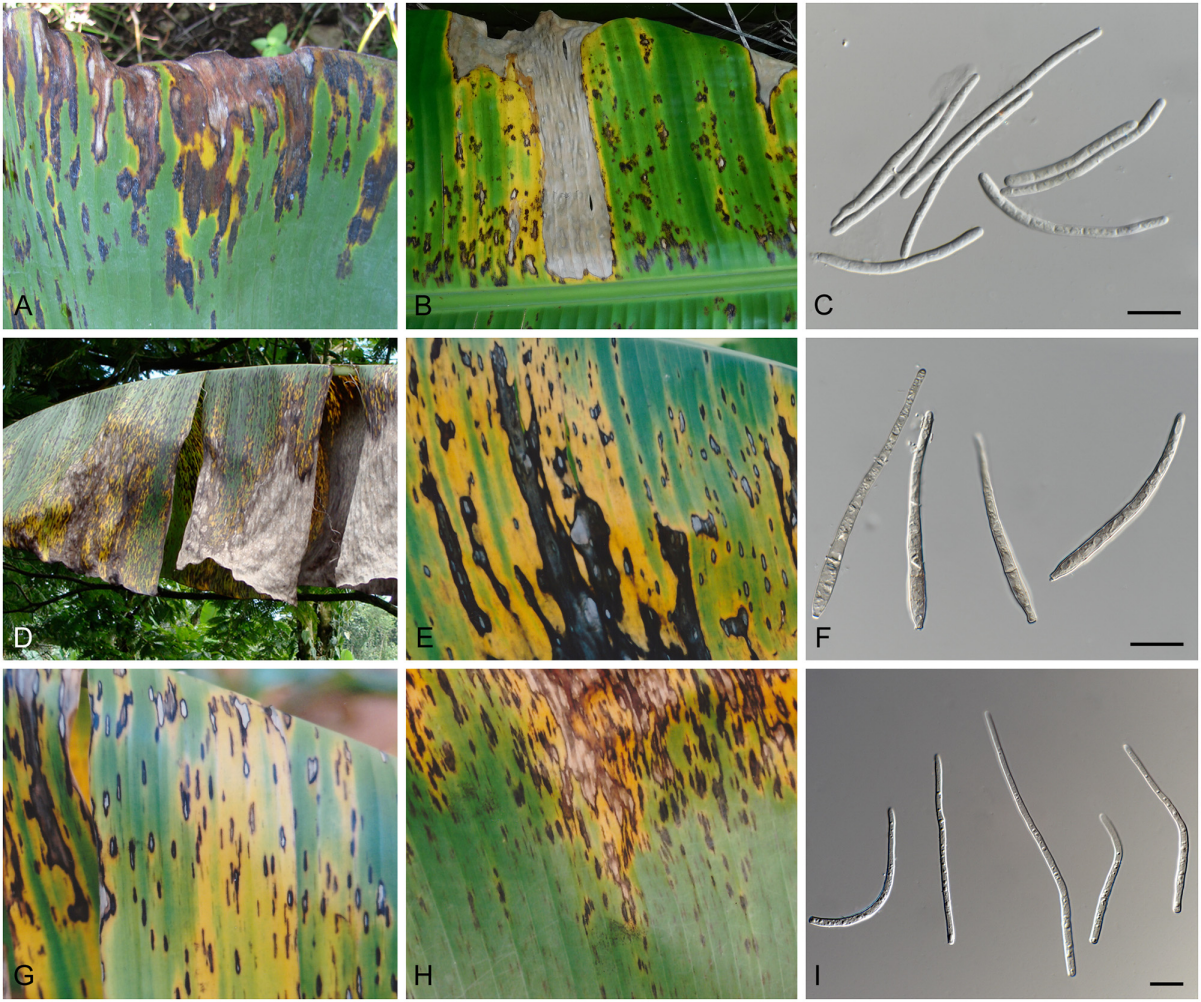
Disease symptoms caused on banana by the three species that constitute the Sigatoka disease complex. (A–C) Leaf symptoms and conidia of *Pseudocercospora eumusae*. (D–F) Leaf symptoms and conidia of *Pseudocercospora fijiensis*. (G–I) Leaf symptoms and conidia of *Pseudocercospora musae*. Scale bars = 10 μm. (leaf photo credits Profs. A. Viljoen and G. Kema).

Three phylogenetically closely related species of *Pseudocercospora* (class Dothideomycetes, order Capnodiales, family Mycosphaerellaceae) have been recognized as the primary constituents of the Sigatoka disease complex in banana, namely *Pseudocercospora fijiensis* (*Pf*) (M. Morelet) Deighton [sexual morph: *Mycosphaerella fijiensis* M. Morelet], causal agent of black Sigatoka or black leaf streak disease, *Pseudocercospora musae* (*Pm*) (Zimm.) Deighton [sexual morph: *Mycosphaerella musicola* R. Leach ex J.L. Mulder], causal agent of yellow Sigatoka disease, and *Pseudocercospora eumusae* (*Pe*) Crous & X. Mourichon (sexual morph: *Mycosphaerella eumusae* Crous & X. Mourichon) causal agent of eumusae leaf spot disease [3–5]. The host range of the three species is believed to be restricted to *Musa* spp., although clear differences in virulence exist amongst them, with *P*. *fijiensis* considered as the most aggressive and *P*. *musae* the least damaging species [5–7] (Fig 1). Despite such differences in virulence, however, the three species share a common hemi-biotrophic lifestyle and disease-cycle on their host, often causing similar and easily confounded symptoms on infected leaves. More specifically, compatible interactions are characterized by a biotrophic latent phase of 3–4 weeks, depending on the specific species/isolate-host interaction, during which the pathogen colonizes the intercellular spaces before any necrotic symptoms appear on the infected leaves. On the other hand, incompatible interactions are expressed either in the form of partial resistance or bear the signatures of a hypersensitivity response (HR), typical of gene-for-gene interactions [3, 5, 8].

In addition to a common phytopathogenic and infectious lifestyle, multilocus DNA analysis has also revealed that the three species form a monophyletic group, and thus are likely to have originated from a common ancestral species [9]. The common evolutionary history of these pathogens was also confirmed by characterization of their mating-type loci, which suggested a stepwise evolution from an heterothallic ancestor splitting first into *P*. *fijiensis* and subsequently into *P*. *musae* and *P*. *eumusae* [10]. Although not exclusively specified, the analysis also suggested that these events are likely to have taken place relatively recently in the evolutionary past of the three pathogens. Indeed, the disease chronology records suggest that all three pathogens emerged in Southeast Asia during the last century, with *P*. *musae* appearing first in the Indonesian island of Java in 1902 from where it rapidly expanded to all banana producing areas of the world, occasionally causing severe epidemics [3, 4]. Nowadays, the pathogen has typically been displaced by the more aggressive *P*. *fijiensis*, which was first recorded in the Sigatoka district of Fiji in 1963, and since then has become the dominant species in areas where the two pathogens co-exist [11]. Compared to *P*. *musae*, *P*. *fijiensis* is able to infect a wider range of cultivars, including ones with resistance to *P*. *musae*, and cause considerably more damage that can affect losses up to 76%, thus endangering food security. At present, *P*. *fijiensis* has spread to most parts of the world where bananas and plantains are grown, and continues to advance to new ecological niches [4, 12, 13]. The third species associated with the Sigatoka disease complex, *P*. *eumusae*, was first described in mid-1990s in Southern and Southeast Asia [6] and, although on the march, so far seems to be restricted to these parts of Asia and some parts of Africa. Notably, *P*. *eumusae* is able to infect banana and plantain cultivars that are resistant to both *P*. *musae* and *P*. *fijiensis*, causing yield losses of up to 40% [3, 4, 6].

Despite the fact that *P*. *musae* was the first pathogen to be described in the disease chronology records, in reality it is possible that the three species co-existed on banana until changes related to the genetics of the pathogens or/and exogenous factors, such as changes in cultural practices and environmental conditions, have prompted the observed alterations in their virulence spectra and the sudden flare-up and over-dominance of one species over the others. A recent study has described more than 20 *Mycosphaerella* species on banana, many of which can co-exist on the same leaf or even the same lesion with the three primary constituents of the Sigatoka disease complex [9]. Although most of these species are only mildly virulent on banana, it is possible that niche sharing by multiple closely related species on the same host could facilitate inter-species exchange of genetic material and result in new species with altered virulence patterns [9].

Understanding the evolutionary and genomic changes involved in the emergence of new pathogens and shifts in virulence spectra is critical. Such knowledge is beyond academic interest alone, as it is vital for deciphering the biological process of disease emergence and for designing new and effective disease control methods. In this study, we employed comparative and evolutionary genomics in order to understand the evolutionary trends and genomic modifications associated with shifts in virulence spectra among *P*. *musae*, *P*. *eumusae*, and *P*. *fijiensis*, the main constituents of the Sigatoka disease complex on banana, and to further identify their pathogenic core that can be exploited in disease management programs. Using next generation sequencing technologies, we have sequenced the genomes of *P*. *eumusae* and *P*. *musae* and compared them with the recently determined 74.1 Mb genome sequence of *P*. *fijiensis* [14]. Genome-wide molecular selection analysis was further used to estimate whether changes in virulence spectra are mainly facilitated by adaptive evolution of the core genome or through species-specific gene acquisitions and losses. Overall, our analysis identified a significant amount of species-specific adaptations, but also revealed convergent patterns of evolution in the two more aggressive pathogens, suggesting that the evolution of virulence traversed through key changes in specific molecular pathways. The results presented in this study enable a deeper understanding of the Sigatoka disease complex and the evolution of virulence in these pathogens and beyond.

## Results and Discussion

### De novo genome assemblies and estimates of genome sizes reveal diversification in genome structures

Whole-genome shotgun sequencing of *P*. *musae* and *P*. *eumusae* on the Illumina Hiseq2500 platform generated a total of 22.6 and 38.9 millions of high quality pair-ended reads (150x150 bp) for each species, respectively, that were first used for an assembly-independent estimation of their genomic characteristics, by *k*-mer analysis (*k* = 17) (Tables 1 and S1, S1 Fig). Based on the total *k*-mer number and the volume peak, genome sizes were estimated to 82.8 Mb for *P*. *musae* and 53.8 Mb for *P*. *eumusae*, thus revealing that, as compared to *P*. *fijiensis* (74.1 Mb) [14], *P*. *musae* has the largest and *P*. *eumusae* the smallest genome size from the three species.

**Table 1:** Summary of the genome assembly and annotation statistics.

	*Pseudocercospora musae*	*Pseudocercospora eumusae*	*Pseudocercospora fijiensis*^a^
Assembly length (Mb, >500 bp)	60.44	47.12	74.14
Assembly length (Mb, >2 Kb)	59.79	45.89	74.14
Scaffolds	3331	2626	56
Scaffolds ≥2 Kb	2879	1562	56
Scaffold L50	353	109	5
Scaffold N50 (Mb)	0.04	0.16	5.9
Estimated genome size (Mb)	82.77	53.79	74.14
Predicted genes	10,632	11,173	13,107
Predicted coding genes	10,548	11,064	13,107
Protein length (median)	367	382	351
GC content of coding DNA (%)	53.27	53.29	53.10

^a^ Data retrieved from Arango Isaza et al. (2016) [14]

**Fig 2:**
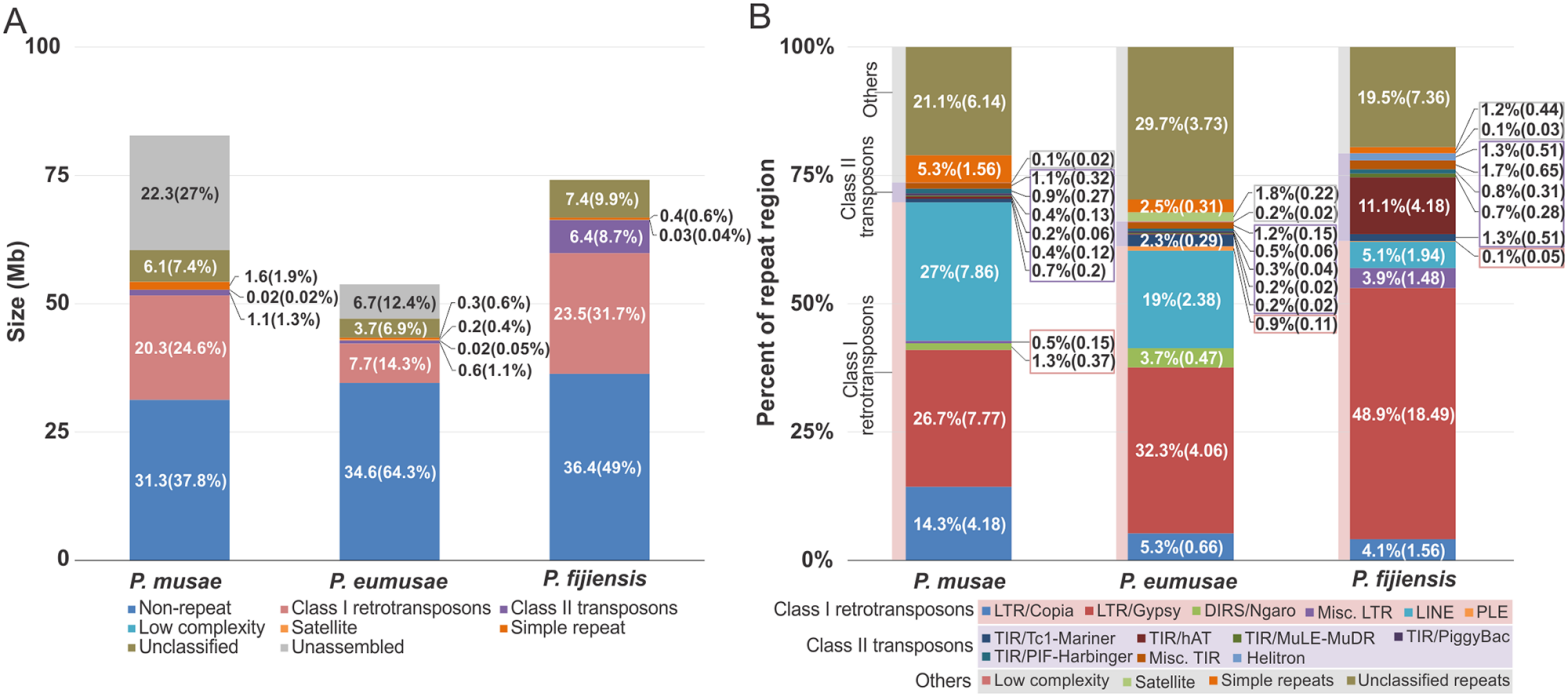
Genome size and composition in *Pseudocercospora musae*, *Pseudocercospora eumusae*, and *Pseudocercospora fijiensis*. (A) Overall genome composition and repeat content in *P*. *musae*, *P*. *eumusae*, and *P*. *fijiensis*. The size (Mb) and proportion (%) of the main components of the species’ genomes are indicated. Given their close evolutionary relationships, the species show considerable differences in genome size mainly due to differences in repeat content. (B) The distribution and composition of repeat elements in *P*. *musae*, *P*. *eumusae*, and *P*. *fijiensis*. The proportion (%) and size (Mb) of each individual class of repeat elements are indicated. The three species differ in their composition and proportion of the different classes or repeat elements.

Subsequent analysis of the single-copy and repeat regions, in which *k*-mer frequencies falling between the boundaries of the peak region were considered as single-copy regions, indicated that the differences in genome sizes are essentially due to differences in repeat content. Indeed, while 31.3 Mb (37.8%), 34.6 Mb (64.3%), and 36.4 Mb (49%) of the genomes of *P*. *musae*, *P*. *eumusae*, and *P*. *fijiensis*, respectively, are classified as single-copy regions, in contrast, the amount of repetitive content and unassembled sequences ranges from 51.5 Mb (62.2%) in *P*. *musae*, to 19.2 Mb (35.7%) in *P*. *eumusae*, and 37.7 Mb (51.0%) in *P*. *fijiensis*, thus showing that, as with other Dothideomycetes [15, 16], repeat content is highly variable and plays the largest role in determining genome sizes (Tables 1 and S1, Fig 2A).

The high number of repetitive sequences also impeded assembly efforts, as *de novo* assembly of the NGS reads produced a highly scaffolded genome of 60.4 Mb with 3331 scaffolds for *P*. *musae* and 47.1 Mb with 2626 scaffolds for *P*. *eumusae* (Tables 1 and S1). Average contig length was 18.1 Kb and 17.9 Kb for *P*. *musae* and *P*. *eumusae*, respectively, while the N50 size of the genome scaffolds was 0.4 Mb for *P*. *musae* and 0.16 Mb for *P*. *eumusae* (S2 Fig). Given the high sequencing depth of 100-150x that is considered sufficient to cover the breadth of protein-coding exons, the apparent discrepancy between the final assembly sizes and the genome sizes estimated by the *k-*mer distribution analysis, can be attributed to the high repeat content, which prevented the complete assembly of the repeat-rich regions and led to highly fragmented genome assemblies.

In spite of the fragmented genome assemblies, analysis of syntenic relationships in scaffold alignments between pairs of the three species, revealed high levels of localized conservation of gene content, order and orientation in most identified syntenic blocks. In pairwise comparisons of scaffolds larger than 200 kb in size from *P*. *musae* and *P*. *eumusae*, such regions of co-linearity were occasionally extended along the length of entire scaffolds in the form of segmental and tandemly repeated blocks of synteny, indicating the presence of “broken” or “segmented” macrosynteny (S3A Fig). For example, this was the case with scaffold number 2 (223.5 kb), 3 (225.7 kb), 6 (233.5 kb), and 9 (209.4 kb) of *P*. *musae* that showed almost perfect but broken macrosynteny to scaffolds in *P*. *eumusae*. However, the signature of macrosynteny was eroded in other scaffold alignments between the two species, as synteny was restricted to interspersed and very short genomic segments, as in alignments of scaffolds number 5, 7 and 11 from *P*. *musae* to those of *P*. *eumusae*. In a similar way, analysis of the pairwise syntenic relations between scaffolds in *P*. *musae* and *P*. *eumusae* larger than 200 kb in size, on one hand, and scaffolds in *P*. *fijiensis*, on the other, revealed an analogous pattern of broken macrosynteny, as stretches of interspersed co-linearity occasionally combined with intra-chromosomal inversions were frequently observed (S3B and S3C Fig, S1 Text). Although difficult to infer with certainty, due to the highly fragmented genome assemblies, overall the scaffold alignments suggest a pattern of broken or segmented macrosynteny among the three primary agents of the Sigatoka disease complex, which could possibly be driven by the lineage-specific proliferation of repetitive elements in each species and other genomic rearrangements. This pattern is different from the mesosynteny that is usually observed in genome-wide synteny alignments between more distantly related species of Dothideomycetes [17].

To further investigate the impact of the fragmented assemblies on gene identification and to assess the completeness of the assemblies with regard to gene content, we used the CEGMA pipeline to match them against a set of 248 core eukaryotic gene (CEG) families that are highly conserved across nearly all eukaryotes [18, 19]. For the analysis, the CEG families were classified into four groups (Groups 1-to-4) based on the degree of protein sequence conservation across eukaryotes, ranging from low (Group 1), to high (Group 4) (S4 Fig). Overall, CEG completeness ratios were slightly higher for *P*. *eumusae* (96.2, 95.5, 95.1, and 94.6%, respectively) as compared to *P*. *musae* (96.2, 90.1, 87.7, and 96.9%, respectively), and *P*. *fijiensis* (94.7, 92.9, 95.9, and 97.7%, respectively) for nearly all four groups. Nonetheless, all three species had completeness ratios comparable to those previously reported for other fungal genome sequencing projects [19], and thus the produced genome assemblies for *P*. *eumusae* and *P*. *musae* should cover almost the entire gene space.

### Genome size variations are strongly related to differential lineage-specific amplification of transposable elements (TEs)

The increase in genome size in species of Dothideomycetes has been frequently connected to an invasion of their genomes by TEs, consequently altering their genome structure and function and shaping their pathogenic life-styles [15, 16, 20]. Therefore, we classified and compared the diversity of TEs and other repeats present in *P*. *musae*, *P*. *eumusae*, and *P*. *fijiensis* (S2 Table) in order to understand their impact on genome organization and evolution of the three species.

Overall, TEs comprise an estimated 73.6% (21.4/29.2 Mb), 65.8% (8.3/12.6 Mb), and 79.3% (29.9/37.73 Mb) of the repetitive fractions in *P*. *musae*, *P*. *eumusae*, and *P*. *fijiensis* [14], respectively, whilst the rest of the repeat sequences can be mainly attributed to satellites, simple repeat and low complexity sequences (*Pm*: 1.6 Mb; *Pe*: 5.6 Mb; *Pf*: 0.43 Mb), unclassified repeats (*Pm*: 6.1 Mb; *Pe*: 3.7 Mb; *Pf*: 7.4 Mb), and unassembled sequences (*Pm*: 22.3 Mb; *Pe*: 6.7 Mb; *Pf*: 0.0 Mb) (Fig 1A). Class I TEs, in particular, account for the majority of the repetitive content in each genome, totaling 69.8% (20.4/29.2 Mb) in *P*. *musae*, 61.1% (7.7/12.6 Mb) in *P*. *eumusae*, and 62.3% (23.5/37.73 Mb) in *P*. *fijiensis* (Fig 2B, S2 Table). The high ratio of Class I elements in the genomes of the three species is in-between the ratio previously reported for other Dothideomycetes, such as *Fulvia fulva* (syn. *Cladosporium fulvum*, syn. *Passalora fulva*) (90.9%), *Dothistroma septosporum* (40.6%), *Plenodomis lingam* (syn. *Leptosphaeria maculans*) (83.3%), *Zymoseptoria tritici* (syn. *Mycosphaerella graminicola*) (54.4%), and others [15, 16, 21]. Within Class I TEs, LTR retrotransposons are the most numerous retroelements in all three genomes, but their fraction is much higher in *P*. *fijiensis* (21.5 Mb, 57.7%) [14] as compared to *P*. *musae* (12.5 Mb, 42.8%) and *P*. *eumusae* (5.2 Mb, 41.3%) (Fig 2B, S2 Table, S1 Text). In contrast to Class I TEs, Class II transposons are considerably less expanded in the genomes of *P*. *musae* and *P*. *eumusae*, occupying only a minor 3.8% (1.1/29.2 Mb) and 4.7% (0.6/12.6 Mb) of the repetitive fraction, respectively. In *P*. *fijiensis*, however, Class II elements are strikingly more abundant, tallying up to 17.2% (6.4/37.3 Mb) of the total repetitive fraction in this species (Fig 2B, S2 Table, S1 Text) [14].

Overall, the marked differences in the repertoire of TEs among the three species suggest that they are major contributors to genome evolution, organization, and function, also conceivably affecting their pathogenic lifestyles and contributing to the generation of new virulence specificities. In addition, such differences may also imply differences in TE activity and possibly genome defenses against mobile genetic elements, such as those mediated by repeat-induced point mutation (RIP) [22, 23],[24]. In this respect, analysis by RIPCAL [25] indicated that a larger fraction of the *P*. *fijiensis* (60.2%, 44.58 Mb) and *P*. *musae* (53.5%, 31.97 Mb) genomic sequences are under RIP as compared to *P*. *eumusae* (37.2%, 17.06 Mb) (S3 Table, S1 Text). In all three genomes RIP occurred mainly on large repeat sequences (> 500 bp) as the vast majority (~98% on average) shows signs of RIP. Such high levels of RIP in repeat sequences, although comparable to those reported for other Dothideomycetes [15, 16, 21], are inconsistent with the high density of TEs in the genomes of the three Sigatoka complex species, suggesting that RIP cannot perhaps effectively defend against TE activity (S1 Text).

### Phylogenetic reconstruction and estimation of divergence times support a recent stepwise radiation from a common ancestor

The disease chronology record suggests that *P*. *musae* was the first of the three pathogens to appear on the banana host, followed in quick succession by *P*. *fijiensis* and then *P*. *eumusae*. However, analysis of mating-type genes combined with multilocus sequence analysis of four housekeeping genes suggested a stepwise evolution from a common ancestor splitting first into *P*. *fijiensis* and then into *P*. *musae* and *P*. *eumusae* [10]. Although not conclusively determined, this analysis also suggested that these events are likely to have taken place relatively recently in the evolutionary past of the three pathogens.

**Fig 3:**
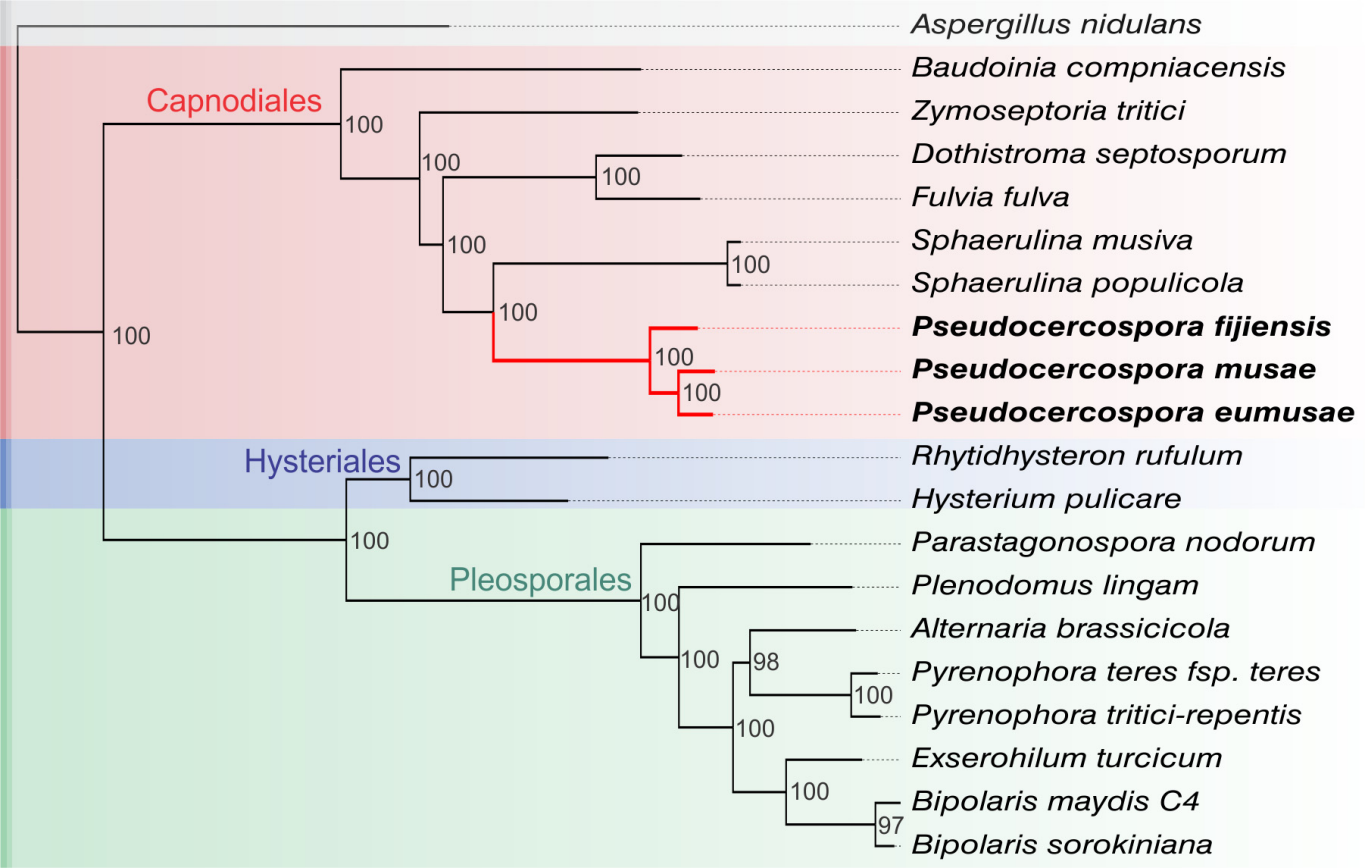
Molecular phylogeny of the three species that constitute the Sigatoka disease complex and 16 other representative Dothideomycetous fungi. The maximum likelihood (ML) tree was constructed based on a concatenated sequence alignment of 46 orthologous single-copy genes. Bootstrap values (%) are indicated next to corresponding branching nodes. *Aspergillus nidulans* (class of Eurotiomycetes) was used as an outgroup species for rooting the tree. The selected 16 representative Dothideomycete species that are included in the phylogeny fall into three major orders, i.e. Capnodiales (red), Hysteriales (blue), and Pleosporales (green). In the inferred topology *P*. *musae*, *P*. *eumusae*, and *P*. *fijiensis* are strongly clustered (bootstrap value of 100%) as a monophyletic clade within the Capnodiales, whereas *P*. *eumusae* is sister to *P*. *musae* (bootstrap value of 100%), suggesting an earlier split of *P*. *fijiensis* from the common ancestor of these two species.

To discriminate between the two opposing hypotheses and obtain a deeper insight into the species history and divergence times, we reconstructed their phylogenetic relationships based on concatenated sequences of 46 single-copy genes that are conserved across Dothideomycetes [16], and further used molecular clock analysis to obtain time estimates of their divergence [26]. In order to place the relationships among the three *Pseudocercospora* species in a broader context of other Dothideomycetes with sequenced genomes, we also incorporated 16 more species of Dothideomycetes in the analysis, including six species from the order Capnodiales, eight species from the order Pleosporales, and two species from the order Hysteriales [16]. Finally, the Eurotiomycete *Aspergillus nidulans* was used as an outgroup species for rooting the phylogenetic tree. In agreement with previous studies, Pleosporales, Hysteriales, and Capnodiales formed three tight clades within Dothideomycetes, whereas *P*. *eumusae*, *P*. *musae*, and *P*. *fijiensis* produced a highly supported (bootstrap value of 100) monophyletic clade embedded within the Capnodiales, indicating shared ancestry and a close phylogenetic relationship (Fig 3). In the inferred topology, *P*. *eumusae* is sister to *P*. *musae* and these two species together are sister to *P*. *fijiensis*, thus, conforming with the scenario inferred by other molecular markers of an ancestor successively diversifying first into *P*. *fijiensis* and then into *P*. *musae* and *P*. *eumusae* [10]. To further resolve when these radiations might have taken place and the time interval between speciation events, we used molecular clock analysis [26] to obtain estimates of divergence times. The origin of the Dothideomycetes crown group has been previously estimated to be 394–284 million years ago (MYA), during the Carboniferous period [27]. Using this time period as a calibration point and the highly-supported species tree obtained using the 46 selected genes, we estimated the divergence of the Capnodiales to be 234.2–180.2 MYA, while the radiation of Pleosporales likely took place much later at approximately 111.1–85.5 MYA and that of Hysteriales at 146.4–112.6 MYA (S5 Fig). Within the Capnodiales clade, the emergence of *Mycosphaerellaceae* is estimated to have occurred between 186.7–143.6 MYA, thus placing it almost immediately after the appearance of the Capnodiales but considerably earlier than the previously estimated 120–87 MYA [16]. This may be the result of incorporating a higher number of genes in our analysis and/or the limited sampling. Despite such discrepancies in time estimations, our results are in agreement with previous reports that support an earlier origin for the Capnodiales as compared to Pleosporales and Hysteriales [16, 28]. Within *Mycosphaerellaceae*, the last common ancestor of *P*. *eumusae*, *P*. *musae*, and *P*. *fijiensis* seems to have appeared at 146.6–112.8 MYA, splitting shortly after into *P*. *fijiensis* and the progenitor of *P*. *eumusae* and *P*. *musae* at 39.9–30.6 MYA. Finally, the split between *P*. *eumusae* and *P*. *musae* is estimated to 22.6–17.4 MYA (S5 Fig). Combined, these results validate the recent evolutionary radiation of the three species associated with the Sigatoka disease complex and, given the relatively short time interval between speciation events, further suggest high rates of diversification and consequently speciation.

### Orthology-based analysis of the species’ gene complements suggests abundant species- and lineage-specific adaptations

*De novo* genome annotations yielded 10 632 gene models for *P*. *musae* of which 10 548 represented protein-coding genes, while the rest were classified as tRNA sequences and pseudogenes. Similarly, a total of 11 173 gene models were predicted for *P*. *eumusae*, of which 11 064 represented protein-coding genes (Tables 1 and S1). The predicted proteome of *P*. *eumusae* and *P*. *musae* is slightly smaller than that of *P*. *fijiensis* (13 107) but nonetheless within the range of the proteome size reported for other plant pathogenic Dothideomycetes [15, 16]. Thus, despite the large differences in genome sizes, there is considerably less variation in protein-coding gene counts among the three species that constitute the Sigatoka disease complex.

Further annotation of the species predicted proteomes by assignment into the four major functional categories of the eukaryotic orthologous groups (KOG) database [29], indicated that a fairly similar percentage of each species proteome could be assigned to KOGs (*Pm*: 59.7%, *Pe*: 61.3%, *Pf*: 55.9%), although the total number of proteins assigned to each main category of KOG could be different among the species (S6 Fig, S1 Text). Similarly, proportionally to their proteome sizes the three species do not exhibit any significant differences in the percentage of proteins distributed across the 25 subcategories of KOG, indicating that, based on their KOG profiles, they execute a fairly similar spectrum of biological activities (S6 Fig, S1 Text).

We focused next on a comparative analysis of the protein-coding gene complements of the three species. For this purpose, reciprocal BLAST analysis (e-value: 1e-5, alignment coverage > 50%) as implemented in OrthoMCL [30] was used to retrieve the set of orthologous protein-coding gene groups among the three species and consequently determine the core, lineage- and species-specific gene families and genes. We defined “*core”* as the gene families that are shared by all three species and “*lineage-specific”* as the subset of core gene families that are not present in any other fungus. We considered “*species-specific”* as genes that are found in only one of the three species that constitute the Sigatoka disease complex, while we classified “*orphans”* as the subcategory of species-specific genes that do not have homologs in the other fungal species.

**Fig 4:**
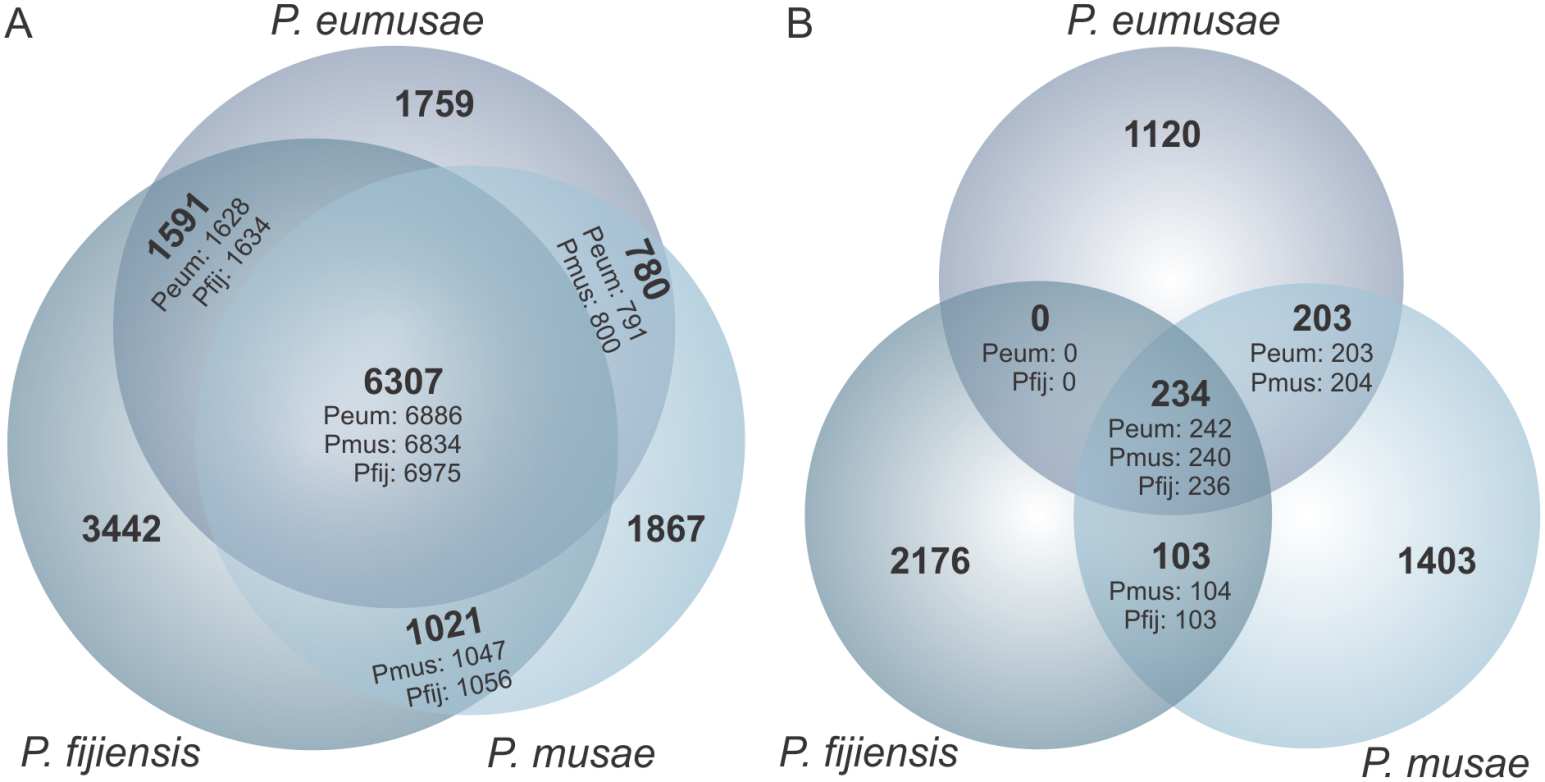
Shared and species-specific gene families and genes in *Pseudocercospora musae*, *Pseudocercospora eumusae*, and *Pseudocercospora fijiensis*. (A) Venn diagram showing the total number of species-specific genes and shared gene families among the three species, as determined by reciprocal BlastP best hit (e-value: 1e-5) analysis implemented in OrthoMCL. A larger number of species-specific genes are found in *P*. *fijiensis*, whereas more gene families are shared between *P*. *eumusae* and *P*. *fijiensis* as compared to *P*. *eumusae* and *P*. *musae*, or *P*. *musae* and *P*. *fijiensis*. (B) The Venn diagram is expanded to include a broader comparison of the three species gene content against the NCBI nr database and the JGI fungal genome database (BlastP e-value: 1e-5, alignment coverage > 50%). In both Venn diagrams, the number of genes from each species included within the pool of shared gene families is indicated at every intersection.

A total of 6307 protein-coding gene families shared by all three species were identified that represent their core proteome complement (Fig 4A, S7 Fig, S1 Text), whereas a broader BLAST-based search (e-value: 1e-5, alignment coverage > 50%) against all currently available fungal genomes in the JGI database revealed that 234 of the core families are lineage-specific to the Sigatoka species, which could facilitate virulence specifically to the banana host (Fig 4B, S8 Fig, S1 Text). A larger number of species-specific protein-coding genes were retrieved from *P*. *fijiensis* (3442/13 107, 26.2%) as compared to *P*. *eumusae* (1759/11 064, 15.9%) and *P*. *musae* (1867/10 548, 17.7%) (Fig 4A, S7 Fig, S1 Text), which is in line with the earlier branching of *P*. *fijiensis* from the last common ancestor of the three species [10]. Of the species-specific genes, 2176, 1403, and 1120 genes in *P*. *fijiensis*, *P*. *musae*, and *P*. *eumusae*, respectively, can be further classified as putative orphans, as no homologs could be identified in any other species (Fig 4B, S8 Fig, S1 Text). Taken as a whole, it is perhaps surprising to see such diversity in the species’ gene complements given the common ancestry and relatively short evolutionary distance among the three species along with the fact that they have been co-evolving with their banana host. This, in turn, implies that the evolution of virulence in these pathogens has, to an extent, been facilitated by a number of species-specific adaptations.

### Pairwise analyses of gene copy-number variations (CNV) indicate that *P*. *fijiensi*s and *P*. *eumusae* share a parallel pattern of expansions and contractions in gene families that are associated with metabolism

While species-specific acquisitions of new genes with novel functions have likely significantly contributed to the phenotypic variation among the three species, changes in gene family sizes as a result of gene duplication, loss, or elevated sequence diversification are also a major evolutionary force that could have further fostered the shifts in virulence spectra. The pairwise comparisons of gene content, for example, showed that the number of gene families shared exclusively between *P*. *fijiensis* and *P*. *eumusae* (*n* = 1591) is much larger than the number of gene families shared only between *P*. *fijiensis* and *P*. *musae* (*n =* 1021) (Fig 4A, S7 Fig). This was rather surprising as it suggests that the evolutionary distance between *P*. *fijiensis* and *P*. *eumusae* is shorter than the one between *P*. *fijiensis* and *P*. *musae*. Alternatively, it could be that *P*. *fijiensis* and *P*. *eumusae* share more similar patterns of duplications and losses in gene families that were inherited from the common ancestor of the three species. If true, it is conceivable that the evolution of virulence in *P*. *fijiensis* and *P*. *eumusae* may have been additionally facilitated by parallel gains and losses in specific gene families, which in turn may underlay the molecular basis of virulence in these two pathogens. To investigate this possibility, we first examined whether specific gene categories are enriched for copy number variants (CNVs) among the three species and, subsequently, whether a pattern exists on the expansion and reduction in the size of gene families with CNV among *P*. *musae*, *P*. *eumusae*, and *P*. *fijiensis* that could be linked to changes in their virulence phenotypes.

**Fig 5:**
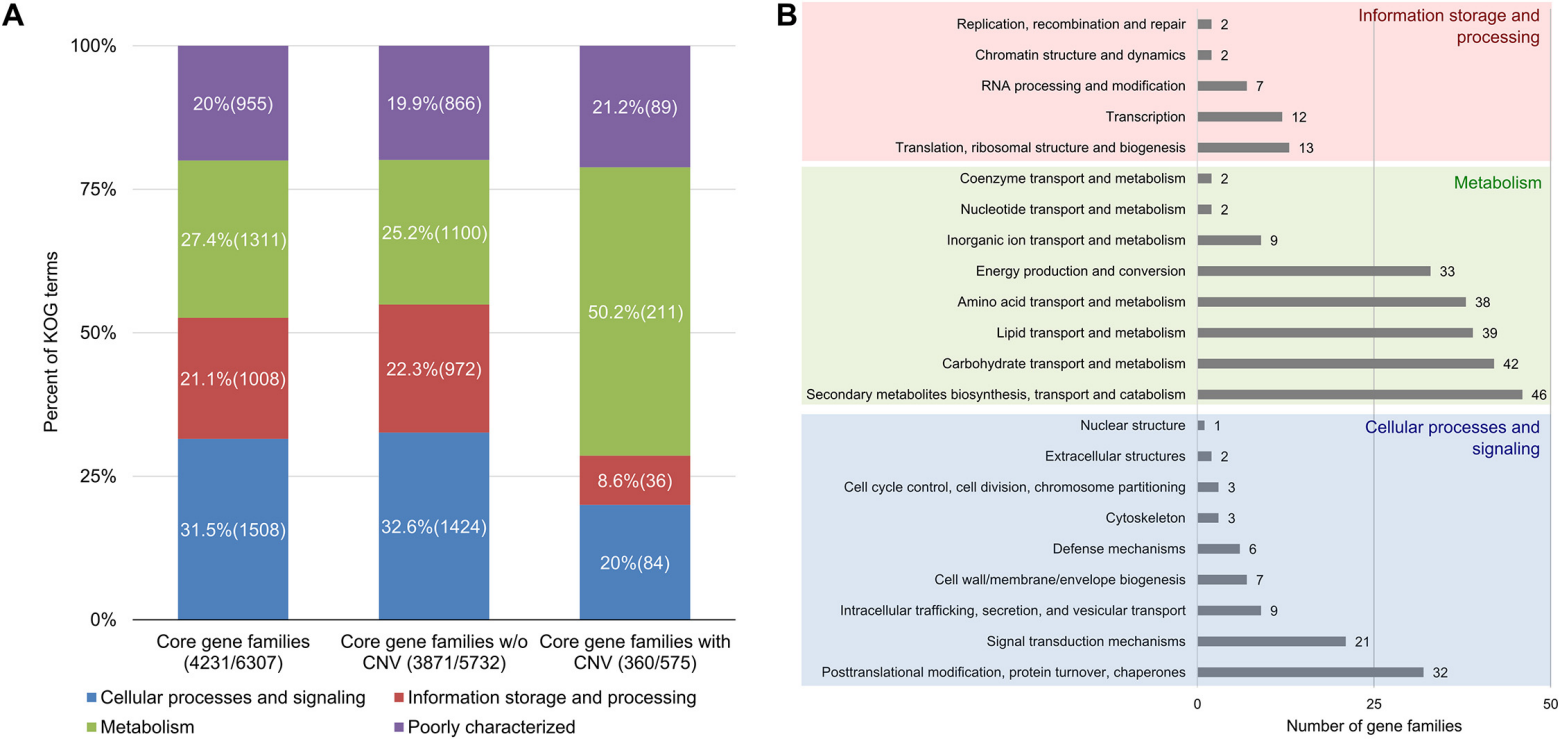
Distribution of KOG annotation profiles in core gene families with or without copy number variation (CNV) among *Pseudocercospora musae*, *Pseudocercospora eumusae*, and *Pseudocercospora fijiensis*. (A) Plotted in the different segments of the stacked bars is the percent of KOG terms assigned to core gene families (column 1), core gene families without (w/o) copy number variation (CNV) (column 2), and the core gene families with CNV (column 3), for each of the main functional categories of KOG (Cellular processes and signaling: blue; Information storage and processing: red; Metabolism: green; and Poorly characterized: purple). The number in the parenthesis of each segment in the columns refers to the number of KOG terms assigned to the gene families for each specific functional category of KOG. The first number in the X-axis label of each comparison refers to the total number of gene families with assigned KOG terms, whereas the second number refers to the total number of gene families in each comparison compartment. A high fraction (211, 50.2%) of the total number of 420 KOG terms that were collectivity assigned to gene families with CNV was ascribed to metabolism. (B) The number of gene families with CNV assigned to each subcategory of KOG. A high number of gene families (46) is associated with biosynthesis of secondary metabolites, transoport and catabolism, as well as carbohydrate transport and metabolism (42), lipid transport and metabolism (39), and amino acid transport and metabolism (38). Note that because some gene families receiving KOG annotations could be assigned to more than one functional categories of KOG, the number of KOG terms in this case is equivalent to the number of gene families.

Of the 6307 core gene families shared by the three species, 5583 are single-copy families. The remaining 724 correspond to multi-copy gene families, of which 575 display copy number variations (CNV) among the three species. Functional annotations revealed that while the KOG-based distribution of the 5732 gene families without CNV follows a similar pattern to that obtained for the core proteome of the species (Fig 5A), in contrast, gene families with CNV are significantly enriched in genes encoding for proteins that are involved in metabolism (211 KOG terms, 190/575 gene families, 33%) rather than cellular processes and signaling (84 KOG terms, 76/575 gene families, 13.2%), or information storage and processing (36 KOG terms, 35/575 gene families, 5.7%) (Fig 5A). Further characterization of the gene families with CNV, according to the subcategories of KOG, showed that most could be classified in secondary metabolite biosynthesis transport and catabolism (46 gene families), followed by carbohydrate metabolism and transport (42 gene families), and finally lipid transport and metabolism (39 gene families) (Fig 5B). Taken together, the above results indicate that changes in gene family sizes across the three species are not selectively neutral and uniform for all biological processes, but largely affect genes involved in metabolic processes. Such a functional bias in gene categories enriched for CNVs implies an association of virulence with altered metabolism in the three pathogens, perhaps for enhanced uptake and utilization of the nutrients obtained from the host and/or production of certain secondary metabolites.

**Fig 6:**
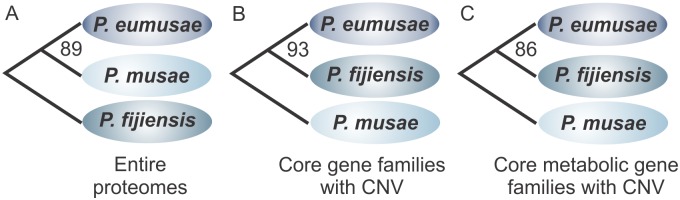
Hierarchical clustering of *Pseudocercospora musae*, *Pseudocercospora eumusae*, and *Pseudocercospora fijiensis* based on copy number changes in different groups of KOG gene families. Hierarchical clustering of the species based on (A) the KOG distribution profile (i.e. the number of genes assigned to each category of KOG) of their entire proteomes, (B) the KOG distribution profiles of the 575 core gene families with copy number variation (CNV), and (C) a subset of 190 core gene families with CNV that are predicted to be involved in metabolism based on KOG assignments. The reliability of the clustering patterns was assessed by bootstrap tests (1000 replicates) and obtained bootstrap values are indicated next to their corresponding branching nodes. While clustering of the species based on the KOG distribution profiles of the entire proteomes follows a pattern that is respective of the their phylogenetic relations (Fig 2), clustering of the species based on the KOG profiles of core gene families with CNV or their subset of gene families involved in metabolism, indicates a swapped topology in which *P*. *eumusae* is clustered together with *P*. *fijiensis* suggesting that these two species share a more similar pattern of gene family expansions and contractions.

To further elucidate whether a causal relationship exists between CNV in genes involved in metabolism and the species virulence phenotypes, we performed hierarchical clustering based on the KOG distribution profiles (i.e. by enumerating the number of genes assigned to each category of KOG) of the species entire proteomes and compared it with the species hierarchical clustering based on the KOG distribution profiles of their core gene families with CNV. When clustering was performed using the species entire proteomes, then the obtained tree topology was reflective of their evolutionary relationships, with *P*. *musae* and *P*. *eumusae* clustering together as a monophyletic group (Fig 6A). In contrast, hierarchical clustering of the species based on the KOG distribution profiles of the 575 core gene families with CNV (Fig 6B) or the subset of 190 gene families with CNV that are predicted to be involved in metabolism (Fig 6C), returned swapped topologies in which *P*. *fijiensis* clustered with *P*. *eumusae* as a monophyletic group with strong supporting bootstrap values (93 and 86, respectively). These clustering patterns were consistent and irrespective of distance measure and clustering algorithm used, suggesting that *P*. *fijiensis* and *P*. *eumusae* share a more congruent pattern of gene family expansions and contractions as compared to *P*. *eumusae* and *P*. *musae* or *P*. *fijiensis* and *P*. *musae*. Similar results were also obtained when the above analysis was expanded to include gene families that are shared by at least two of the species but not necessarily the third one, in which case pairwise comparisons showed that a significantly higher number of the gene families had exactly the same copy number shared between *P*. *eumusae* and *P*. *fijiensis* (1742 gene families), rather than between *P*. *musae* and *P*. *fijiensis* (1127 gene families) or between *P*. *musae* and *P*. *eumusae* (945 gene families) (S9 Fig, S1 Text). Moreover, the analysis of CNV in the metabolic gene families of the nine Capnodiales species that were previously used for phylogenetic reconstruction and estimation of divergence times (Fig 3, S5 Fig), further supported that the clustering of *P*. *fijiensis* together with *P*. *eumusae*, when considering changes in metabolism, is likely due to parallel expansions and contractions in these two species rather than changes that took place solely in *P*. *musae* (S10 Fig, S1 Text).

Although the analysis performed based on the KOG annotations of the species entire proteomes indicated that the more virulent *P*. *eumusae* and *P*. *fijiensis* share complementary patterns of expansions and contractions in core gene families related to metabolism, it does not provide any information regarding the metabolic pathways that these gene families are involved in. To investigate which pathways are likely to have been affected by parallel changes in the two more virulent species, we performed a genome-wide GO (Gene Ontology)-based analysis and identified GO terms that support the clustering of *P*. *eumusae* with *P*. *fijiensis* (S11 Fig, S1 Text). The analysis indicated that GO terms associated with metabolic processes (GO: 0008152) and particularly regulation of metabolic processes (GO: 0019222) and cellular metabolic processes (GO: 0044237) (S12 Fig, S1 Text) are those contributing the most to the clustering of *P*. *eumusae* together with *P*. *fijiensis* when considering changes in the species proteome, thus further corroborating the KOG-based analysis.

Taken together, the above results indicate that changes in gene family sizes among the three species that constitute the Sigatoka disease complex have not been selectively neutral but are more respectful of the species virulence profiles rather than their evolutionary relationships. This implies that, next to species-specific evolutionary adaptations, the evolution of virulence in the three pathogens has also been driven by recurrent genomic changes on particular molecular pathways. Among the evolutionary mechanisms shared by the more virulent *P*. *fijiensis* and *P*. *eumusae* are matched changes in the size of families related to metabolism that could potentially translate into a higher efficiency of nutrient uptake and utilization. Although speculative, the annotation of the species-specific genes shows that they mostly encode for novel proteins with unknown function, suggesting that they might be virulence-associated genes with a role in overcoming or evading the host immune system.

### Annotation of carbohydrate-active enzymes (CAZymes) and plant cell wall degrading enzymes (PCWDEs) suggests that *P*. *eumusae* and *P*. *fijiensis* also share more similar CAZyme profiles as compared to *P*. *musae*

The fairly coordinated changes in the size and range of metabolic gene families shared between *P*. *fijiensis* and *P*. *eumusae* suggests that many of these families could have played a significant role in the evolution of virulence in these two pathogens. However, next to nutrient uptake and utilization, nutrient acquisition through the enzymatic degradation of plant polysaccharides is also an important aspect of pathogenesis that promotes host colonization and infection.

To assess the ability of *P*. *musae*, *P*. *eumusae*, and *P*. *fijiensis* to degrade and metabolize different polysaccharides, we annotated and contrasted their repertoires of putative carbohydrate-active enzymes (CAZymes), with an emphasis on characterizing enzymes that are involved in the breakdown of plant cell walls (PCWs). In order to identify any features specific to the three banana pathogens we, additionally compared the CAZyomes of the three Sigatoka species to the ones of 16 other Dothideomycetous fungi with different nutritional lifestyles and host specificities [15, 16] (S1 Text).

Our CAZy annotations identified a total of 490, 501, and 516 CAZyme modules from all six major CAzyme superfamilies in the predicted proteomes of *P*. *musae*, *P*. *eumusae*, and *P*. *fijiensis*, respectively (S4 Table, S13 and S14 Figs, S1 Text). Plant cell wall degrading enzymes (PCWDEs), in particular, are the most abundant in the three species, accounting approximately for a quarter of their CAZyomes (*Pm*: 119/490, 24.3%; *Pe*: 125/501, 25.0%; *Pf*: 130/516, 25.2%). The majority of PCWDEs are putatively directed towards the degradation of hemicellulose (*Pm*: 54.6%, *Pe*: 55.2%, *Pf*: 53.1%), followed by the decomposition of hemicellulose-pectin complexes (*Pm*: 21.0%, *Pe*: 22.4%, *Pf*: 21.5%), pectin (*Pm*: 21.0%, *Pe*: 20.8%, *Pf*: 22.3%), and cellulose (*Pm*: 3.4%, *Pe*: 1.6%, *Pf*: 3.1%) (S5 Table, S15 Fig, S1 Text). The higher number of hemicellulases in the three Sigatoka species is not unusual among plant pathogenic fungi [16, 31], whereas comparative analysis with the group of 16 Dothideomycetous fungi included in this study did not, based on Mann-Whitney U tests, identify any significant differences in the abundance of PCWDEs present in these groups. However, significant differences were detected at the individual CAZYme family level, including when the CAZyme distribution profiles of the three Sigatoka species were compared with the distribution profiles of five hemibiotrophic fungi from the Capnodiales clade that were included in the group of 16 Dothideomycetes (S6 Table, S1 Text). Such differences could reflect an evolutionary adaptation of *P*. *musae*, *P*. *eumusae*, and *P*. *fijiensis* to their banana host and the fine-tuning of their CAZyme repertoire for a better exploitation of the polysaccharide resources available in this host.

**Fig 7:**
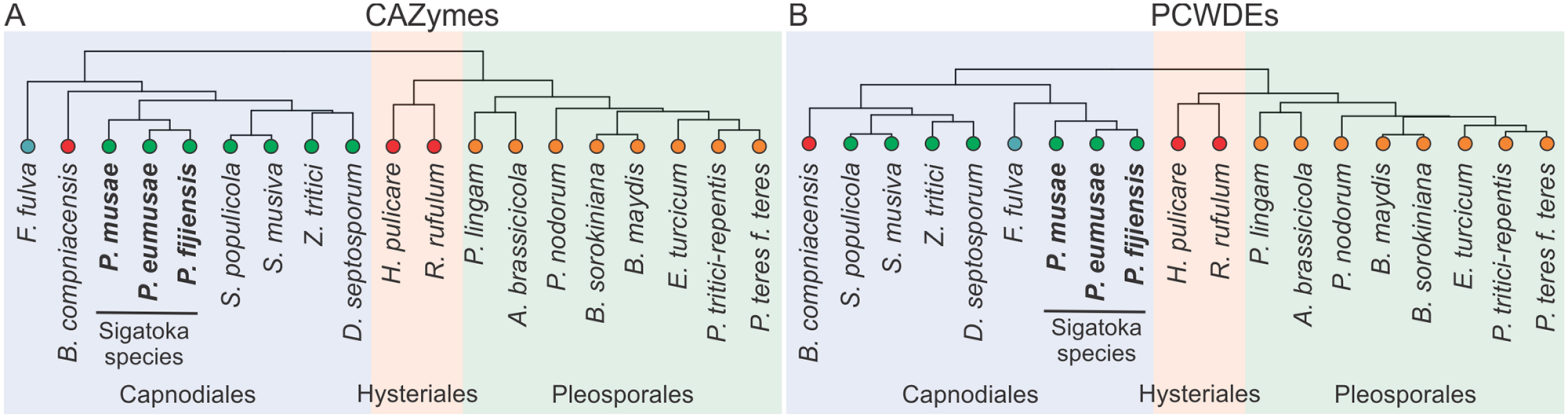
Hierarchical clustering of *Pseudocercospora musae*, *Pseudocercospora eumusae*, *Pseudocercospora fijiensis*, and 16 other representative Dothideomycete fungi with different nutritional lifestyles, based on copy number changes in carbohydrate-active enzyme (CAZyme) families or the subset of plant cell wall degrading enzymes (PCWDEs). The selected 16 representative Dothideomycete species that are included in the analysis fall into three major orders: Capnodiales (red), Hysteriales (blue), and Pleosporales (green). The nutritional lifestyle of each species is indicated by a colored dot next to each species name: biotrophs (blue), hemi-biotrophs (green), necrotrophs (yellow), saprophytes (red). (A) Hierarchical clustering of the species based on their total CAzyme distribution profile (i.e. the number of genes assigned to each CAZyme family) (B) Hierarchical clustering of the species based on their distribution profile for CAZyme families related to plant cell wall degradation. In both cases, clustering supported a swapped topology in which *P*. *eumusae* is clustered together with *P*. *fijiensis*, suggesting that these two species share a more similar pattern of gene family expansions and contractions in CAZymes and PCWDEs in particular.

Although the three banana pathogens share similar overall numbers in PCWDEs, they do display some differences at the individual family level, perhaps as a result of the enzymatic redundancy exhibited among many of the CAZy families (S6 Table, S16 Fig, S1 Text). Notably, hierarchical clustering of the species based on the distribution profiles in individual CAzyme families of their entire CAZYomes or arsenal of PCWDEs, resulted once more in *P*. *eumusae* grouping with *P*. *fijiensis* rather than *P*. *musae*, as expected based on the phylogenetic placement of the three species. This indicates that *P*. *eumusae* and *P*. *fijiensis* share complementary patterns of expansions and contractions in CAZymes (Fig 7A) and PCWDEs (Fig 7B) more specifically. Such coherent changes between *P*. *eumusae* and *P*. *fijiensis* in the size of gene families related to nutrient acquisition could reflect evolutionary changes that underlie a more effective exploitation of the banana host. Thus, in addition to parallel adaptations for nutrient utilization, *P*. *eumusae* and *P*. *fijiensis* seem to have evolved more similar mechanisms for nutrient acquisition as well. Taken together, based on their overall arsenal of CAZymes, the three species likely do not exhibit substantially large differences in their ability to break-down and metabolize different types of plant cell material, although, given their differences at the individual CAZy family level, they may differ in the efficiency by which they hydrolyze different types of polysaccharides.

### The three banana pathogens have putatively the capacity to produce a diverse and only partially overlapping array of secondary metabolites (SMs)

The production of phytotoxic metabolites by the three pathogens that constitute the Sigatoka disease complex has long been known, but whether these play a pivotal or rather secondary role in the interaction of the pathogens with their *Musa* host remains in question [32–37]. To obtain an insight into the commonalities and differences of the arsenal of phyto- and mycotoxins that may be produced by the three banana pathogens, we performed an inventory of the genes encoding the four core enzyme types that catalyze the first committed step in the biosynthesis of the major secondary metabolite (SM) classes found in fungi, namely the non-ribosomal peptide synthases (NRPSs), the polyketide synthases (PKSs), the terpene synthases (TSs), and the dimethylallyl tryptophan synthases (DMATSs) (S1 Text) [38].

Despite their hemibiotrophic lifestyle, 28, 27, and 21 genes encoding core SM enzymes were identified in the genomes of *P*. *musae*, *P*. *eumusae*, and *P*. *fijiensis*, respectively, indicating that the three pathogens have the ability to produce diverse SMs. The majority of core enzymes in all three species are predicted as PKSs (7 in *Pm*: PksA-to-PksG, 10 in *Pe*: Pks1-to-Pks10, and 7 in *Pf*: PksI-to-PksVII), followed by NRPSs (10 in *Pm*: NpsA-to-NpsK, 7 in *Pe*: Nps1-to-Nps6, and 8 in *Pf*: NpsI-to-NpsVII) or hybrid PKS-NRPSs (1 in *Pm*: PksNpsA, 2 in *Pe*: PksNps1 and PksNps2, and 2 in *Pf*: PksNpsI and PksNpsII), and finally TSs (5 in *Pm*: TsA-to-TsG, 5 in *Pe*: Ts1-to-Ts5, and 4 in *Pf*: TsI-to-TsIV) (S7 Table, S1 Text). No DMATs were detected in any of the three species. The number and type of core SM genes predicted in the genomes of the three banana pathogens are comparable to those reported previously for other species of Capnodiales, including the close-related tomato pathogen *F*. *fulva*, the wheat pathogen *Z*. *tritici*, and the poplar pathogen *S*. *populicola* [15, 16]. Furthermore, phylogenetic analysis with other fungal core SM enzymes [39–40], showed that most core enzymes from the three banana pathogens could be clustered with high support (ML bootstrap values ≥80%) with enzymes that are involved in the biosynthesis of known phyto- and mycotoxins in other fungi, and thus could be involved in the production of structural analogs with matching backbones. Among others, these include core enzymes that are involved in the biosynthesis of notorious mycotoxins, such as fumonisins, and light-activated phytotoxins, such as elsinochrome and cercosporin, thus corroborating earlier experimental findings suggesting the involvement of photoactivated toxins in the pathogenesis of the three Sigatoka species (S17, S18 and S19 Figs, S1 Text). Overall, the annotation and analysis of core SM enzymes in *P*. *musae*, *P*. *eumusae*, and *P*. *fijiensis* showed that although the three pathogens share some orthologous core enzymes, they differ in the arsenal of SMs that they potentially produce, some of which could bare structural similarity in their backbone structure to already characterized phyto- and mycotoxins (S1 Text).

### Effector annotations indicate that the three pathogens exhibit overlapping but still very dissimilar repertoires of candidate effectors

To gain a deeper insight into the pathogenic potential of the three species that constitute the Sigatoka disease complex, we characterized their secretomes (S20 Fig), placing an emphasis on identifying and comparing their repertoires of candidate effectors. A total of 612, 638, and 584 secreted proteins, of which 110, 112, and 105 represented putative effector proteins, were predicted in the genomes *P*. *musae*, *P*. *eumusae*, and *P*. *fijiensis*, respectively, indicating that the three species employ secretome and effector arsenals of comparable size to those of most other hemi-biotrophic fungi (Mann-Whitney U test, *P-*value = 0.01) (S8 Table, S1 Text).

Clustering by OrthoMCL indicated that, on average, ~50% of the effectors in each species could be regarded as species-specific (*Pm*: 48 effectors, *Pe*: 54 effectors, *Pf*: 61 effectors), while a broader BlastP-based search for homologs in the NCBI nr database and the JGI fungal genome database, suggested that a large number of the species-specific effectors can be further classified as orphans (*Pm*: 30 effectors, *Pe*: 27 effectors, *Pf*: 39 effectors) (S9 Table; S21A Fig). To further confirm that some of the differences in the effector repertoires of the three species are species- rather than strain-specific, we randomly selected a set of 12 species-specific or orphan effectors from each of the three pathogens and used PCR, with primers designed within the effectors’ genes coding sequences, to amplify them from seven isolates of each species. PCR and subsequent sequencing analysis of the amplified products confirmed that the 12 randomly selected species-specific or orphan effectors were both conserved within their species of origin and absent in the other two species (S10 Table; S22 Fig).

The clustering analysis also indicated that more effector families are shared between *P*. *eumusae* and *P*. *musae* (*n = 23*) as compared to *P*. *fijiensis* and *P*. *eumusae* (*n* = 9) or *P*. *fijiensis* and *P*. *musae* (*n = 10*). Thus, unlike changes in the metabolome and CAZyome of the species, clustering of the species based on the effector repertoires is more respectful of their evolutionary relationships rather than their virulence on their *Musa* host. Moreover, 22 core effector families shared by all three pathogens were identified, seven of which can be regarded as lineage-specific, as they were only present in the three pathogens that constitute the Sigatoka disease complex and none of the other fungal species (S9 Table, S21A Fig, S1 Text). Among the core effectors shared by the three banana pathogens and other fungi are three paralogs of Ecp2 (i.e. Ecp2-1, Ecp2-2, and Ecp2-3) [41] and homologs of the *F*. *fulva* Ecp6 [42] and Avr4 [43] chitin-binding effectors (S9 Table, S1 Text). Overall, the analysis suggests that the three banana pathogens, despite their very close evolutionary relationships, common host and infection biology, exhibit a considerably diverse arsenal of effector proteins that could have contributed to their differences in virulence (S1 Text).

### Orthologous genes shared by the three species are primarily under purifying selection

Next to gross genomic changes in content and architecture, the identification of the genes and genetic pathways most affected by selection during speciation is essential for both understanding the evolutionary history of fungal plant pathogens, as well as for finding important traits that contribute to phenotypic diversity and disease [44]. Our previous analysis of gene content indicated a functional bias in the pattern of expansions and contractions in families related to metabolism and enzymatic degradation of PCWs. Here we examined whether, within the group of orthologous genes shared by the three species, similar patterns of elevated selection pressure could be observed among the different functional categories of gene families. Along the same lines, we also investigated whether putative effectors and other secreted proteins shared by the three pathogens show evidence of positive selection or higher evolutionary rates. If the case, such findings could suggest that next to changes in gene content, positive selection has also contributed to the phenotypic divergence of the three species. For the analysis of selection pressures, we used the maximum likelihood method implemented in the Codeml program of PAML [45] to calculate the ratio of non-synonymous (*dN*) to synonymous *(dS*) substitutions for all between species pairwise comparisons of the 6307 orthologous gene families shared by them. For any given pair *dN*/*dS* >1 is suggestive of positive selection, while *dN*/*dS* <1 indicates purifying selection. As different parts of the proteome and functional categories of genes could experience significant differences in selection pressure, *dN*/*dS* ratios were also examined separately for different gene families and subgroups of genes, including, for example, genes encoding secreted or non-secreted proteins and genes encoding putative effectors or secreted proteins excluding the effectors.

*dN*/*dS* ratios for the entire set of orthologous genes shared by the three species ranged from 0.00–2.63, while the median *dN*/*dS* value is very low (0.1) indicating that the vast majority of the orthologous gene pairs appear to be under purifying selection (S23 Fig). The subgroup of genes encoding for secreted proteins displayed slightly higher evolutionary rates as compared to genes encoding non-secreted proteins, although median *dN*/*dS* values for each specific subgroup remained very low (0.128 and 0.099, respectively). Also, within secreted proteins, putative effector encoding genes have experienced relatively higher levels of adaptive evolution (median *dN*/*dS* value of 0.214) as compared to the pool of secreted but non-effector encoding genes (median *dN*/*dS* value of 0.124). However, caution is needed when comparing evolutionary rates among the different subgroups, as the sample sizes used in calculations of median *dN*/*dS* values varied considerably among them. In this respect, median *dN*/*dS* values were lower for all groups than mean values, suggesting a skewed distribution and an excess of proteins with evolutionary rates lower than the average. A search within each group for orthologous pairs with *dN*/*dS* >1 identified only a single core effector (Avr4-2), which however did not receive any statistical support (*P* = 0.233, Fisher’s exact test) for being positively selected, and 27 non-secreted proteins of which only four received statistical support at the 0.05 level for being positively selected (S11 Table). Of these four proteins, one could be annotated as a sulfatase based on Pfam and GO annotations, while none of the other three proteins received any functional annotations. In addition, examination of *dN*/*dS* rate ratios in orthologous pairs of protein-coding genes representing the different functional categories of KOG did not indicate any significant differences in evolutionary rates between the group of genes encoding for proteins that are involved in metabolism (median *dN*/*dS* value of 0.07) as compared to the group of genes encoding for proteins that are involved in cellular processes and signaling (median *dN*/*dS* value of 0.08), information storage and processing (median *dN*/*dS* value of 0.08), or poorly characterized ones (median *dN*/*dS* value of 0.09) (S24 Fig). Also, among the group of orthologous CAZymes that are shared by all species, we could not identify any genes as being under positive selection or an elevated *dN*/*dS* ratio for the subgroup of genes encoding PCWDEs (S25 Fig). Overall, based on a global analysis of *dN*/*dS* ratios, we identified only very few cases of positive selection in the group of orthologous genes shared by the three species. Instead, we observed abundant purifying selection, suggesting that the conserved between the species proteome has likely played a less significant role in the phenotypic diversification among the three species.

### Concluding remarks

Currently, the Sigatoka disease complex of banana, caused by the closely related Dothideomycetes (Ascomycetes), *P*. *musae*, *P*. *eumusae*, and *P*. *fijiensis*, is the most devastating disease on bananas, reducing yields by more than 40% [3–5]. The three species have surfaced as destructive pathogens on bananas during the last century and although they have evolved from a recent common ancestor, clear differences in virulence exist amongst them that correlate with the time of their appearance [5–7]. Within this complex, *P*. *musae* was the first of the three pathogens to be recorded on banana, although black Sigatoka caused by *P*. *fijiensis* is currently the major agronomic constraint for banana production, necessitating over 50 contact fungicide applications per year for its control. It is also one of the most marked examples of a recent pandemic in the plant kingdom and, considering the importance of banana as a staple food crop, a serious threat to global food security. Despite its aggressiveness, over the last decade black Sigatoka is gradually replaced by *P*. *eumusae*, which appears to be equally, if not more, aggressive and resilient than *P*. *fijiensis* [5–7]. Thus, there is an urgent need to understand the pathobiology of these species in order to safeguard banana production for the future [1, 4]. The relative short evolutionary distance of the three Sigatoka pathogens and their differences in virulence that broadly parallel their historical record of appearance, offer an excellent opportunity to examine the genomic changes associated with increased virulence, speciation, and specialization of parasites on their host. Evolution of microbial virulence and the genetics of host-adaptation is a highly active and competitive field but there is only a limited knowledge about these processes in plant pathogenic fungi, as compared to bacteria and oomycetes,

Here, we have sequenced the genomes of *P*. *musae* and *P*. *eumusae*, and compared them with the available genome sequence of *P*. *fijiensis* [14] in order to first understand the nature, diversity and extent of genomic modifications associated with shifts in their virulence spectra on banana after speciation and second, to determine whether some of the changes and evolutionary processes are recurrent across the species, and thus predictable. A critical question in fungal evolutionary biology is whether speciation and diversification of virulence is mainly facilitated by adaptive evolution of the core genome or through species-specific gene acquisitions. Our analysis showed that speciation has largely altered both the genome architecture and composition of the three species. More specifically, comparative analysis of genome architectures revealed marked differences in genome sizes among the three species that positively correlate with different rates of TE, and especially LTR-retrotransposon, accumulation and retention. The three species also show marked differences in the type of TEs that they maintain in their genomes, including in the ratios of Class I and Class II TEs. As these two classes of transposons leave different imprints on coding and non-coding DNA sequences [46], they may have also differentially impacted genome evolution and innovation in the species. The differential invasion of the genomes by TEs has also likely contributed to chromosomal rearrangements and the breakdown of macrosynteny among the three species, consequently accelerating the process of speciation and diversification. Analysis of gene content showed that although the three species retain a similar in size predicted arsenal of protein-coding genes, they exhibit considerable differences in their gene composition, suggesting that the evolution of virulence in these pathogens has, to an extent, been facilitated by a number of species-specific adaptations. This is particularly true for putative virulence associated genes, such as those encoding for effectors, as ~50% of the effectors in each species could be regarded as species-specific. Notably, of the core effectors, seven were found only in the three pathogens that constitute the Sigatoka disease complex and these might play an essential role in the interaction with the banana host. Next to overcoming the host immune system, the capacity for metabolic adaptation, in terms of acquiring and exploiting the host nutrient resources has also likely played a major role in the evolution of virulence in the three species. In this respect, metabolic streamlining in *P*. *fijiensis* and *P*. *eumusae* through independent but parallel expansions and contractions in gene families that are associated with metabolism and PCWDEs may have contributed to the increased virulence of these two species on the banana host. Such parallel changes in the two most aggressive species suggest that they may represent *molecular fingerprints* of adaptation to the banana host. Thus, next to species-specific adaptations, convergent evolution in specific molecular pathways seems to have facilitated the evolution of higher virulence in *P*. *eumusae* and *P*. *fijiensis*.

## Materials and Methods

### Genome sequencing and assembly

The genomes of *P*. *musae* (strain CBS116634) and *P*. *eumusae* (strain CBS114824) were sequenced by the UC Davis Genome Sequencing Core facility using the Illumina HiSeq technology (150 bp pair-end reads). A total of 22.7 and 26.0 million pair-end reads were obtained for *P*. *musae* and *P*. *eumusae*, respectively. The read quality was assessed by FastQC [47] and low quality reads and/or bases were trimmed using Trim Galore [48]. The high quality reads were assembled using different assembly software, including SoapDenovo2 [49], SPAdes [50], and ABySS [51], and different *k*-mer sizes (*k* = 55, 77, 99, and 121) and the assembly with the highest assembly qualities in terms of N50 value and assembly size was selected and merged by GAM-NGS [52] to obtain a consensus assembly for each species. The consensus assembly was scaffolded by SSPACE [53] and the remaining gaps in the scaffolds were closed by GapFiller [54]. The estimated genome coverage is 112x in *P*. *musae* and 165x in *P*. *eumusae*. The *P*. *musae* (GenBank: LFZO01000000) and *P*. *eumusae* (GenBank: LFZN01000000) genomes have been deposited to DDBJ/EMBL/GenBank, whereas the genome of *P*. *fijiensis* was reported earlier (GenBank: GCA_000340215.1)[14].

### Estimation and analysis of repeat content

An estimation of the repeat content size was first performed through a calculation of *k*-mer occurrence by Jellyfish [55] using *k* = 17 bp and summarized as a histogram. The histogram was examined by custom R scripts to partition it into regions that corresponded to potential unique and repetitive fractions of the genomes, based on peak positions. The total number of *k*-mer in the unique and repetitive fractions was calculated as an estimate of the fraction size. RepeatModeler [56] incorporating RECON [57], RepeatScout [58], TRF [59], and RepeatMasker [60] was used for *de-novo* identification and modeling of the different classes of repeat families. For each species, RepeatModeler produced a library of classified putative interspersed repeats. All repeat families were compared with Repbase sequences [61] for classification. The consensus repeat element library identified in each species was fed into the downstream annotation pipeline. The genomic regions subject to repeat induced mutation (RIP) were predicted following the composite RIP index (CRI) method as described in *de Wit* et al. (2012) [15]. RIPCAL [25] and custom Perl scripts were used to analyze and annotate the genomic regions under RIP mutations. The RIPed sequences were defined according to RIP product (≤ 1.2), RIP substrate index (≤ 0.8) and composite RIP indices (≥ 1.0). A genomics region was considered as a RIPed locus when its sequence length was larger than 750 nt along with a peak CRI ≥1.5.

### Genome annotations

The *P*. *musae* and *P*. *eumusae* genomes were both annotated using the Maker2 annotation pipeline [62], which incorporated several gene model prediction programs and sequence analyses based on EST and transcriptome, to improve the quality of genome annotations. In the pipeline, RepeatMasker [60] was first used to mask the genome regions that were comprised of low-complexity repeats and interspersed repeats, based on the repeat element library produced from RepeatModeler. RepeatRunner [63] was then used to identify more divergent transposable elements and viral proteins that may have been missed by RepeatMasker. After masking the repeat elements, *ab initio* gene predictors such as SNAP [64], Augustus [65], and GeneMark-ES [66] were used for prediction of gene models in the genomes. To improve the quality of gene model prediction, we performed transcriptome sequencing (RNA-seq) of cDNA libraries representing two different *in vitro* growth conditions, i.e. growth in rich media (10 g/L Yeast extract, 30 g/L Glucose) and growth in poor media (1 g/L KH_2_PO_4_, 1g/L KNO_3_, 0.5 g/L MgSO_4_x7H_2_O, 0.5 g/L KCl, 0.5 g/L Sucrose, 0.5 g/L Glucose), using Illuimina HiSeq platform (PE100x100) in each species. The generated 26.3 and 23.6 M of pair-end reads for *P*. *musae* and *P*. *eumusae*, respectively, were *de novo* assembled by Trinity [67] and the resulting transcriptome shotgun assemblies have been deposited at DDBJ/EMBL/GenBank under the accession GDIK00000000 (*P*. *musae*: PID PRJNA289098 and *P*. *eumusae*: PID PRJNA289096). The resulted transcriptomes along with ESTs deposited in the NCBI dbEST database were used for training the gene prediction parameters. Maker2 merged all the predicted gene models from different gene predictors to generate a set of predicted gene models, which were further polished by EST and protein alignments by BLAST and Exonerate [68] to avoid spurious predicted gene models. To further improve the performance of the *de novo* gene prediction, a second round of gene predictions was conducted using the generated gene annotations as input for the training step in order to re-annotate the genomes using the Maker2 pipeline iteratively. The completeness of the genome assembly was assessed by the CEGMA pipeline [18], as indicated elsewhere [19]. Gene families were predicted using the OrthoMCL pipeline [30], which produces normalized score based on the E-values generated from an all-versus-all BLASTp analysis (1e-5 as the cutoff value) for pairs of the compared genomes. The normalized scores were fed into the MCL algorithm to classify the genes into hypothesized orthologous and paralogous gene families using a default inflation parameter of 1.5.

### Functional annotations

Functional annotations were first performed using the InterProScan pipeline [69], which compared encoded protein sequences against the PFAM [70], PROSITE [71], and ProDom [72], to identify the domains and motifs present in each gene model. Meanwhile, the associated gene ontologies and pathways of each gene model were retrieved for the InterProScan hits, when available. The second layer of genome annotations was performed by sequence similarity, by comparing the protein sequences (BLASTp) against the non-redundant protein database in NCBI and SwissProt database. A hit was considered significant when the E-value was lower than 1e-4 and the coverage higher than 50%. The eukaryotic orthologous groups of proteins (KOG) was analyzed by RPSblast [73] against the KOG database deposited in the NCBI CDD database (E-value < 1e-3). The frequency of GO terms, as identified using InterProScan was also enumerated in each species. Based on the GO frequency, we implemented the random forest method (REF) to select the GO terms that may contribute to the observed switched topology [74]. A total of 5000 trees were generated and the classification was based on combining the entire generated trees using a majority rule. The mean decrease of the Gini index (MDGI) was used to select the important GO terms. A supervised hierarchical clustering was applied for the GO terms with MDGI value > 0.01 to produce the clustering topology and heatmap. The annotation of the carbohydrate-active enzymes was performed based on a sequence search against the CAZyme Hidden Markov Models (HMM) using the HMMER3 as implemented in the dbCAN annotation server (E-value < 1e-4) [75]. The secondary metabolic genes were annotated by the AntiSMASH 2.0 pipeline [76], using the HMMs of nonribosomal polypeptide synthetase (NRPS), polyketide synthase (PKS), and terpene synthase (TPS). The prediction was further cross-validated by BLASTp analysis. The phylogenetic trees of NRPS and PKS were constructed based on the predicted NRPS and PKS sequences in *P*. *musae*, *P*. *eumusae* and *P*. *fijiensis* and an additional set of NRPS and PKS homologous sequences as described in Collemare, *et al*. [77]. For TPS, the sequences for phylogenetic tree construction was by blasting the TPS protein sequences in *P*. *musae*, *P*. *eumusae* and *P*. *fijiensis* against the SwissProt database (E-value < 1e-4 and coverage > 50%). The clustering analyses of the annotation were performed by the R ggplot [78] package. The clustering procedure was performed with different distance measures (Euclidian and Manhattan) and linkage methods (Ward, single and complete linkage methods), all of which produced a consistent clustering topology. The reliability of the topologies was assessed by multiscale bootstrap analyses by Pvclust [79] with 1000 bootstraps.

### Secretome annotations and effector predictions

The SignalP [80], TMHMM [81], TargetP [82], Phobius [83] were incorporated in the InterProScan pipeline [69] to predict the presence of signal peptide, transmembrane (TM) domains and cellular localization for each protein sequence. The WoLF PSORT program [84] was used to refine the prediction result. This information was used for secretome and effector protein prediction. Briefly, proteins with a signal peptide and a signal peptide cleavage site were predicted by SignalP (D-score > 0.5), whereas those with no TM domains or with a single TM domain within the first 40 amino acid and overlapping with the signal peptide as predicted by TMHMM and Phobius were considered as candidates of secreted proteins. The candidate proteins that were predicted by TargetP as targeted to mitochondria were also discarded. The prediction was re-examined by WoLF PSORT and those consistently predicted as secreted proteins were considered as true candidates. Finally, PredGPI [85] was used to predict the presence of GPI-anchor signal in candidate proteins, in which those with a predicted GPI-anchor signal were removed to yield the final set of secreted proteins. An effector was defined as a secreted protein with a protein length < 250 aa and a high percentage of cysteine residues in the protein that was higher than two-fold of the average cysteine percentage in all predicted proteins of each species. The amplification of selected effectors from field isolates of the three species was performed by PCR, using primers designed at the beginning and the end of the effector’s coding sequence (S12 Table). Genomic DNA of the isolates was kindly provided by Prof. Gert Kema (Wageningen University—Plant Research International, The Netherlands), Pablo Chong Aguire (Wageningen University—Plant Research International, The Netherlands), and Dr. Ewald Groenewald (CBS-KNAW Fungal Biodiversity Centre, The Netherlands). PCR conditions included an initial 95°C denaturation step for 10 minutes followed by denaturation for 15 seconds at 95°C, annealing for 30 seconds at 50–60°C depending on the effector amplified, and extension for 30 seconds at 72°C for a total of 35 cycles. PCR products were directly sequenced using the Sanger technology and sequences were aligned to the original effector sequence using the MEGA6 software [86].

### Phylogenetic placements and divergence times of the Sigatoka disease complex species

An orthoMCL [30] classification was performed on the three target species along with 17 additional species [16] to identify the homology between species following the approach as described above. A total of 46 single-copy orthologous genes were identified and used for the subsequent analysis. The amino acid sequences of these 46 genes were aligned using PRANK [87]. All the gaps present in the alignments were removed by Gblocks [88] prior to phylogenetic tree construction. Two different approaches were used for phylogenetic tree construction. First, all the genes were individually subjected to a tree construction using the maximum likelihood approach by RAxML (1000 bootstraps) and a consensus tree was produced [89]. The best evolutionary model for each alignment was determined by ProtTest [90]. Second, a maximum likelihood (ML) tree was constructed based on a concatenated alignment using PROTGAMMAWAG model with 500 rapid bootstraps. Both tree topologies were found to be consistent with each other. The divergence time of the species was estimated using the phylogenetic tree, based on the concatenated alignment by the penalized likelihood analysis, as implemented in the r8s program [26]. Based on previously published data [27], the upper and lower bound of the divergence time estimation of the root of tree (the Dothideomycetes crown group) was calibrated as 394 million years ago (MYA) and 284 MYA, respectively. The final chronogram was visualized by FigTree [91].

### Genome synteny among the Sigatoka disease complex species

The syntenic relationships among the three species were calculated using SyMap 4.0 [92]. Since the *P*. *eumusae* and *P*. *musae* genomes are more fragmented than the *P*. *fijiensis* genome, the *P*. *fijiensis* genome was used as a reference in the analysis. SyMap first performed an alignment of the genomes using MUMmer [93] and identify the anchor hits clusters by clustering the MUMmer hits into gene or putative gene regions. The clustered anchor regions were filtered by a reciprocal-best filtering algorithm. Synteny blocks were then identified by searching collinear sequences of anchors in the compared genomes.

### Calculation of pairwise synonymous and non-synonymous substitution rates

Pairwise synonymous and non-synonymous substitution rates (*dN* and *dS*) were calculated for the gene families with one-to-one orthology relation in the proteomes of the three species. The sequences of each family were aligned using PRANK [87] based on protein sequences and back-translated into codon alignment. The alignments were trimmed by Gblocks [88] with stringent criteria that trimmed small alignment blocks, gaps from the alignments. The Codeml program of PAML [45] was used to calculate pairwise *dN* and *dS* (mode = -2), taking the transition and transversion bias and codon usage bias into consideration. Fisher’s Exact test (FET) was used to assess the significance level of selection.

## Supporting Information

S1 Fig K-mer analysis in *Pseudocercospora musae* and *Pseudocercospora eumusae*. The K-mer (17-mer) distributions of the Illumina sequencing reads of (A) *P*. *musae* (Pmus) and (B) *P*. *eumusae* (Peum) are shown. A single major peak is present in both distributions indicating a unimodal K-mer distribution.
(TIFF)

S2 Fig Size distribution of the assembled scaffolds in the *Pseudocercospora musae* (blue) and *Pseudocercospora eumusae* (red). The high number of repetitive sequences present in the genomes of *P*. *musae* and *P*. *eumusae* lead to highly fragmented genome assemblies, in which the majority of scaffolds are less than 10 kb in size.
(TIFF)

S3 Fig Syntenic relationships among the three species that constitute the Sigatoka disease complex. (A) Dot-plot of the syntenic regions between scaffolds larger than 200 kb in size in *P*. *musae* and those larger than 200 kb in size in *P*. *eumusae* (left panel). Syntenic blocks and hits are highlighted in blue, whereas genomic inversions are shown in red. Only the regions of synteny are shown in the plot. Collinearity among scaffolds larger than 200 kb in size in the two species is also depicted as circle plots (right panel). Non-inverted blocks of synteny are connected with red ribbons, whereas inverted blocks of synteny are connected with blue ribbons. (B) Dot-plot of the syntenic regions between scaffolds larger than 200 kb in size in *P*. *musae* and those in *P*. *fijiensis* (left panel). Syntenic blocks and hits are highlighted in blue, whereas genomic inversions are shown in red. Only the regions of synteny are shown in the plot. Collinearity among scaffolds of the two species is also depicted as circle plots (right panel). Non-inverted blocks of synteny are connected with red ribbons, whereas inverted blocks of synteny are connected with blue ribbons. (C) Dot-plot of the syntenic regions between scaffolds larger than 200 kb in size in *P*. *eumusae* and those in *P*. *fijiensis* (left panel). Syntenic blocks and hits are highlighted in blue, whereas genomic inversions are shown in red. Only the regions of synteny are shown in the plot. Collinearity among the scaffolds of the two species is also depicted as circle plots (right panel). Non-inverted blocks of synteny are connected with red ribbons, whereas inverted blocks of synteny are connected with blue ribbons.
(TIF)

S4 Fig Assessment of the completeness of the gene space in the *Pseudocercospora musae* (red), *Pseudocercospora eumusae* (green), and *Pseudocercospora fijiensis* (blue) genome assemblies. A set of 248 low copy number genes that are highly conserved among eukaryotic species (CEG) is generally used to assess the quality and completeness of eukaryotic genome assemblies. These genes are classified into four CEG groups (Groups 1-to-4) based on the degree of protein sequence conservation across eukaryotes, ranging from low (Group 1), to high (Group 4) as depicted in the gradient red color bar. The Y-axis represents the percent of CEG models classified as “complete” (top section) or “partial” (bottom section) models. In a genome assembly, a predicted CEG model is considered as a “complete” model when the protein alignment length against the hidden markov model (HMM) of the orthologous genes is larger than 70% of protein length; an incomplete gene model is considered as a “partial” model if the alignment score is larger than thresholds estimated by CEGMA. Overall CEG completeness ratios were slightly higher for *P*. *eumusae* as compared to *P*. *musae* and *P*. *fijiensis* but nonetheless ratios for all three species were within the completeness ratios reported previously for other fungal genome sequencing projects.
(TIFF)

S5 Fig Estimated divergence times of *Pseudocercospora musae*, *Pseudocercospora eumusae*, *Pseudocercospora fijiensis* and 16 other representative Dothideomycetous fungi. The blue horizontal bars indicate the maximum (left end) and minimum (right end) ages of a specific node. A time scale is shown at the bottom. In the phylogenetic tree, the species fall into three major orders, i.e. Capnodiales (red), Hysteriales (blue), and Pleosporales (green), whereas *Aspergillus nidulans* (class of Eurotiomycetes) was used as an outgroup species for rooting the tree. The divergence time of the Pleosporales, Hysteriales, Capnodiales, and the three Sigatoka disease complex species are denoted next to their corresponding nodes.
(TIFF)

S6 Fig Distribution of the KOG annotation profiles of *Pseudocercospora musae*, *Pseudocercospora eumusae*, and *Pseudocercospora fijiensis*. The number of genes from each of the three species that are assigned to the individual functional subcategories of KOG is shown. KOG includes four major categories, i.e. (i) cellular processes and signaling (blue), (ii) information storage and processing (green), (iii) metabolism (orange), and (iv) poorly characterized genes (purple) that can be further classified into 25 subcategories (denoted by letter codes). The width of each pie slice is proportional to the number of genes assigned to the functional subcategory of KOG that it represents, whereas the overall ratio of the KOG term numbers assigned to each category is denoted in the rim of the pie chart. Classification of KOG: *Cellular processes and signaling*: Cell cycle control, cell division, chromosome partitioning (D); Cell motility (N); Cell wall/membrane/envelope biogenesis (M); Cytoskeleton (Z); Defense mechanisms (V); Extracellular structures (W); Intracellular trafficking, secretion, and vesicular transport (U); Nuclear structure (Y); Posttranslational modification, protein turnover, chaperones (O); Signal transduction mechanisms (T). *Information storage and processing*: Chromatin structure and dynamics (B); Replication, recombination and repair (L); RNA processing and modification (A); Transcription (K); Translation, ribosomal structure and biogenesis (J). *Metabolism*: Amino acid transport and metabolism (E); Carbohydrate transport and metabolism (G); Coenzyme transport and metabolism (H); Energy production and conversion (C); Inorganic ion transport and metabolism (P); Lipid transport and metabolism (I); Nucleotide transport and metabolism (F); Secondary metabolites biosynthesis, transport and catabolism (Q). *Poorly characterized*: Function unknown (S); General function prediction only (R).
(TIFF)

S7 Fig Distribution of KOG annotation profiles in species-specific and shared gene families among *Pseudocercospora musae*, *Pseudocercospora eumusae*, and *Pseudocercospora fijiensis*. The total number of gene families assigned to each of the four main functional categories of KOG (Cellular processes and signaling: blue; Information storage and processing: red; Metabolism: green; and Poorly characterized: purple) is enumerated for (A) the ones shared by *Pseudocercospora musae*, *Pseudocercospora eumusae*, and *Pseudocercospora fijiensis* (i.e. core gene families, shaded in green), (B) the ones shared by paired species only (shaded in orange), and (C) the ones present in only one species (species-specific genes, shaded in blue). These numbers are indicated in the different sections of each stacked column. The first number in the X-axis label of each comparison refers to the total number of gene families with KOG assigned, whereas the second number refers to the total number of gene families in each comparison compartment.
(TIF)

S8 Fig Distribution of KOG annotation profiles in shared and species-specific gene families that are lineage-specific to *Pseudocercospora musae*, *Pseudocercospora eumusae*, and *Pseudocercospora fijiensis*. The number of lineage-specific gene families assigned to each functional category of KOG (Cellular processes and signaling: blue; Information storage and processing: red; Metabolism: green; and Poorly characterized: purple) is enumerated for (A) the ones shared by all the three species (i.e. core gene families, shaded in green), (B) the ones shared by paired species only (shaded in orange), and (C) the ones present in only one of the three species (species-specific genes, shaded in blue). These numbers are indicated in the different sections of each stacked column. The first number in the X-axis label of each comparison refers to the total number of lineage-specific gene families with KOG assigned, whereas the second number refers to the total number of lineage-specific gene families in each comparison compartment.
(TIF)

S9 Fig The KOG distribution profile of equally-sized gene families in pairwise species comparisons among *Pseudocercospora musae*, *Pseudocercospora eumusae*, and *Pseudocercospora fijiensis*. The number of gene families sharing exactly the same copy number between pairwise species comparisons is enumerated and assigned to each specific functional category and sub category of KOG (Cellular processes and signaling: blue; Information storage and processing: red; Metabolism: green; and Poorly characterized: purple). The subcategories of KOG are denoted by the letter codes indicated in S6 Fig. In all comparisons, the number of gene families with the same copy number between *P*. *eumusae* and *P*. *fijiensis* is always higher as compared to *P*. *eumusae* and *P*. *musae*, or *P*. *musae* and *P*. *fijiensis*, suggesting that these two species share a more similar pattern of expansions and contractions in shared gene families.
(TIFF)

S10 Fig Hierarchical clustering of *Pseudocercospora musae*, *Pseudocercospora eumusae*, *Pseudocercospora fijiensis*, and six other representative species of Capnodiales, based on copy number changes in KOG gene families related to metabolism. (A) Hierarchical clustering of the species based on copy number changes in all the metabolic gene families that were identified in the nine species based on KOG annotations. (B) Hierarchical clustering of the species after removal of the metabolic gene families that show copy number variation only in *P*. *musae*.
(TIF)

S11 Fig Distribution of the Gene Ontology (GO) annotation profiles of *Pseudocercospora musae*, *Pseudocercospora eumusae*, and *Pseudocercospora fijiensis*. (A) Histogram of the abundance of the different functional categories of GO in the genome of the three species. GO categories are grouped by cellular component, molecular function, or biological process. (B) Hierarchical clustering of the species based on copy number changes in the different functional categories of GO. (C) Functional categories of GO that support the clustering of *P*. *eumusae* together with *P*. *fijiensis* as inferred using a random forest approach.
(TIF)

S12 Fig Mapping on a directed acyclic graph (DAG) of the functional categories of Gene Ontology (GO) terms that support the clustering of *Pseudocercospora eumusae* together with *Pseudocercospora fijiensis*. The graph illustrates, in the form of parent-to-child relationships, the connections among the different GO categories. Categories that support the clustering of the two species were inferred using a random forest approach and are highlighted with different colors. In contrast, categories that do not significantly contribute to the clustering of *P*. *eumusae* together with *P*. *fijiensis* are shown in white.
(PNG)

S13 Fig Comparison of the carbohydrate-active enzymes (CAZymes) repertoires in *Pseudocercospora musae*, *Pseudocercospora eumusae*, *Pseudocercospora fijiensis* and 16 other representative Dothideomycetes with different nutritional lifestyles. The selected 16 representative Dothideomycete species that are included in the comparison fall into three major orders: Capnodiales (red), Hysteriales (blue), and Pleosporales (green). The nutritional lifestyle of each species is indicated by a colored dot above each column: biotrophs (blue), hemi-biotrophs (green), necrotrophs (yellow), saprophytes (red). The height of each segment in the stacked bars represents the predicted number of CAzymes assigned to each of the major superfamilies of CAZymes, i.e. Glycoside Hydrolases (GHs), Glycosyl Transferases (GTs), Polysaccharide Lyases (PLs), Carbohydrate Esterases (CEs), Auxiliary Activities (AAs), and Carbohydrate-Binding Modules (CBMs). The following abbreviations are used for each species: Psemu: *Pseudocercospora musae*, Pseeu: *Pseudocercospora eumusae*, Psefi: *Pseudocercospora fijiensis*, Dotse: *Dothistroma septosporum*, Zymgr: *Zymoseptoria tritici*, Sphmu: *Sphaerulina musiva*, Sphpo: *Sphaerulina populicola*, Pleli: *Plenodomus lingam*, Exstu: *Exserohilum turcicum*, Fulfu: *Fulvia fulva*, Altbr: *Alternaria brassicicola*, Bipma: *Bipolaris maydis C4*, Bipso: *Bipolaris sorokiniana*, Pyrtt: *Pyrenophora teres f*. *teres*, Pyrtr: *Pyrenophora tritici-repentis*, Parno: *Parastagonospora nodorum*, Bauco: *Baudoinia compniacensis*, Hyspu: *Hysterium pulicare*, Rhyru: *Rhytidhysteron rufulum*.
(TIFF)

S14 Fig Hierarchical clustering of *Pseudocercospora musae*, *Pseudocercospora eumusae*, *Pseudocercospora fijiensis*, and 16 other representative Dothideomycetes with different nutritional lifestyles, based on their full profile of carbohydrate-active enzymes (CAZymes). Hierarchical clustering (top tree) was performed according to the number of enzymes from each species assigned to the individual CAZyme families of the six major superfamilies (i.e. Glycoside Hydrolases (GHs), Glycosyl Transferases (GTs), Polysaccharide Lyases (PLs), Carbohydrate Esterases (CEs), Auxiliary Activities (AAs), and Carbohydrate-Binding Modules (CBMs)), using the Manhattan distance measure and complete clustering algorithm. Bootstrap values are indicated next to nodes in the clustering tree only if the value is higher than 50%. Abundance of enzymes within a family are shaded from grey (min: 0) to red (max: 55). The clustering tree indicates that *P*. *eumusae* is clustered first with *P*. *fijiensis*, followed by *P*. *musae* with a strong bootstrap value (100).
(TIFF)

S15 Fig Comparison of repertoires for plant cell wall degrading enzymes (PCWDEs) and/or fungal cell wall degrading enzymes, between *Pseudocercospora musae*, *Pseudocercospora eumusae*, *Pseudocercospora fijiensis*, and 16 other representative Dothideomycetes with different nutritional lifestyles. The selected 16 representative Dothideomycete species that are included in the comparison fall into three major orders: Capnodiales (red), Hysteriales (blue), and Pleosporales (green). The nutritional lifestyle of each species is indicated by a colored dot above each column: biotrophs (blue), hemi-biotrophs (green), necrotrophs (yellow), saprophytes (red). The height of each segment in the stacked bars represents the predicted number of carbohydrate-active enzymes (CAZymes) that are involved in the degradation of cellulose (light blue), hemicellulose (orange), hemicellulose-pectin (grey), pectin (yellow), fungal cell walls (blue), and fungal or plant cell wall (light green). The following abbreviations are used for each species: Psemu: *Pseudocercospora musae*, Pseeu: *Pseudocercospora eumusae*, Psefi: *Pseudocercospora fijiensis*, Dotse: *Dothistroma septosporum*, Zymgr: *Zymoseptoria tritici*, Sphmu: *Sphaerulina musiva*, Sphpo: *Sphaerulina populicola*, Pleli: *Plenodomus lingam*, Exstu: *Exserohilum turcicum*, Fulfu: *Fulvia fulva*, Altbr: *Alternaria brassicicola*, Bipma: *Bipolaris maydis C4*, Bipso: *Bipolaris sorokiniana*, Pyrtt: *Pyrenophora teres f*. *teres*, Pyrtr: *Pyrenophora tritici-repentis*, Parno: *Parastagonospora nodorum*, Bauco: *Baudoinia compniacensis*, Hyspu: *Hysterium pulicare*, Rhyru: *Rhytidhysteron rufulum*.
(TIFF)

S16 Fig Comparison of the capacity of *Pseudocercospora musae*, *Pseudocercospora eumusae*, and *Pseudocercospora fijiensis* to degrade cellulose, hemicellulose, hemicellulose-pectin and pectin according to their arsenal of plant cell wall degrading enzymes (PCWDEs). Families of PCWDEs are grouped based on their major targeted substrates, including cellulose (shaded in blue), hemicellulose (shaded in orange), hemicellulose-pectin (shaded in grey), and pectin (shaded in yellow). Segments in the stacked bars represent *P*. *musae* (grey), *P*. *eumusae* (orange), or *P*. *fijiensis* (blue), and their height is proportional to the number of enzymes from each species assigned to the family of PCWDEs that the individual stacked bars represent. Stars indicate differences more than two.
(TIF)

S17 Fig Clustering of Type I Polyketide Synthases (PKS) and Hybrid Polyketide Synthase-Nonribosomal Peptide Synthetases (PKS-NRPSs) from *Pseudocercospora musae*, *Pseudocercospora eumusae*, and *Pseudocercospora fijiensis*, with representatives from other fungal species. The phylogenetic tree is built based on the conserved ketoacyl synthase (KS) and acyltransferase (AT) domain sequences of each modular PKS and PKS-NRPS enzyme, using maximum likelihood with 1000 replications. Bootstrap values > 50% are labeled next to corresponding nodes. Well supported clades (bootstrap values >80%) that include PKSs or PKS-NRPSs involved in the biosynthesis of known secondary metabolites (SMs) and toxins are shaded in red for the non-reducing Type I PKS, in blue for the reducing Type I PKS, and in green for the hybrid PKS-NRPSs. PKSs and PKS-NRPSs from the three Sigatoka disease complex species are highlighted in red. The domain architecture of each modular PKS and PKS-NRPS enzyme is plotted next to its name. The abbreviations used for each species are indicated in the legend.
(TIFF)

S18 Fig Clustering of Nonribosomal Peptide Synthetases (NRPSs) from *Pseudocercospora musae*, *Pseudocercospora eumusae*, and *Pseudocercospora fijiensis*, with representatives from other fungal species. The phylogenetic tree is built based on the adenylation (A) domain sequences of each modular NRPS enzyme, using maximum likelihood with 1000 replications. Bootstrap values > 50% are labeled next to corresponding nodes. Well supported clades (bootstrap values >80%) that include NRPSs involved in the biosynthesis of known secondary metabolites (SMs) and toxins are shaded in different colors. NRPSs from the three Sigatoka disease complex species are highlighted in red. The domain architecture of each modular NRPS enzyme is plotted next to its name, whereas the outer ring represents the three major subfamilies to which NRPSs from *P*. *musae*, *P*. *eumusae*, and *P*. *fijiensis* can be classified, i.e. siderophore synthetases (SID), Euascomycete clade synthetases (EAS), and cyclosporin synthetases (CYCLO). The following abbreviations are used for each species: Psemu: *Pseudocercospora musae*, Pseeu: *Pseudocercospora eumusae*, Psefi: *Pseudocercospora fijiensis*, Altal: *Alternaria alternata*, Aspfu: *Aspergillus fumigatus*, Fulfu: *Fulvia fulva*, Clapu: *Claviceps purpurea*, Bipze: *Bipolaris zeicola*, Bipma: *Bipolaris maydis*, Epife: *Epichloe festucae*, Fuseq: *Fusarium equiseti*, Fusgra: *Fusarium graminearum*, Pyror: *Pyricularia oryzae*, Ompol: *Omphalotus olearius*, Schpo: *Schizosaccharomyces pombe*, Trivi: *Trichoderma virens*, Ustma: *Ustilago maydis*.
(TIFF)

S19 Fig Clustering of Terpene Synthases (TSs) from *Pseudocercospora musae*, *Pseudocercospora eumusae*, and *Pseudocercospora fijiensis*, with representatives from other fungal species. The phylogenetic tree is built based on an amino acid alignment of the full-length TSs, using maximum likelihood with 1000 replications. Bootstrap values > 50% are labeled next to corresponding nodes. Well supported clades (bootstrap values >80%) that include TSs involved in the biosynthesis of known secondary metabolites (SMs) and toxins are shaded in different colors. TSs from the three Sigatoka disease complex species are highlighted in red. The abbreviations used for each species are indicated in the legend.
(TIFF)

S20 Fig The workflow used for the prediction of the secreted proteins and effectors in *Pseudocercospora musae*, *Pseudocercospora eumusae*, and *Pseudocercospora fijiensis*. (TIFF)

S21 Fig Shared and species-specific effectors in *Pseudocercospora musae*, *Pseudocercospora eumusae*, and *Pseudocercospora fijiensis*. (A) Black numbers in the venn diagram show the total number of species-specific and shared effectors between and among the three species. Blue numbers in parentheses the species-specific sectors correspond to orphans (i.e effectors that do not have homologs in other fungi), whereas red numbers in parentheses in the intersections of shared effectors correspond to lineage-specific ones (i.e. effectors that are present only in at least two of the Sigatoka disease complex species). Reciprocal BlastP best hit (e-value: 1e-5) analysis implemented in OrthoMCL was used to retrieved the set of effectors shared by the three species, while BlastP (e-value: 1e-5, alignment coverage > 50%) against the NCBI nr database and the JGI fungal genome database was used to identify putative homologs in other fungal species and beyond. (B-D) The analysis is expanded to include a broader search for homologs of each species effectors against the entire proteome of the other two species. As before, black numbers correspond to comparisons among *P*. *musae*, *P*. *eumusae*, and *P*. *fijiensis*, while blue and red numbers in parentheses correspond to orphan and lineage-specific effectors, respectively.
(TIFF)

S22 Fig Amplification using PCR of 12 species-specific or core effectors from (A) *Pseudocercospora musae*, (B) *Pseudocercospora eumusae*, and (C) *Pseudocercospora fijiensis* from a set of seven field isolates of each species. PCR and subsequent sequencing analysis of the amplified products (S10 Table) confirmed that the 12 randomly selected species-specific and orphan effectors are conserved in each species and, as expected, absent in the other two species.
(TIF)

S23 Fig Box-and-whisker diagrams of the distribution of *dN*/*dS* ratios in partitions of the species’ proteomes that relate to secreted proteins and effectors. Pairwise *dN*/*dS* ratios (ω) (dots in the plot) were calculated for the gene families with one-to-one orthology in the three Sigatoka disease complex species. In order to examine whether different parts of the species proteomes are evolving under different rates, the gene families were partitioned into five groups, i.e those encoding for i) effectors, ii) non-effector secreted proteins, iii) secreted proteins, iv) non-secreted proteins, and iv) the total number of proteins. The mean, median, and total number of comparisons is shown at the top of each group. The *dN*/*dS* ratios were classified into three compartments along the y-axis (ω ≤ 0.5, 0.5 < ω ≤1, and ω > 1), in which the number of pairwise comparisons is denoted. In each group, an additional number (ω >1(s)) is denoted for the number of comparisons that have a *dN*/*dS* ratios > 1 and *P*-value < 0.05 based on Fisher’s exact tests.
(TIFF)

S24 Fig Box-and-whisker diagrams of the distribution of *dN*/*dS* ratios in partitions of the species’ proteomes that relate to the different categories of KOG. Pairwise *dN*/*dS* ratios (ω) (dots in the plot) were calculated for the gene families with one-to-one orthology in the three Sigatoka disease complex species. In order to examine whether different parts of the species proteomes are evolving under different rates, the gene families were partitioned into six groups based on the KOG functional assignment of their encoded proteins, i.e. i) cellular process and signaling, ii) information storage and processing, iii) metabolism, iv) poorly characterized, v) proteins with no KOG assignment, and vi) the total number of proteins. The mean, median, and total number of comparisons is shown at the top of each group. The *dN*/*dS* ratios were classified into three compartments along the y-axis (ω ≤ 0.5, 0.5 < ω ≤1, and ω > 1), in which the number of pairwise comparisons is denoted. In each group, an additional number (ω >1(s)) is denoted for the number of comparisons that have a *dN*/*dS* ratios > 1 and *P*-value < 0.05 based on Fisher’s exact tests.
(TIFF)

S25 Fig Box-and-whisker diagrams of the distribution of *dN*/*dS* ratios in partitions of the species’ proteomes that relate to carbohydrate-active enzymes (CAZymes) with a role in the degradation of plant and/or fungal cell walls. Pairwise *dN*/*dS* ratios (ω) (dots in the plot) were calculated for the gene families with one-to-one orthology in the three Sigatoka disease complex species. In order to examine whether different parts of the species CAZyomes are evolving under different rates, the gene families were partitioned into six groups based on whether their encoded enzymes are involved in the degradation of i) plant cell wall cellulose (PCWD-Cellulose), ii) plant cell wall hemicellulose (PCWD-hemicellulose), iii) plant cell wall hemicellulose-pectin (PCWD-HP), iv) plant cell wall pectin (PCWD-pectin), v) fungal cell walls (FCWD), and vi) fungal and/or plant cell walls (FPCWD). The mean, median, and total number of comparisons is shown at the top of each group. The *dN*/*dS* ratios were classified into three compartments along the y-axis (ω ≤ 0.5, 0.5 < ω ≤1, and ω > 1), in which the number of pairwise comparisons is denoted. In each group, an additional number (ω >1(s)) is denoted for the number of comparisons that have a *dN*/*dS* ratios > 1 and *P*-value < 0.05 based on Fisher’s exact tests.
(TIFF)

S1 Table Genome assembly and annotation statistics. (DOCX)

S2 Table Annotation of transposable elements and other repeat sequences in *Pseudocercospora musae*, *Pseudocercospora eumusae*, and *Pseudocercospora fijiensis*. (DOCX)

S3 Table Summary statistics of repeat induced point mutation (RIP) in *Pseudocercospora musae*, *Pseudocercospora eumusae*, and *Pseudocercospora fijiensis*. (DOCX)

S4 Table Repertoires of carbohydrate-active enzymes (CAZymes) in *Pseudocercospora musae*, *Pseudocercospora eumusae*, *Pseudocercospora fijiensis*, and 16 other representative Dothideomycetes with different nutritional lifestyles. (XLSX)

S5 Table Summary of the total number of carbohydrate-active enzymes (CAZymes) involved in the degradation of plant and fungal cell walls in *Pseudocercospora musae*, *Pseudocercospora eumusae*, *Pseudocercospora fijiensis*, and 16 other representative Dothideomycetes with different nutritional lifestyles. (XLSX)

S6 Table Comparison of the repertoires of carbohydrate-active enzymes (CAZymes) involved in the degradation of plant cell walls in *Pseudocercospora musae*, *Pseudocercospora eumusae*, *Pseudocercospora fijiensis*, and 16 other Dothideomycetes with different nutritional lifestyles. (XLSX)

S7 Table Annotation of genes in *Pseudocercospora musae*, *Pseudocercospora eumusae*, and *Pseudocercospora fijiensis* encoding the core enzyme types that catalyze the first committed step in the biosynthesis of the major classes of secondary metabolites, i.e the polyketide synthases (PKSs), the non-ribosomal peptide synthases (NRPSs), and the terpene synthases (TSs). (XLSX)

S8 Table Total number of predicted secreted proteins in *Pseudocercospora musae*, *Pseudocercospora eumusae*, *Pseudocercospora fijiensis*, and 16 other Dothideomycetes with different nutritional lifestyles. (XLSX)

S9 Table Annotation of candidate effectors in *Pseudocercospora musae*, *Pseudocercospora eumusae*, and *Pseudocercospora fijiensis*. (XLSX)

S10 Table Sequence alignments of the 12 species-specific or core effectors from *Pseudocercospora musae*, *Pseudocercospora eumusae*, and *Pseudocercospora fijiensis* that were amplified from a set of seven field isolates of each species. (XLSX)

S11 Table Annotation of the gene families with high *dN*/*dS* ratio. (XLSX)

S12 Table Sequences of the primers that were used to amplify a set of 12 species-specific or core effectors from field isolates of *Pseudocercospora musae*, *Pseudocercospora eumusae*, and *Pseudocercospora fijiensis*. (DOCX)

S1 Text Supporting Results. Supporting Results includes additional information and discussion on the different analyses that have been performed. The following theme paragraphs (P) are included: **P1:** Analysis of syntenic relationships reveals strong locally conserved gene order and content, interrupted by repeat elements. **P2:** The three Sigatoka species display mark differences in their repertoire of transposable elements (TEs). **P3:** The efficacy and specificity of RIP in transposable elements and beyond differs among the three species. **P4:** Functional annotation and characterization of the species’ gene complement indicate abundant species- and lineage-specific adaptations. **P5:** Analysis of copy-number variations (CNV) reveals parallel patterns of gene family expansions and contractions between *P*. *fijiensis* and *P*. *eumusae*. **P6:** CAZy annotations and characterization of plant cell wall degrading enzymes (PCWDEs) suggest small differences among the three species but also more similar profiles for *P*. *eumusae* and *P*. *fijiensis* as compared to *P*. *musae*.**P7:** Annotation of the core enzymes involved in the biosynthesis of secondary metabolites (SMs) reveals that the three Sigatoka species potentially produce a diverse but only partially overlapping array of SMs. **P8:** Effector characterization indicates that the three pathogens exhibit overlapping but still very dissimilar repertoires of candidate effectors. **P9:** Supporting References.
(PDF)
