# Supplementary figures and images for: Comparative Genomics of the Sigatoka Disease Complex on Banana Suggests a Link between Parallel Evolutionary Changes in Pseudocercospora fijiensis and Pseudocercospora eumusae and Increased Virulence on the Banana Host
Source: PLoS Genet. 2016 Aug 11;12(8):e1005904. doi: 10.1371/journal.pgen.1005904 (PMC4981473; doi:10.1371/journal.pgen.1005904)

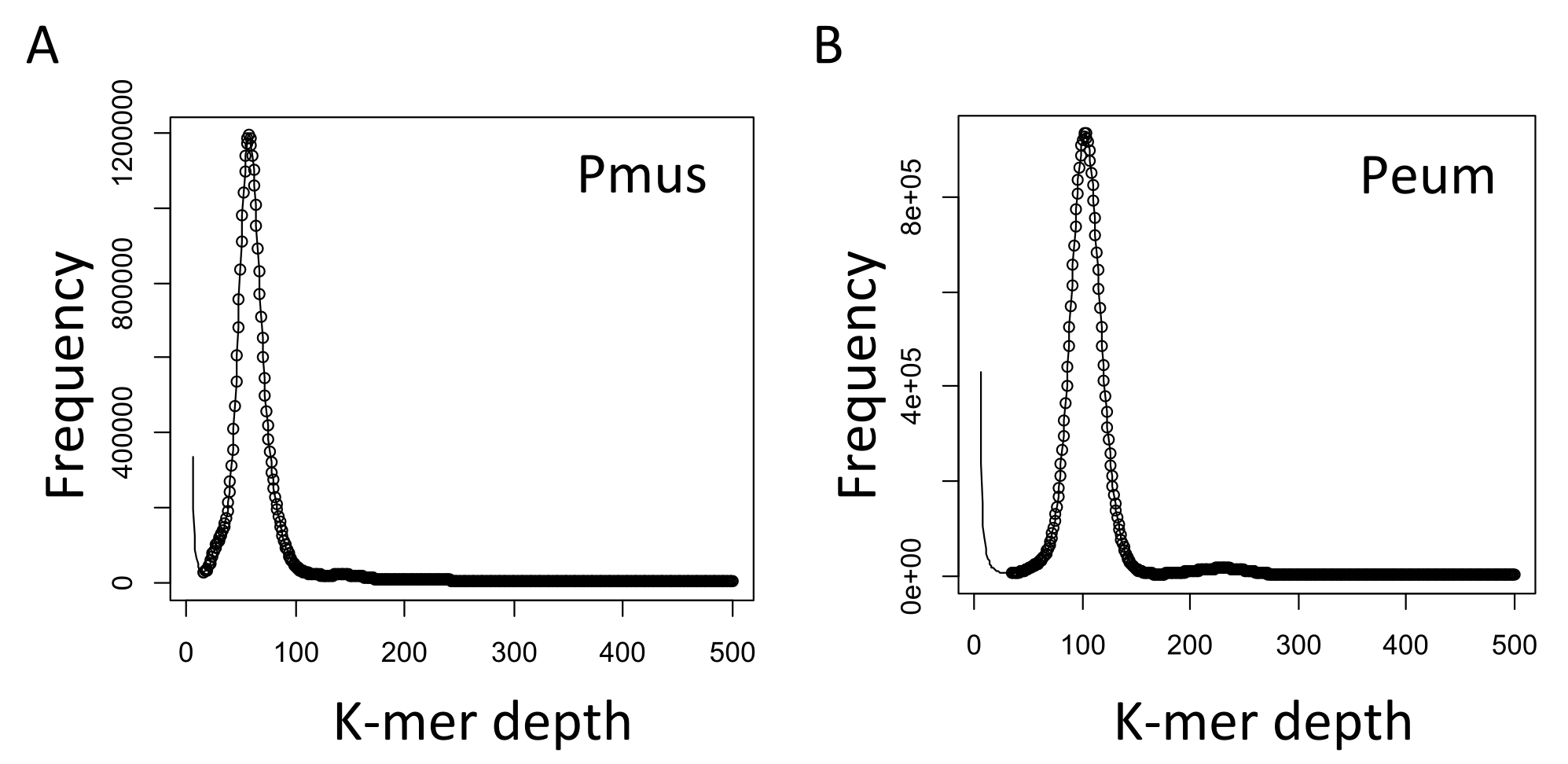

Supplement: S1 Fig — The K-mer (17-mer) distributions of the Illumina sequencing reads of (A) P. musae (Pmus) and (B) P. eumusae (Peum) are shown. A single major peak is present in both distributions indicating a unimodal K-mer distribution. (TIFF) [file pgen.1005904.s001.tiff]

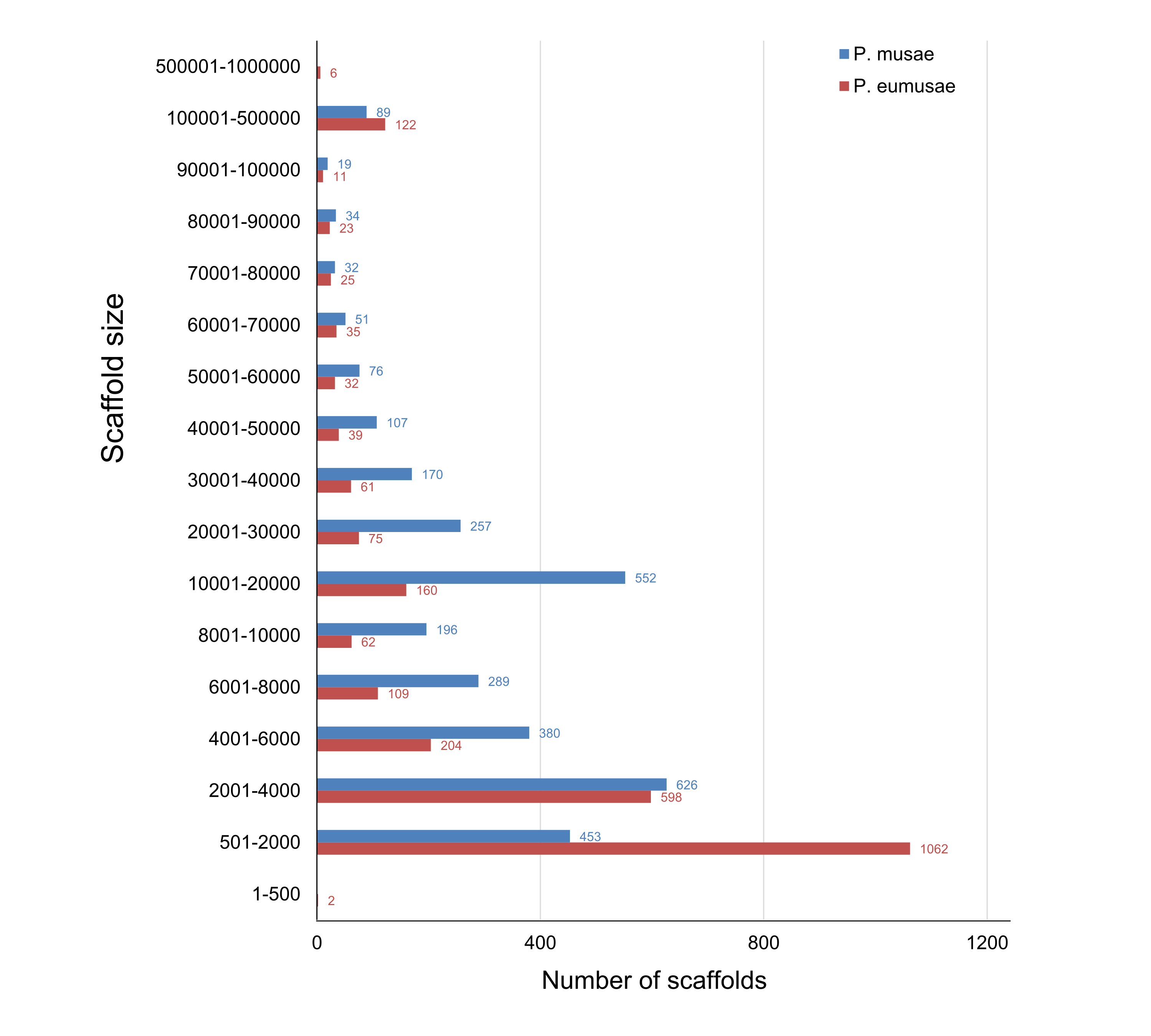

Supplement: S2 Fig — The high number of repetitive sequences present in the genomes of P. musae and P. eumusae lead to highly fragmented genome assemblies, in which the majority of scaffolds are less than 10 kb in size. (TIFF) [file pgen.1005904.s002.tiff]

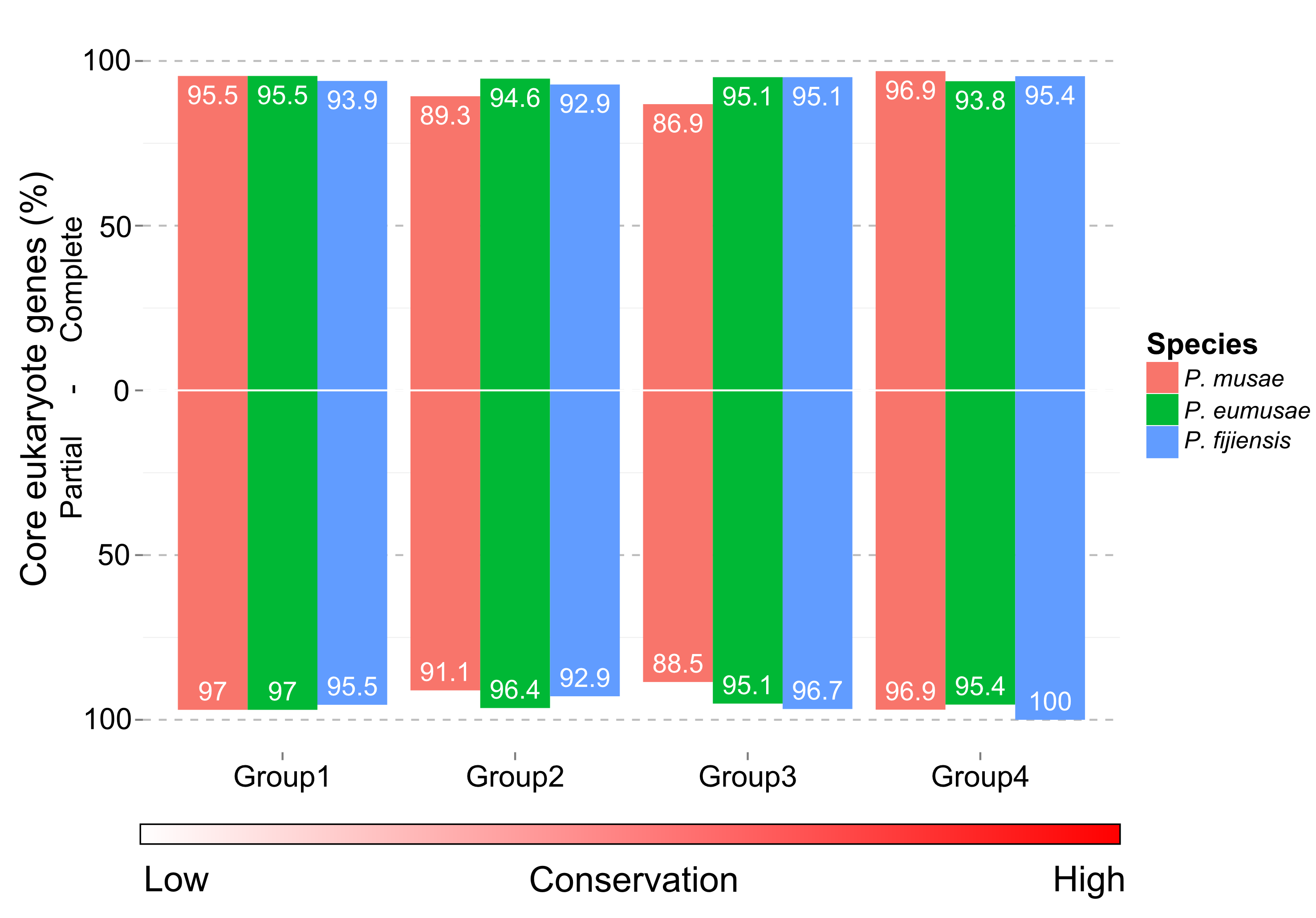

Supplement: S4 Fig — A set of 248 low copy number genes that are highly conserved among eukaryotic species (CEG) is generally used to assess the quality and completeness of eukaryotic genome assemblies. These genes are classified into four CEG groups (Groups 1-to-4) based on the degree of protein sequence conservation across eukaryotes, ranging from low (Group 1), to high (Group 4) as depicted in the gradient red color bar. The Y-axis represents the percent of CEG models classified as “complete” (top section) or “partial” (bottom section) models. In a genome assembly, a predicted CEG model is considered as a “complete” model when the protein alignment length against the hidden markov model (HMM) of the orthologous genes is larger than 70% of protein length; an incomplete gene model is considered as a “partial” model if the alignment score is larger than thresholds estimated by CEGMA. Overall CEG completeness ratios were slightly higher for P. eumusae as compared to P. musae and P. fijiensis but nonetheless ratios for all three species were within the completeness ratios reported previously for other fungal genome sequencing projects. (TIFF) [file pgen.1005904.s004.tiff]

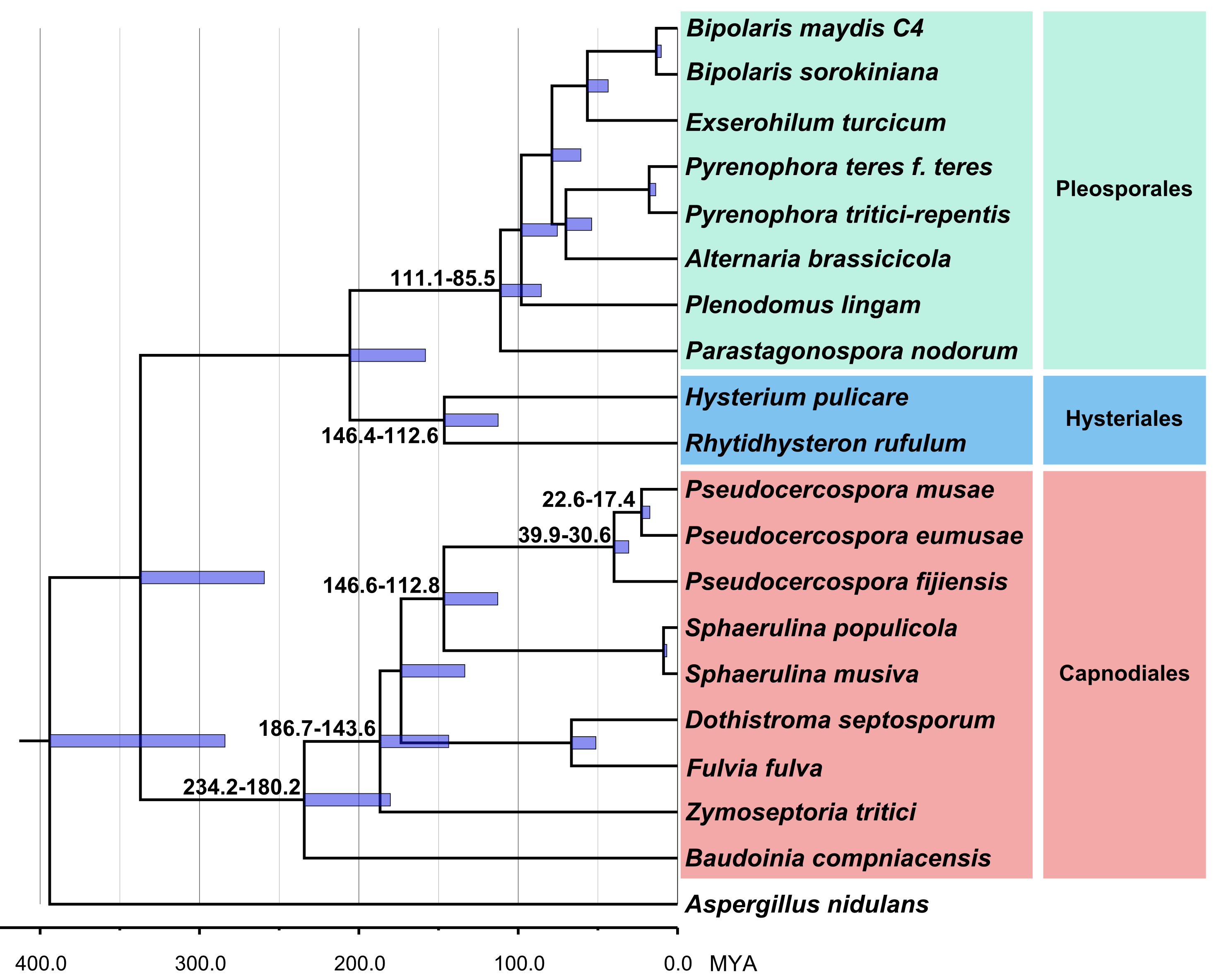

Supplement: S5 Fig — The blue horizontal bars indicate the maximum (left end) and minimum (right end) ages of a specific node. A time scale is shown at the bottom. In the phylogenetic tree, the species fall into three major orders, i.e. Capnodiales (red), Hysteriales (blue), and Pleosporales (green), whereas Aspergillus nidulans (class of Eurotiomycetes) was used as an outgroup species for rooting the tree. The divergence time of the Pleosporales, Hysteriales, Capnodiales, and the three Sigatoka disease complex species are denoted next to their corresponding nodes. (TIFF) [file pgen.1005904.s005.tiff]

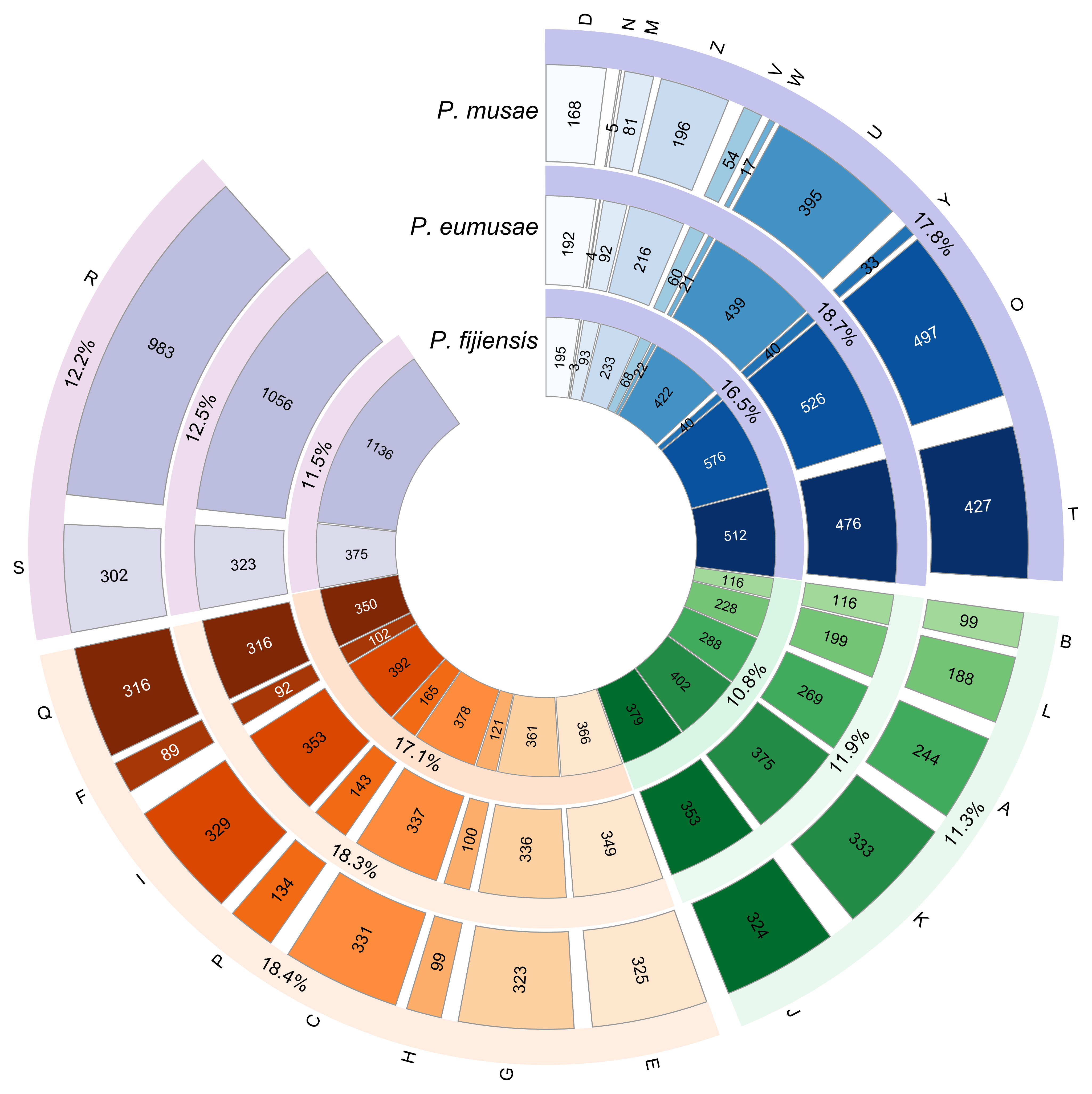

Supplement: S6 Fig — The number of genes from each of the three species that are assigned to the individual functional subcategories of KOG is shown. KOG includes four major categories, i.e. (i) cellular processes and signaling (blue), (ii) information storage and processing (green), (iii) metabolism (orange), and (iv) poorly characterized genes (purple) that can be further classified into 25 subcategories (denoted by letter codes). The width of each pie slice is proportional to the number of genes assigned to the functional subcategory of KOG that it represents, whereas the overall ratio of the KOG term numbers assigned to each category is denoted in the rim of the pie chart. Classification of KOG: Cellular processes and signaling: Cell cycle control, cell division, chromosome partitioning (D); Cell motility (N); Cell wall/membrane/envelope biogenesis (M); Cytoskeleton (Z); Defense mechanisms (V); Extracellular structures (W); Intracellular trafficking, secretion, and vesicular transport (U); Nuclear structure (Y); Posttranslational modification, protein turnover, chaperones (O); Signal transduction mechanisms (T). Information storage and processing: Chromatin structure and dynamics (B); Replication, recombination and repair (L); RNA processing and modification (A); Transcription (K); Translation, ribosomal structure and biogenesis (J). Metabolism: Amino acid transport and metabolism (E); Carbohydrate transport and metabolism (G); Coenzyme transport and metabolism (H); Energy production and conversion (C); Inorganic ion transport and metabolism (P); Lipid transport and metabolism (I); Nucleotide transport and metabolism (F); Secondary metabolites biosynthesis, transport and catabolism (Q). Poorly characterized: Function unknown (S); General function prediction only (R). (TIFF) [file pgen.1005904.s006.tiff]

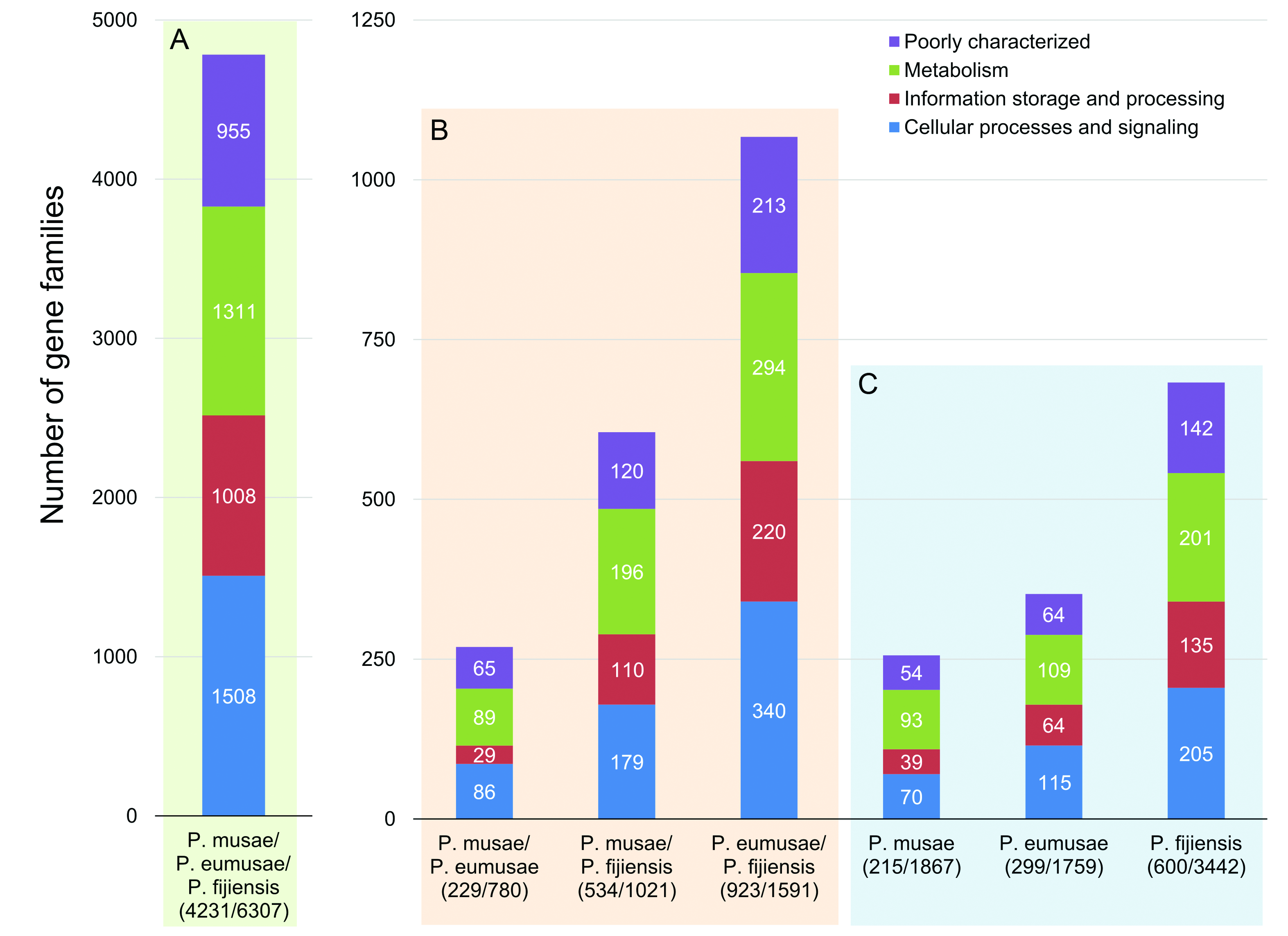

Supplement: S7 Fig — The total number of gene families assigned to each of the four main functional categories of KOG (Cellular processes and signaling: blue; Information storage and processing: red; Metabolism: green; and Poorly characterized: purple) is enumerated for (A) the ones shared by Pseudocercospora musae, Pseudocercospora eumusae, and Pseudocercospora fijiensis (i.e. core gene families, shaded in green), (B) the ones shared by paired species only (shaded in orange), and (C) the ones present in only one species (species-specific genes, shaded in blue). These numbers are indicated in the different sections of each stacked column. The first number in the X-axis label of each comparison refers to the total number of gene families with KOG assigned, whereas the second number refers to the total number of gene families in each comparison compartment. (TIF) [file pgen.1005904.s007.tif]

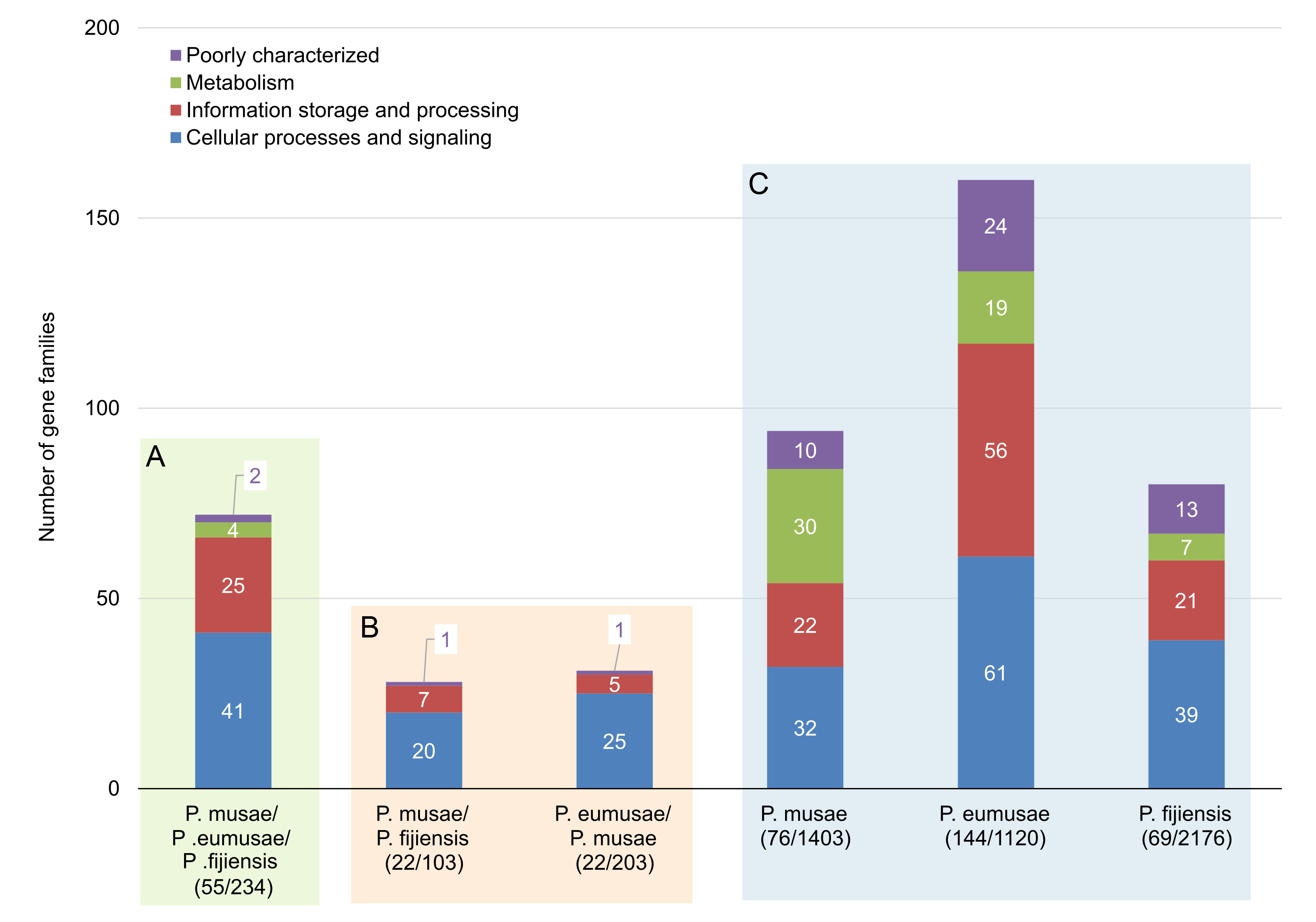

Supplement: S8 Fig — The number of lineage-specific gene families assigned to each functional category of KOG (Cellular processes and signaling: blue; Information storage and processing: red; Metabolism: green; and Poorly characterized: purple) is enumerated for (A) the ones shared by all the three species (i.e. core gene families, shaded in green), (B) the ones shared by paired species only (shaded in orange), and (C) the ones present in only one of the three species (species-specific genes, shaded in blue). These numbers are indicated in the different sections of each stacked column. The first number in the X-axis label of each comparison refers to the total number of lineage-specific gene families with KOG assigned, whereas the second number refers to the total number of lineage-specific gene families in each comparison compartment. (TIF) [file pgen.1005904.s008.tif]

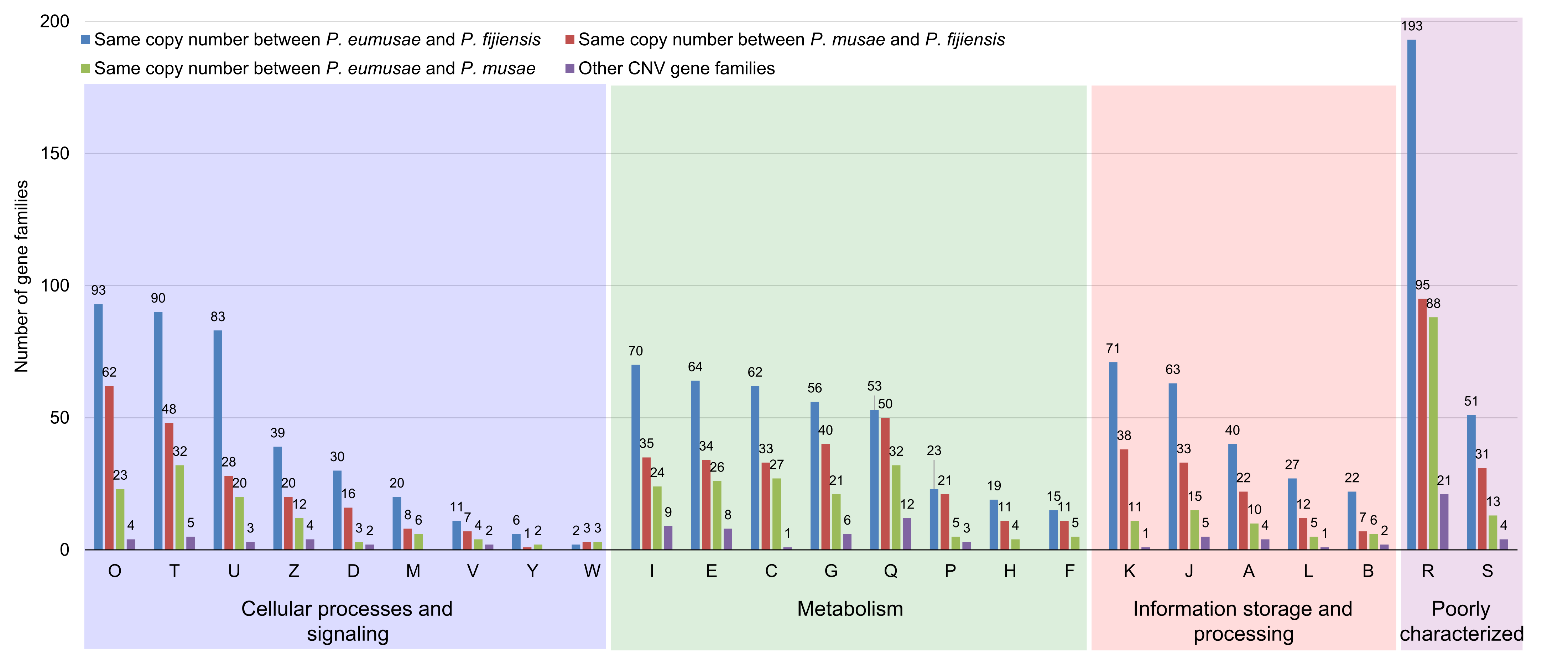

Supplement: S9 Fig — The number of gene families sharing exactly the same copy number between pairwise species comparisons is enumerated and assigned to each specific functional category and sub category of KOG (Cellular processes and signaling: blue; Information storage and processing: red; Metabolism: green; and Poorly characterized: purple). The subcategories of KOG are denoted by the letter codes indicated in S6 Fig. In all comparisons, the number of gene families with the same copy number between P. eumusae and P. fijiensis is always higher as compared to P. eumusae and P. musae, or P. musae and P. fijiensis, suggesting that these two species share a more similar pattern of expansions and contractions in shared gene families. (TIFF) [file pgen.1005904.s009.tiff]

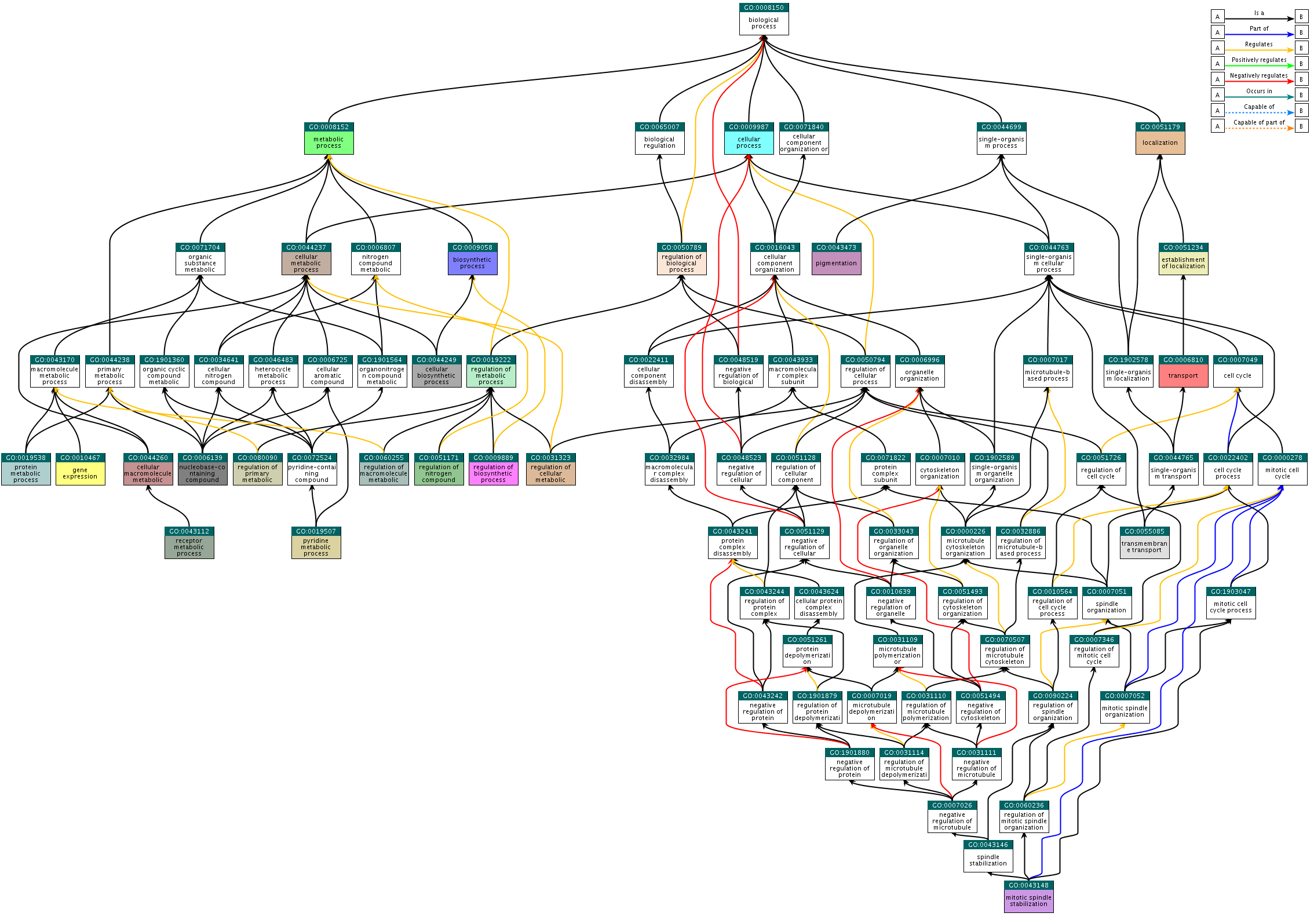

Supplement: S12 Fig — The graph illustrates, in the form of parent-to-child relationships, the connections among the different GO categories. Categories that support the clustering of the two species were inferred using a random forest approach and are highlighted with different colors. In contrast, categories that do not significantly contribute to the clustering of P. eumusae together with P. fijiensis are shown in white. (PNG) [file pgen.1005904.s012.png]

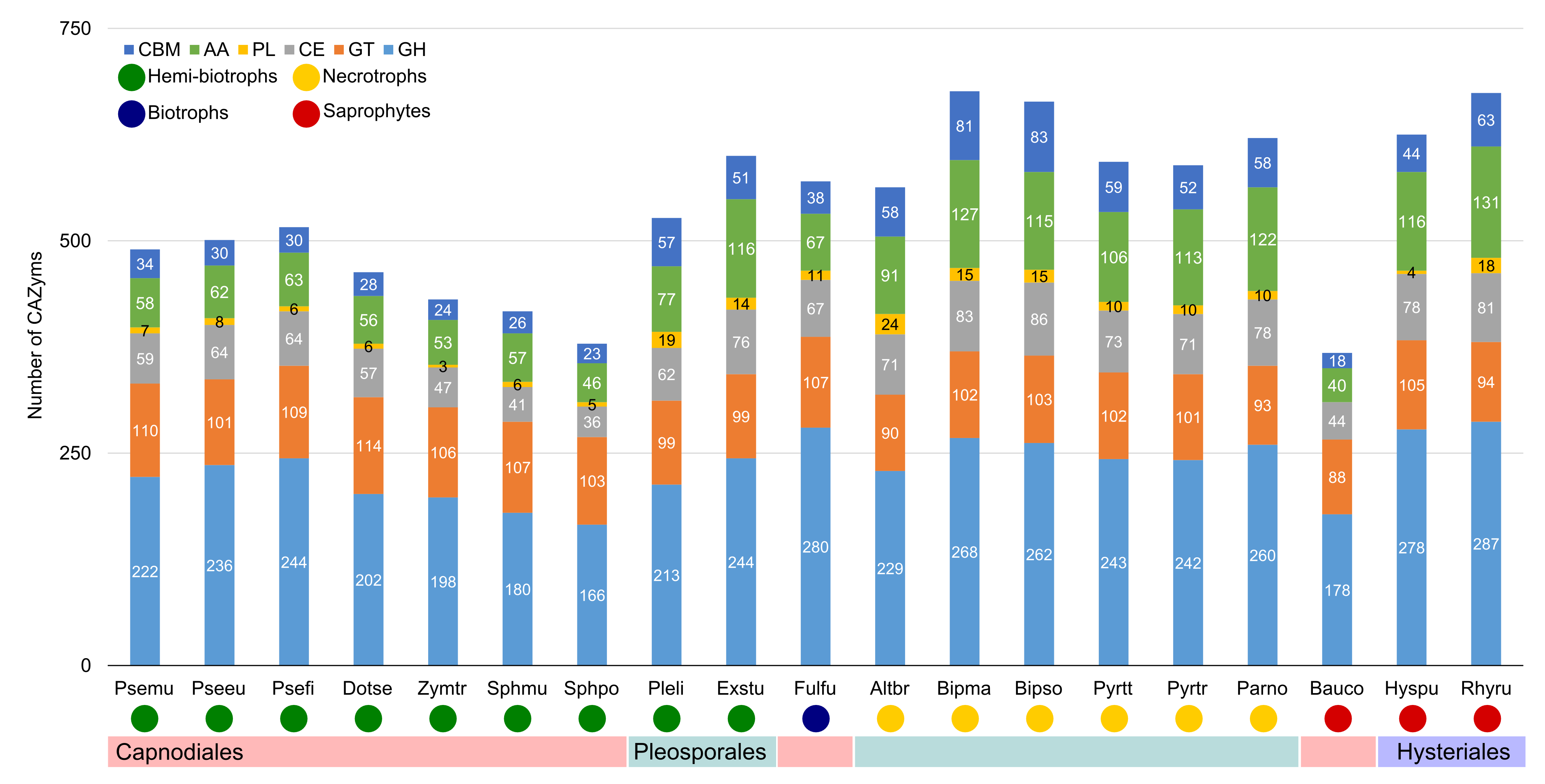

Supplement: S13 Fig — The selected 16 representative Dothideomycete species that are included in the comparison fall into three major orders: Capnodiales (red), Hysteriales (blue), and Pleosporales (green). The nutritional lifestyle of each species is indicated by a colored dot above each column: biotrophs (blue), hemi-biotrophs (green), necrotrophs (yellow), saprophytes (red). The height of each segment in the stacked bars represents the predicted number of CAzymes assigned to each of the major superfamilies of CAZymes, i.e. Glycoside Hydrolases (GHs), Glycosyl Transferases (GTs), Polysaccharide Lyases (PLs), Carbohydrate Esterases (CEs), Auxiliary Activities (AAs), and Carbohydrate-Binding Modules (CBMs). The following abbreviations are used for each species: Psemu: Pseudocercospora musae, Pseeu: Pseudocercospora eumusae, Psefi: Pseudocercospora fijiensis, Dotse: Dothistroma septosporum, Zymgr: Zymoseptoria tritici, Sphmu: Sphaerulina musiva, Sphpo: Sphaerulina populicola, Pleli: Plenodomus lingam, Exstu: Exserohilum turcicum, Fulfu: Fulvia fulva, Altbr: Alternaria brassicicola, Bipma: Bipolaris maydis C4, Bipso: Bipolaris sorokiniana, Pyrtt: Pyrenophora teres f. teres, Pyrtr: Pyrenophora tritici-repentis, Parno: Parastagonospora nodorum, Bauco: Baudoinia compniacensis, Hyspu: Hysterium pulicare, Rhyru: Rhytidhysteron rufulum. (TIFF) [file pgen.1005904.s013.tiff]

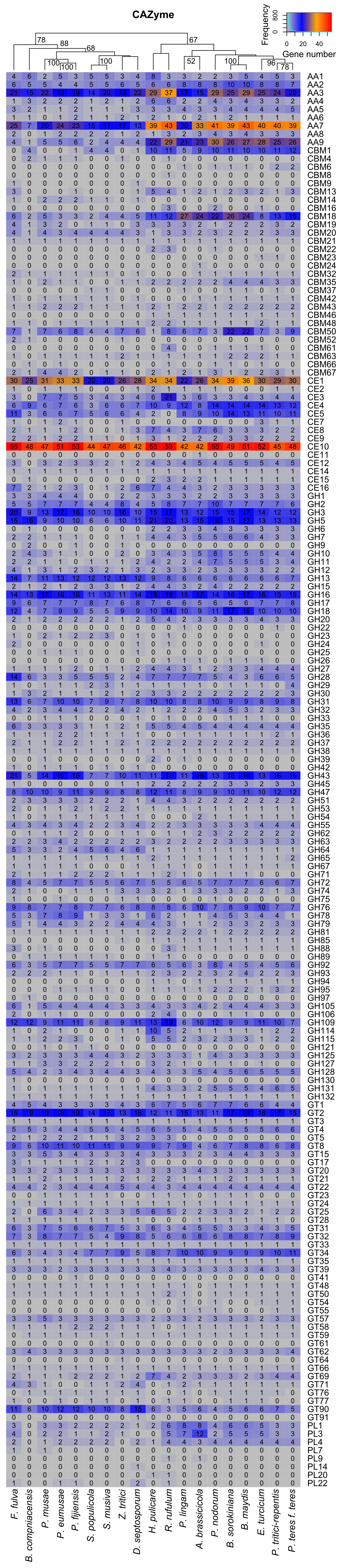

Supplement: S14 Fig — Hierarchical clustering (top tree) was performed according to the number of enzymes from each species assigned to the individual CAZyme families of the six major superfamilies (i.e. Glycoside Hydrolases (GHs), Glycosyl Transferases (GTs), Polysaccharide Lyases (PLs), Carbohydrate Esterases (CEs), Auxiliary Activities (AAs), and Carbohydrate-Binding Modules (CBMs)), using the Manhattan distance measure and complete clustering algorithm. Bootstrap values are indicated next to nodes in the clustering tree only if the value is higher than 50%. Abundance of enzymes within a family are shaded from grey (min: 0) to red (max: 55). The clustering tree indicates that P. eumusae is clustered first with P. fijiensis, followed by P. musae with a strong bootstrap value (100). (TIFF) [file pgen.1005904.s014.tiff]

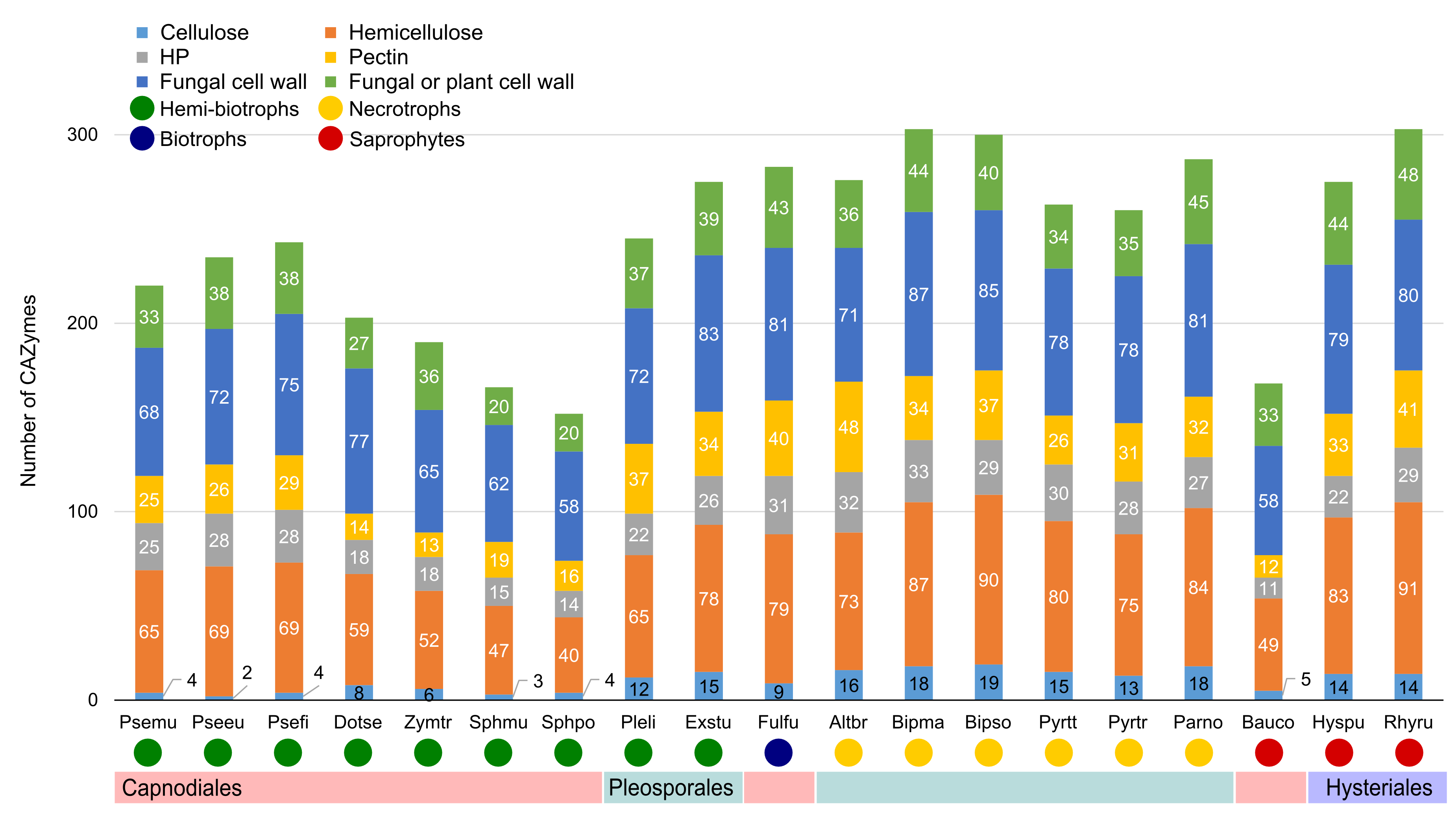

Supplement: S15 Fig — The selected 16 representative Dothideomycete species that are included in the comparison fall into three major orders: Capnodiales (red), Hysteriales (blue), and Pleosporales (green). The nutritional lifestyle of each species is indicated by a colored dot above each column: biotrophs (blue), hemi-biotrophs (green), necrotrophs (yellow), saprophytes (red). The height of each segment in the stacked bars represents the predicted number of carbohydrate-active enzymes (CAZymes) that are involved in the degradation of cellulose (light blue), hemicellulose (orange), hemicellulose-pectin (grey), pectin (yellow), fungal cell walls (blue), and fungal or plant cell wall (light green). The following abbreviations are used for each species: Psemu: Pseudocercospora musae, Pseeu: Pseudocercospora eumusae, Psefi: Pseudocercospora fijiensis, Dotse: Dothistroma septosporum, Zymgr: Zymoseptoria tritici, Sphmu: Sphaerulina musiva, Sphpo: Sphaerulina populicola, Pleli: Plenodomus lingam, Exstu: Exserohilum turcicum, Fulfu: Fulvia fulva, Altbr: Alternaria brassicicola, Bipma: Bipolaris maydis C4, Bipso: Bipolaris sorokiniana, Pyrtt: Pyrenophora teres f. teres, Pyrtr: Pyrenophora tritici-repentis, Parno: Parastagonospora nodorum, Bauco: Baudoinia compniacensis, Hyspu: Hysterium pulicare, Rhyru: Rhytidhysteron rufulum. (TIFF) [file pgen.1005904.s015.tiff]

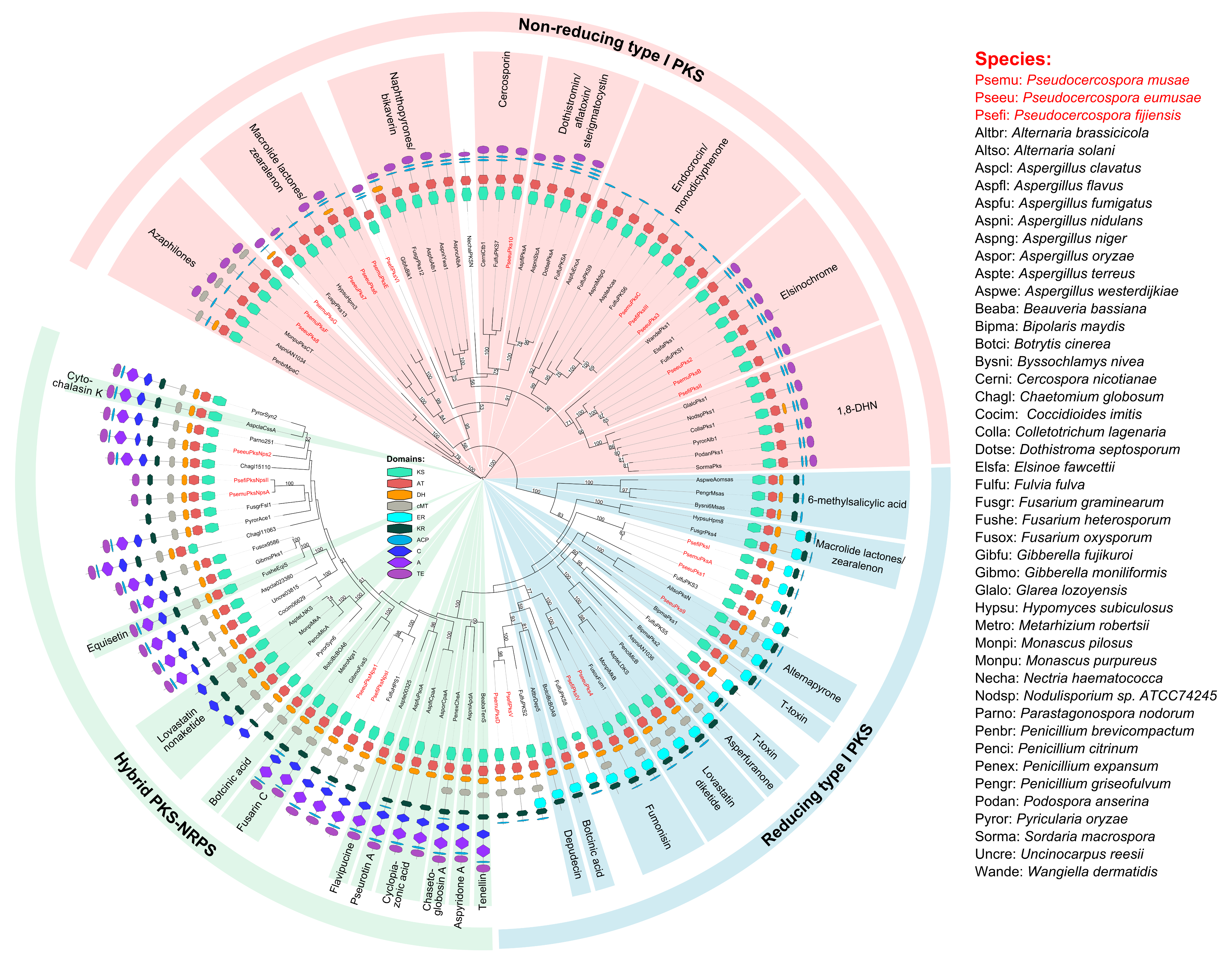

Supplement: S17 Fig — The phylogenetic tree is built based on the conserved ketoacyl synthase (KS) and acyltransferase (AT) domain sequences of each modular PKS and PKS-NRPS enzyme, using maximum likelihood with 1000 replications. Bootstrap values > 50% are labeled next to corresponding nodes. Well supported clades (bootstrap values >80%) that include PKSs or PKS-NRPSs involved in the biosynthesis of known secondary metabolites (SMs) and toxins are shaded in red for the non-reducing Type I PKS, in blue for the reducing Type I PKS, and in green for the hybrid PKS-NRPSs. PKSs and PKS-NRPSs from the three Sigatoka disease complex species are highlighted in red. The domain architecture of each modular PKS and PKS-NRPS enzyme is plotted next to its name. The abbreviations used for each species are indicated in the legend. (TIFF) [file pgen.1005904.s017.tiff]

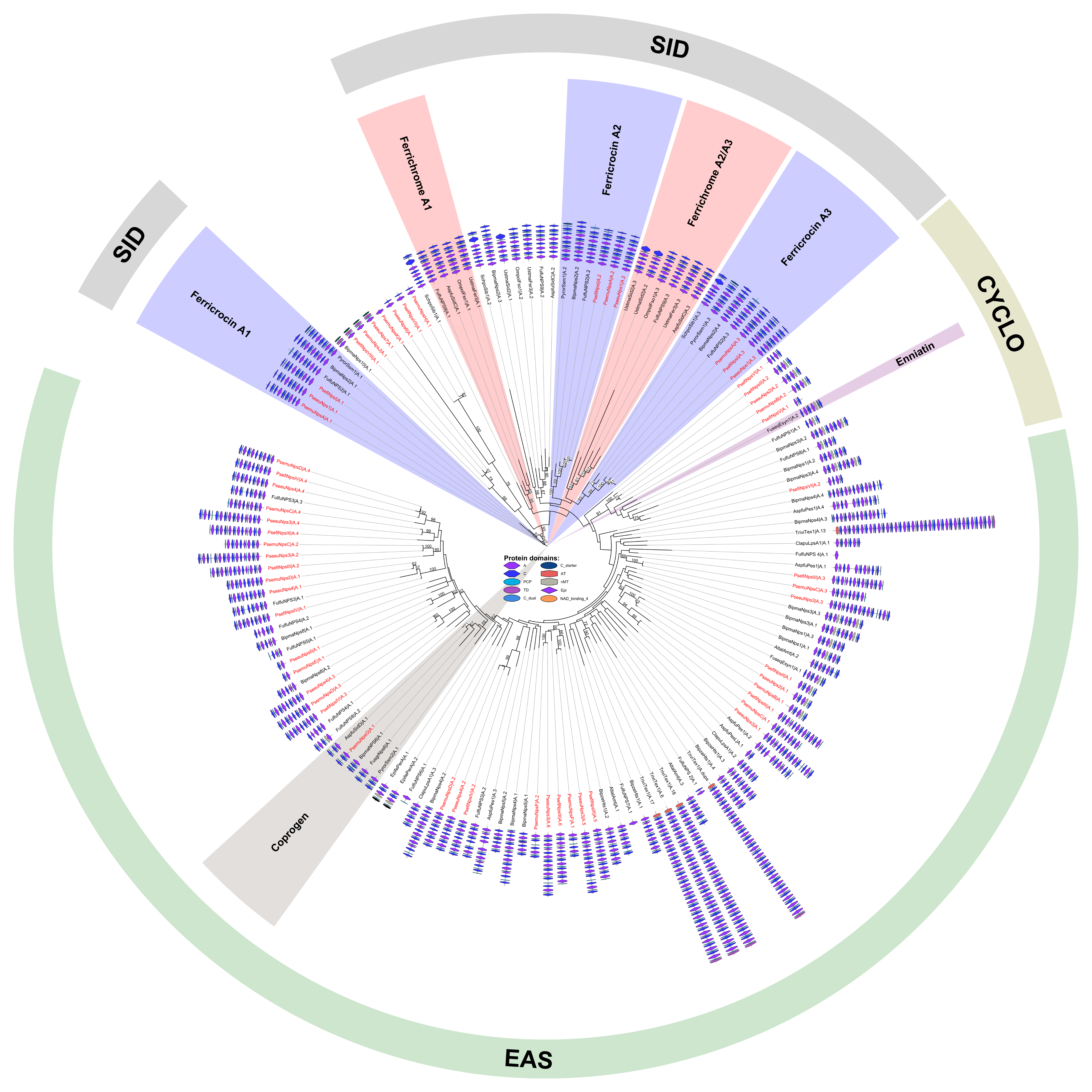

Supplement: S18 Fig — The phylogenetic tree is built based on the adenylation (A) domain sequences of each modular NRPS enzyme, using maximum likelihood with 1000 replications. Bootstrap values > 50% are labeled next to corresponding nodes. Well supported clades (bootstrap values >80%) that include NRPSs involved in the biosynthesis of known secondary metabolites (SMs) and toxins are shaded in different colors. NRPSs from the three Sigatoka disease complex species are highlighted in red. The domain architecture of each modular NRPS enzyme is plotted next to its name, whereas the outer ring represents the three major subfamilies to which NRPSs from P. musae, P. eumusae, and P. fijiensis can be classified, i.e. siderophore synthetases (SID), Euascomycete clade synthetases (EAS), and cyclosporin synthetases (CYCLO). The following abbreviations are used for each species: Psemu: Pseudocercospora musae, Pseeu: Pseudocercospora eumusae, Psefi: Pseudocercospora fijiensis, Altal: Alternaria alternata, Aspfu: Aspergillus fumigatus, Fulfu: Fulvia fulva, Clapu: Claviceps purpurea, Bipze: Bipolaris zeicola, Bipma: Bipolaris maydis, Epife: Epichloe festucae, Fuseq: Fusarium equiseti, Fusgra: Fusarium graminearum, Pyror: Pyricularia oryzae, Ompol: Omphalotus olearius, Schpo: Schizosaccharomyces pombe, Trivi: Trichoderma virens, Ustma: Ustilago maydis. (TIFF) [file pgen.1005904.s018.tiff]

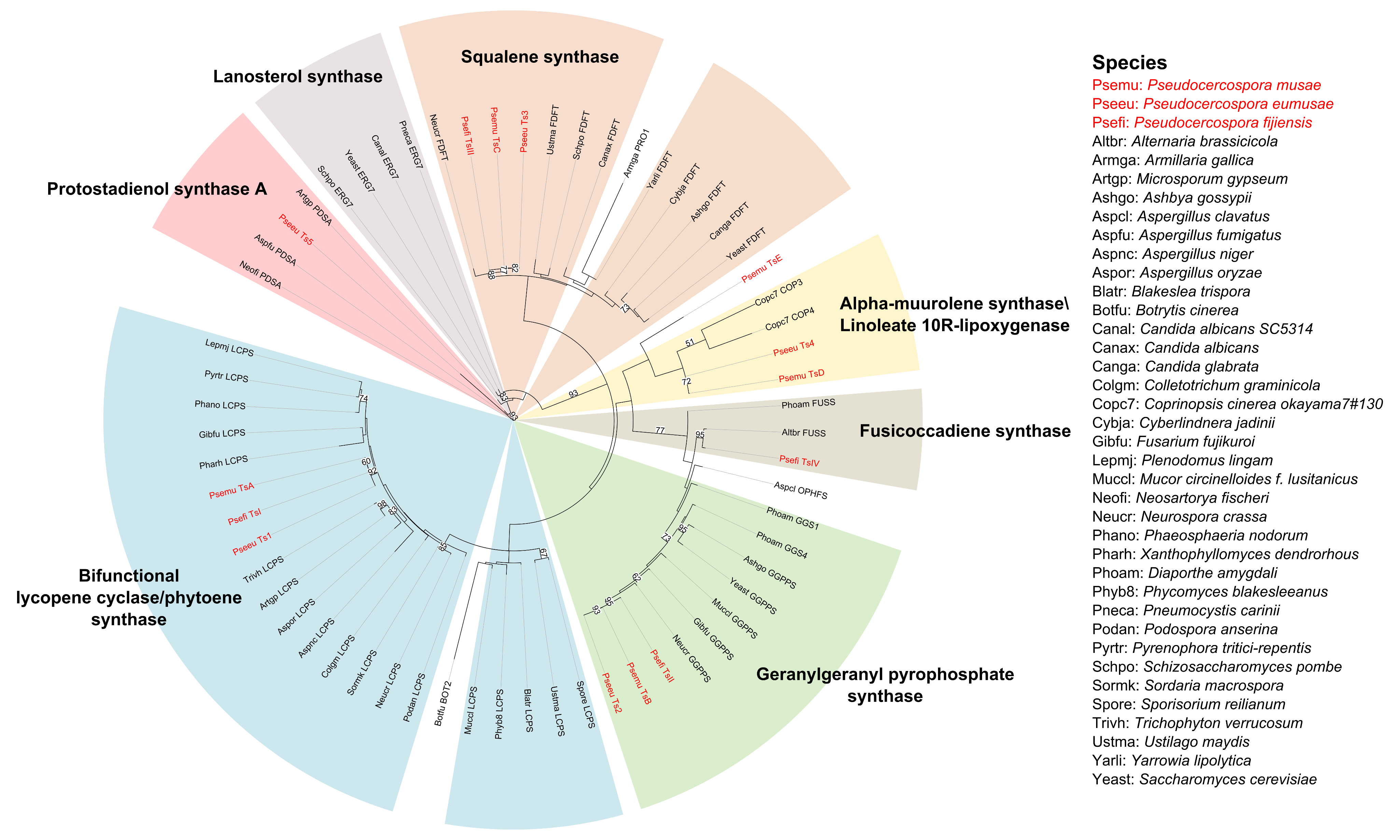

Supplement: S19 Fig — The phylogenetic tree is built based on an amino acid alignment of the full-length TSs, using maximum likelihood with 1000 replications. Bootstrap values > 50% are labeled next to corresponding nodes. Well supported clades (bootstrap values >80%) that include TSs involved in the biosynthesis of known secondary metabolites (SMs) and toxins are shaded in different colors. TSs from the three Sigatoka disease complex species are highlighted in red. The abbreviations used for each species are indicated in the legend. (TIFF) [file pgen.1005904.s019.tiff]

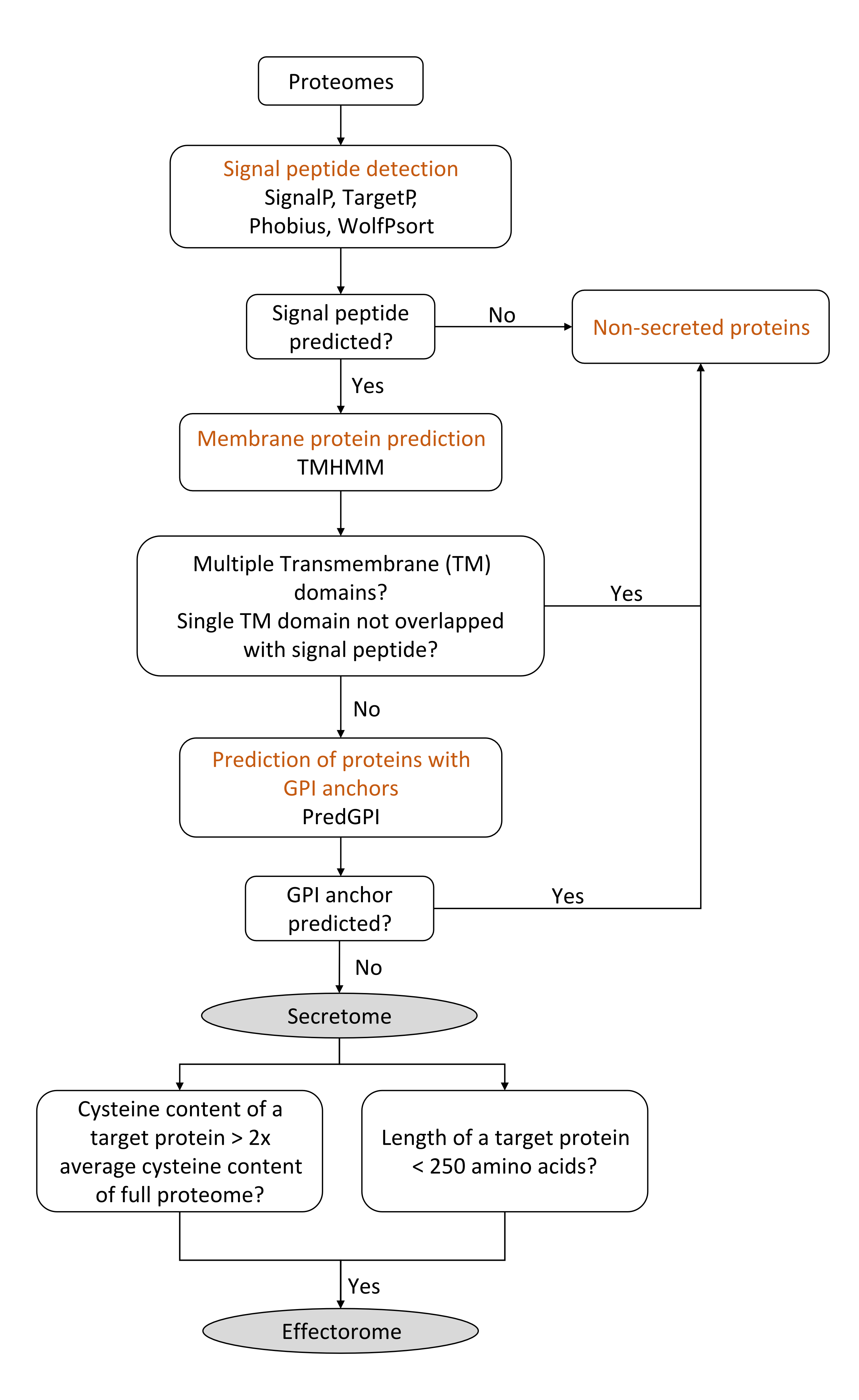

Supplement: S20 Fig — (TIFF) [file pgen.1005904.s020.tiff]

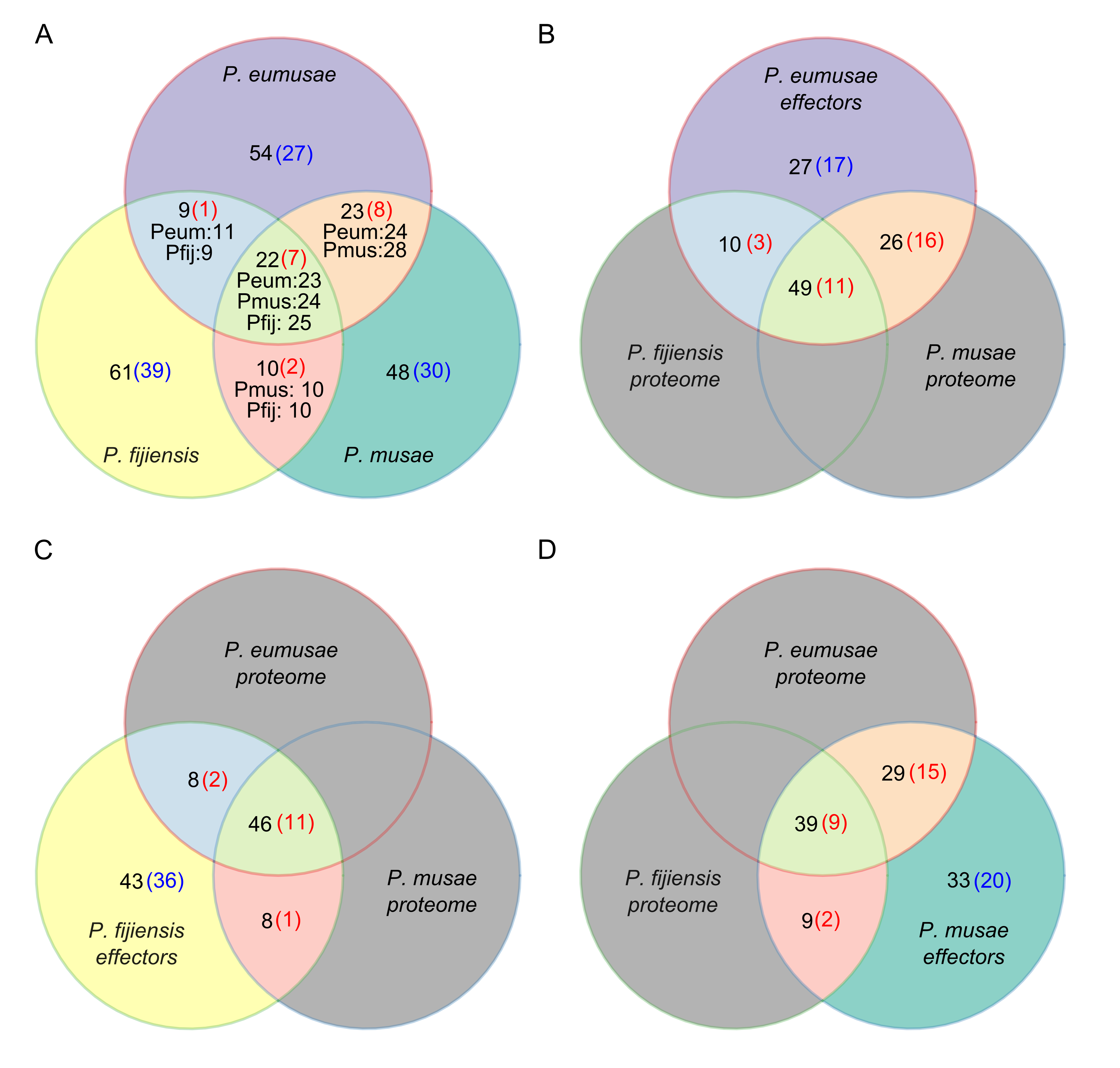

Supplement: S21 Fig — (A) Black numbers in the venn diagram show the total number of species-specific and shared effectors between and among the three species. Blue numbers in parentheses the species-specific sectors correspond to orphans (i.e effectors that do not have homologs in other fungi), whereas red numbers in parentheses in the intersections of shared effectors correspond to lineage-specific ones (i.e. effectors that are present only in at least two of the Sigatoka disease complex species). Reciprocal BlastP best hit (e-value: 1e-5) analysis implemented in OrthoMCL was used to retrieved the set of effectors shared by the three species, while BlastP (e-value: 1e-5, alignment coverage > 50%) against the NCBI nr database and the JGI fungal genome database was used to identify putative homologs in other fungal species and beyond. (B-D) The analysis is expanded to include a broader search for homologs of each species effectors against the entire proteome of the other two species. As before, black numbers correspond to comparisons among P. musae, P. eumusae, and P. fijiensis, while blue and red numbers in parentheses correspond to orphan and lineage-specific effectors, respectively. (TIFF) [file pgen.1005904.s021.tiff]

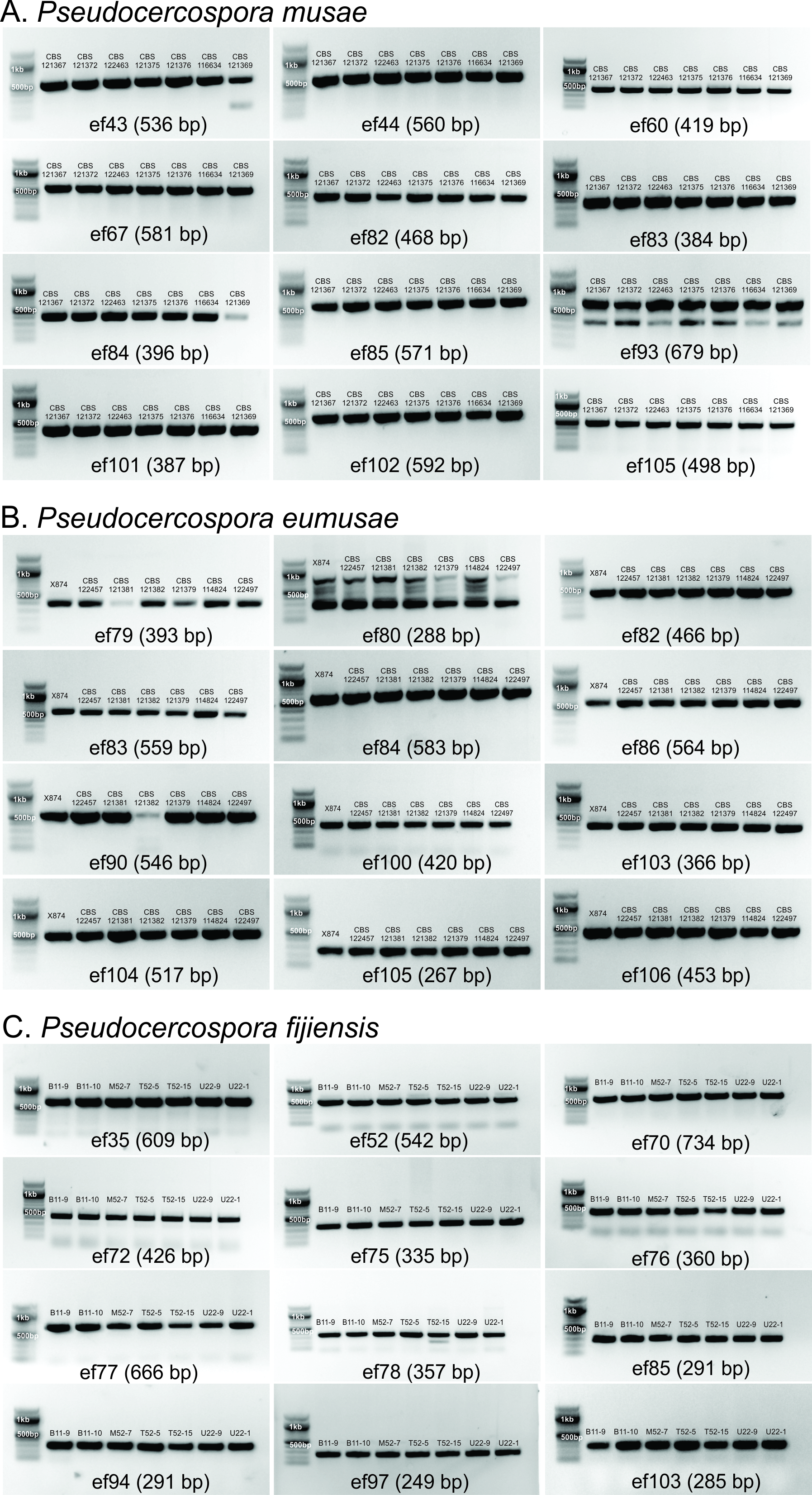

Supplement: S22 Fig — PCR and subsequent sequencing analysis of the amplified products (S10 Table) confirmed that the 12 randomly selected species-specific and orphan effectors are conserved in each species and, as expected, absent in the other two species. (TIF) [file pgen.1005904.s022.tif]

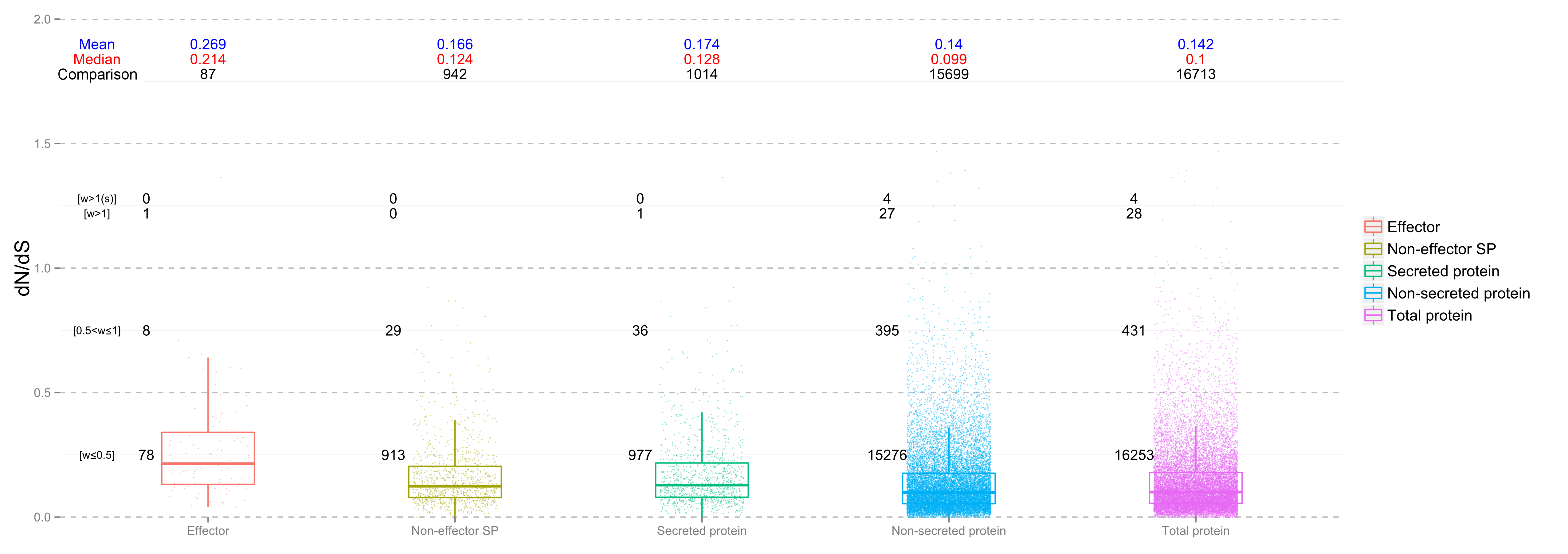

Supplement: S23 Fig — Pairwise dN/dS ratios (ω) (dots in the plot) were calculated for the gene families with one-to-one orthology in the three Sigatoka disease complex species. In order to examine whether different parts of the species proteomes are evolving under different rates, the gene families were partitioned into five groups, i.e those encoding for i) effectors, ii) non-effector secreted proteins, iii) secreted proteins, iv) non-secreted proteins, and iv) the total number of proteins. The mean, median, and total number of comparisons is shown at the top of each group. The dN/dS ratios were classified into three compartments along the y-axis (ω ≤ 0.5, 0.5 < ω ≤1, and ω > 1), in which the number of pairwise comparisons is denoted. In each group, an additional number (ω >1(s)) is denoted for the number of comparisons that have a dN/dS ratios > 1 and P-value < 0.05 based on Fisher’s exact tests. (TIFF) [file pgen.1005904.s023.tiff]

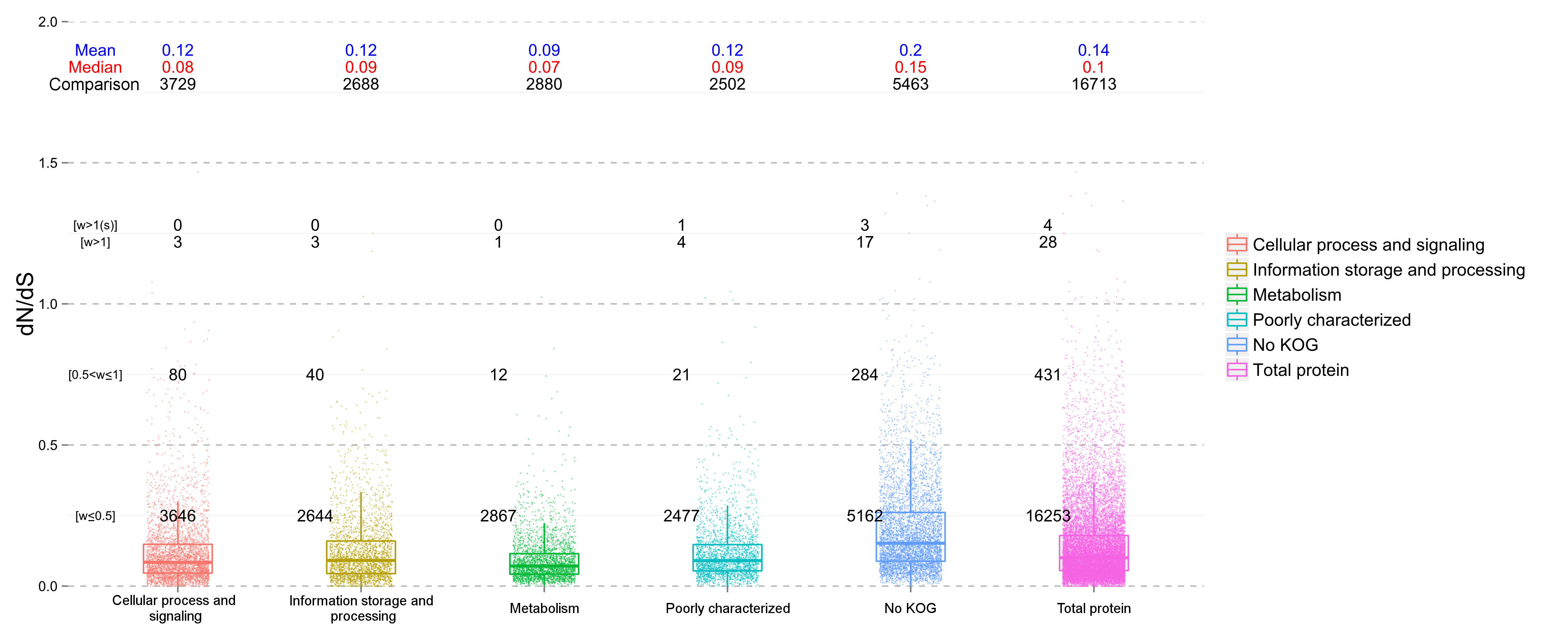

Supplement: S24 Fig — Pairwise dN/dS ratios (ω) (dots in the plot) were calculated for the gene families with one-to-one orthology in the three Sigatoka disease complex species. In order to examine whether different parts of the species proteomes are evolving under different rates, the gene families were partitioned into six groups based on the KOG functional assignment of their encoded proteins, i.e. i) cellular process and signaling, ii) information storage and processing, iii) metabolism, iv) poorly characterized, v) proteins with no KOG assignment, and vi) the total number of proteins. The mean, median, and total number of comparisons is shown at the top of each group. The dN/dS ratios were classified into three compartments along the y-axis (ω ≤ 0.5, 0.5 < ω ≤1, and ω > 1), in which the number of pairwise comparisons is denoted. In each group, an additional number (ω >1(s)) is denoted for the number of comparisons that have a dN/dS ratios > 1 and P-value < 0.05 based on Fisher’s exact tests. (TIFF) [file pgen.1005904.s024.tiff]

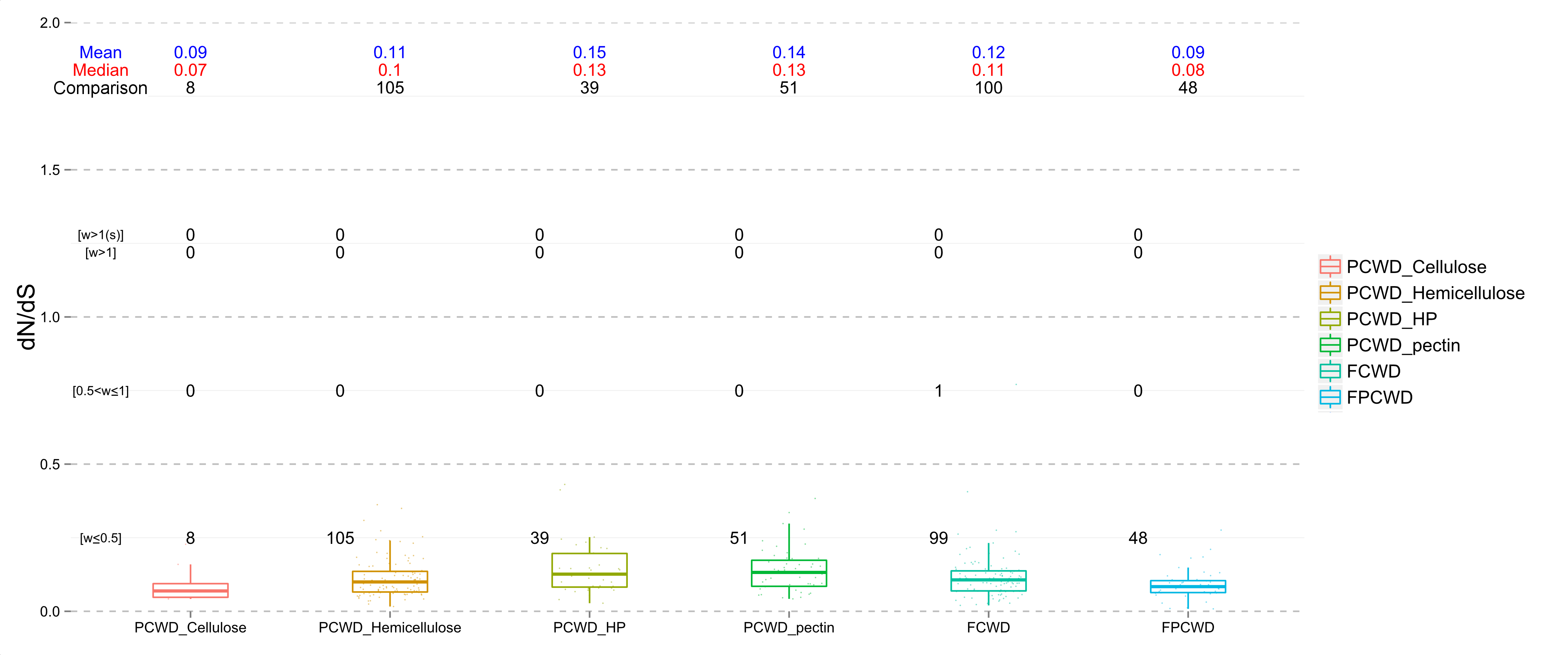

Supplement: S25 Fig — Pairwise dN/dS ratios (ω) (dots in the plot) were calculated for the gene families with one-to-one orthology in the three Sigatoka disease complex species. In order to examine whether different parts of the species CAZyomes are evolving under different rates, the gene families were partitioned into six groups based on whether their encoded enzymes are involved in the degradation of i) plant cell wall cellulose (PCWD-Cellulose), ii) plant cell wall hemicellulose (PCWD-hemicellulose), iii) plant cell wall hemicellulose-pectin (PCWD-HP), iv) plant cell wall pectin (PCWD-pectin), v) fungal cell walls (FCWD), and vi) fungal and/or plant cell walls (FPCWD). The mean, median, and total number of comparisons is shown at the top of each group. The dN/dS ratios were classified into three compartments along the y-axis (ω ≤ 0.5, 0.5 < ω ≤1, and ω > 1), in which the number of pairwise comparisons is denoted. In each group, an additional number (ω >1(s)) is denoted for the number of comparisons that have a dN/dS ratios > 1 and P-value < 0.05 based on Fisher’s exact tests. (TIFF) [file pgen.1005904.s025.tiff]
