# Supplementary material for: Comparative Genomics of the Sigatoka Disease Complex on Banana Suggests a Link between Parallel Evolutionary Changes in Pseudocercospora fijiensis and Pseudocercospora eumusae and Increased Virulence on the Banana Host
Source: PLoS Genet. 2016 Aug 11;12(8):e1005904. doi: 10.1371/journal.pgen.1005904 (PMC4981473; doi:10.1371/journal.pgen.1005904)
Supplement: S1 Table — (DOCX) [file pgen.1005904.s026.docx]

**S1 Table.** Genome assembly and annotation statistics.

| **Species** | ***Pseudocercospora musae*** | ***Pseudocercospora eumusae*** | ***Pseudocercospora fijiensis*^a^** |
| --- | --- | --- | --- |
| **Assembly statistics** |  |  |  |
| Assembly length (Mb) >500 bp | 60.44 | 47.12 | 74.14 |
| Assembly length (Mb) >2 Kb | 59.79 | 45.89 | 74.14 |
| Scaffolds | 3331 | 2626 | 56 |
| Scaffolds ≥2 Kb | 2879 | 1562 | 56 |
| Scaffold L50 | 353 | 109 | 5 |
| Scaffold N50 (Mb) | 0.04 | 0.16 | 5.9 |
| Estimated genome size (Mb) | 82.77 | 53.79 | n.a. |
| **Annotation statistics** |  |  |  |
| Predicted genes | 10632 | 11173 | 13107 |
| tRNA | 84 | 109 | n.a. |
| Coding | 10548 | 11064 | 13107 |
| Protein length (median) | 367 | 382 | 351 |
| Exon Length (median) | 441 | 458 | 152 |
| Gene length (median) | 1698 | 1636 | 1366 |
| Transcript length (median) | 1899 | 1680 | 1191 |
| Intron length (median) | 60 | 59 | 48 |
| Genes with intron | 7568 | 7670 | 9396 |
| Percentage genes with intron | 71.18% | 68.65% | 71.69% |
| Introns per gene with intron (median) | 2 | 2 | 2 |
| Exons per gene (median) | 2 | 2 | 2 |
| Intergenic distance (bp, median) | 547.5 | 552 | 662 |
| Total gene length (bp) | 26.9 | 24.6 | 22 |
| Total gene length (% of total genome) | 44.5 | 52.2 | 29.64 |
| Gene density (genes / Mb) | 175.91 | 237.12 | 176.79 |
| GC content of coding DNA (%) | 53.27% | 53.29% | 53.1 |

^a^ Data retrieved from Arango Isaza et al. (2015)
