# Supplementary material for: Comparative Genomics of the Sigatoka Disease Complex on Banana Suggests a Link between Parallel Evolutionary Changes in Pseudocercospora fijiensis and Pseudocercospora eumusae and Increased Virulence on the Banana Host
Source: PLoS Genet. 2016 Aug 11;12(8):e1005904. doi: 10.1371/journal.pgen.1005904 (PMC4981473; doi:10.1371/journal.pgen.1005904)
Supplement: S2 Table — (DOCX) [file pgen.1005904.s027.docx]

| **Classification** | | **Total bases covered ^a^** | | | | | | |
| --- | --- | --- | --- | --- | --- | --- | --- | --- |
| **Order** | **Superfamily** | ***Pseudocercospora musae*** | | ***Pseudocercospora eumusae*** | | | ***Pseudocercospora fijiensis*** | |
| **Class I (retrotransposons)** | | | | | | | | |
| LTR | Copia | 4,178,530 | ( 6.91, 14.33) | 660,955 | ( 1.40, 5.27) | 1,555,307 | | ( 2.10, 4.12) |
|  | Gypsy | 7,774,235 | (12.86, 26.66) | 4,059,038 | ( 8.61, 32.33) | 18,487,955 | | (24.94, 48.93) |
| DIRS | Ngaro | 374,383 | ( 0.62, 1.28) | 469,109 | ( 1.00, 3.74) | 0 | |  |
| Misc. LTR |  | 145,909 | ( 0.24, 0.50) | 0 |  | 1,476,078 | | ( 1.99, 3.91) |
| **subtotal** |  | **12,473,057** | **(20.64, 42.78)** | **5,189,102** | **(11.01, 41.34)** | **21,519,340** | | **(29.03, 56.95)** |
| LINE |  | 7,863,970 | (13.01, 26.97) | 2,384,542 | ( 5.06, 18.99) | 1,936,442 | | ( 2.61, 5.12) |
| PLE | Penelope | 0 |  | 107,177 | ( 0.23, 0.85) | 51,007 | | ( 0.07, 0.13) |
| **subtotal** |  | **7,863,970** | **(13.01, 26.97)** | **2,491,719** | **( 5.29, 19.85)** | **1,987,449** | | **( 2.68, 5.26)** |
| **Class II (DNA transposons) - subclass 1** | | | | | | | | |
| TIR | Tc1-Mariner | 202,684 | ( 0.34, 0.70) | 285,086 | ( 0.61, 2.27) | 505,674 | | ( 0.68, 1.34) |
|  | hAT | 117,423 | ( 0.19, 0.40) | 20,172 | ( 0.04, 0.16) | 4,180,522 | | ( 5.64, 11.06) |
|  | MuLE-MuDR | 64,187 | ( 0.11, 0.22) | 24,079 | ( 0.05, 0.19) | 281,256 | | ( 0.38, 0.74) |
|  | PiggyBac | 126,502 | ( 0.21, 0.43) | 38,874 | ( 0.08, 0.31) | 0 | |  |
|  | PIF-Harbinger | 266,534 | ( 0.44, 0.91) | 63,651 | ( 0.14, 0.51) | 309,435 | | ( 0.42, 0.82) |
| Misc. TIR |  | 323,916 | ( 0.54, 1.11) | 151,891 | ( 0.32, 1.21) | 654,630 | | ( 0.88, 1.73) |
| **Class II (DNA transposons) - subclass 2** | | | | | | | | |
| Helitron | Helitron | 0 |  | 0 |  | 508,164 | | ( 0.69, 1.34) |
| **subtotal** |  | **1,101,246** | **( 1.82, 3.78)** | **583,753** | **( 1.24, 4.65)** | **6,439,681** | | **( 8.69, 17.04)** |
| Low complexity |  | 19,170 | ( 0.03, 0.07) | 24,868 | ( 0.05, 0.20) | 31,401 | | ( 0.04, 0.08) |
| Satellite |  | 0 |  | 222,399 | ( 0.47, 1.77) | 0 | |  |
| Simple repeats |  | 1,556,674 | ( 2.57, 5.34) | 313,052 | ( 0.66, 2.49) | 444,128 | | ( 0.60, 1.18) |
| Unclassified repeats | | 6,143,959 | (10.17, 21.07) | 3,728,645 | ( 7.91, 29.70) | 7,363,356 | | ( 9.93, 19.49) |
| **Total** |  | **29,158,076** | **(48.24, 100.0)** | **12,553,538** | **(26.64, 100.0)** | **37,785,355** | | **(50.96, 100.0)** |

**S2 Table.** Annotation of transposable elements and other repeat sequences in *Pseudocercospora musae*, *Pseudocercospora eumusae,* and *Pseudocercospora fijiensis*.

^a.^ The first number in the parenthesis referred to percent of a specific repeat class in the genome assembly; the second number referred to the percent of a specific repeat class in the repeat regions
