# Supplementary material for: Comparative Genomics of the Sigatoka Disease Complex on Banana Suggests a Link between Parallel Evolutionary Changes in Pseudocercospora fijiensis and Pseudocercospora eumusae and Increased Virulence on the Banana Host
Source: PLoS Genet. 2016 Aug 11;12(8):e1005904. doi: 10.1371/journal.pgen.1005904 (PMC4981473; doi:10.1371/journal.pgen.1005904)
Supplement: S3 Table — (DOCX) [file pgen.1005904.s028.docx]

**S3 Table.** Summary statistics of repeat induced point mutation (RIP) in *Pseudocercospora musae*, *Pseudocercospora eumusae,* and *Pseudocercospora fijiensis*.

| **Genome features** | ***Pseudocercospora musae*** | ***Pseudocercospora eumusae*** | ***Pseudocercospora fijiensis*** |
| --- | --- | --- | --- |
| RIP loci | 5070 | 3820 | 2591 |
| Total RIP region length (Mb)^a^ | 31.97(53.5%) | 17.06 (37.2%) | 44.58(60.2%) |
| Repeat sequences with RIP signals (Mb)^b^ | 27.73 (99.89%) | 11.08 (99.73%) | 36.5(97.1%) |
| Proteins under RIP (overlap >250 bp) | 215 | 81 | 492 |
| Secreted protein under RIP | 20 | 5 | 13 |
| Effector under RIP | 2 | 1 | 4 |
| Proteins flanking RIP (<2 Kb) | 3164 | 2163 | 4539 |
| Secreted protein flanking RIP | 219 | 158 | 240 |
| Effector flanking RIP | 35 | 32 | 37 |

^a^ The number in the parenthesis indicates the percent of genomes covered by RIP loci

^b^ The number in the parenthesis indicates the percent of repeat regions with RIP signals
