# Supplementary material for: Comparative Genomics of the Sigatoka Disease Complex on Banana Suggests a Link between Parallel Evolutionary Changes in Pseudocercospora fijiensis and Pseudocercospora eumusae and Increased Virulence on the Banana Host
Source: PLoS Genet. 2016 Aug 11;12(8):e1005904. doi: 10.1371/journal.pgen.1005904 (PMC4981473; doi:10.1371/journal.pgen.1005904)
Supplement: S12 Table — (DOCX) [file pgen.1005904.s037.docx]

**S12 Table.** Sequences of the primers that were used to amplify a set of 12 species-specific or core effectors from field isolates of *Pseudocercospora musae*, *Pseudocercospora eumusae,* and *Pseudocercospora fijiensis.*

| **Target effector** | **Primer sequence** | **Product length (bp)** |
| --- | --- | --- |
| *Pseudocercospora musae* | | |
| Ef43 | F: ATG CGT GGC TTT ACA GTC  R: TCA ATT CGG TGC AAG TCC | 536 bp |
| Ef44 | F: ATG CAA TTT TCA CTC GTC GTG  R: CTA CGA TCG CTG ACA CAC AAG | 560 bp |
| Ef60 | F: CAA CAT GAA GGC CAA CAC AAT C  R: AAT CCA AGC CCT TTT ACT TCC C | 419 bp |
| Ef82 | F: ATG CAT TCC TTG TCT ATC CTC  R: CTA ACA TCT CTG CTT AGG GG | 468 bp |
| Ef83 | F: ATG CAA CAC CTC ACC ATC ACC  R: CTA TTC CGG ACA ATT CAC C | 384 bp |
| Ef84 | F: ATG CGA GGT GCG CAT AAC  R: TAA GCC GAG AGG CCA TCT ATC | 396 bp |
| Ef85 | F: ATG CGC TCC CTC ACG C  R: TCA CAT TCC AAT CTT CAT CTT AG | 571 bp |
| Ef93 | F: ATG GCC GTG ATC GTC CTG  R: TTA TCT TGT GGC TGG TGG ATC | 679 bp |
| Ef100 | F: ATG CCT GCG CAC TAC TTG  R: TCA ACA TCC TGA ATA GCA C | 396 bp |
| Ef101 | F: ATG CGC TTG ATC GAC ATC  R: GAC GTC TAT GTT GCA GGA CTG | 387 bp |
| Ef102 | F: ATG AAG TCC CTC CTC ACC  R: TTA TCC AAC CCC GGA CAC C | 592 bp |
| Ef105 | F: ATG CTC AGC TTC GGT TTC AAG  R: TCA TCC CGT GTC GCT GGT C | 498 bp |
| *Pseudocercospora eumusae* | | |
| Ef79 | F: ATG TTT AGT AGA GCT TGG G  R: GTC AGC GAA GCT CAA CTT G | 393 bp |
| Ef80 | F: ATG CTG ATT CAG CTC GCC  R: GAT TTC TGT TTG GGA CGA C | 288 bp |
| Ef82 | F: ATG CAG TTT ACC ACG ACA TCC  R: CTA GGA TCC ACA GTT GCT G | 466 bp |
| Ef83 | F: ATG AAT ATC GAA GAG ATT CTG  R: GAG CTC ACG TCA TGA TAC C | 559 bp |
| Ef84 | F: ATG CGA CTC TAC AGC ACG C  R: TCA GTG GCA CGG ACA TGG | 583 bp |
| Ef86 | F: ACA ATA TGC ATC TCC ACG C  R: TCA AAT ACA CTG CTG ACA AG | 564 bp |
| Ef90 | F: ATG TCC GCG AAT GTG ATT TTC C  R: TCA CGA GGT CAA CTT CAC TCC | 546 bp |
| Ef100 | F: ATG AAG CTC TCA GCC TGG  R: TCA TGG CCA ATA CTT AAC C | 420 bp |
| Ef103 | F: ATG CAT TTC AGC TCC CCA AAT C  R: TCA CTC GCA ATC GAC AGC | 366 bp |
| Ef104 | F: ATG CAT TAT ATC AAG TCG ATT C  R: TCA TTT GCA CCT CCT AGC G | 517 bp |
| Ef105 | F: ATG AAG ACC ACA CAA TAC GG  R: CTA AGC CTT GGT GCA ACC | 267 bp |
| Ef106 | F: ATG CTG CTT CTA CTT CAA CTC  R: AAC GAC TCT CAA CAC GTA GG | 453 bp |
| *Pseudocercospora fijiensis* | | |
| Ef35 | F: TCC TTC CCA CAT TTG CAA TG  R: CAA CAG AGA GTC GAA AAG CT | 609 bp |
| Ef52 | F: GAT TTG CAC CCA TGA ATA TGG  R: GAA GTG TCC TTG TAG TCA C | 542 bp |
| Ef70 | F: ATG TGC GTC ACG CAT TGC TC  R: GTA TAT CAT TTG ACC ACT GTC | 734 bp |
| Ef72 | F: ATG CTG CTG ATT ATG CTG CTG  R: ATC CTG CCG CTA CCA TCA CC | 426 bp |
| Ef75 | F: AAG ATG CAT TAC GCA GTT ATC C  R: TGA CAT GTA ACC TTG TAC GAA | 335 bp |
| Ef76 | F: ATG CAT TAC GCA GTT ATC TTT G  R: TTA TAT AGG GTG GCG ACA TG | 360 bp |
| Ef77 | F: CAT CAT GAA GTC CAT TCT TAT C  R: TTA ATT CTG GCA AGG ACT C | 666 bp |
| Ef78 | F: GAA TGG CAG TCA TGG CTA G  R: ATT GCC CTT GTC AGC AGG | 357 bp |
| Ef85 | F: ATG AAT GAT ATG AGC GCA CG  R: TTA GTG CAC TTT CCA GGG G | 291 bp |
| Ef94 | F: ATG AAT GAT TGT GGT AGG TC  R: TTC AAG TTG CTA CAG TAC C | 291 bp |
| Ef97 | F: ATG ATG CAC TTT CGA CCG TT  R: TTA TCG AGG ACA AAA GCT AGC G | 249 bp |
| Ef103 | F: TGT ATG TCG TCC TGC TTT CC  R: TAG GAA GGA AAG GAA GTT GTC | 285 bp |
