# Supplementary material for: Comparative Genomics of the Sigatoka Disease Complex on Banana Suggests a Link between Parallel Evolutionary Changes in Pseudocercospora fijiensis and Pseudocercospora eumusae and Increased Virulence on the Banana Host
Source: PLoS Genet. 2016 Aug 11;12(8):e1005904. doi: 10.1371/journal.pgen.1005904 (PMC4981473; doi:10.1371/journal.pgen.1005904)
Supplement: S1 Text — Supporting Results includes additional information and discussion on the different analyses that have been performed. The following theme paragraphs (P) are included: P1: Analysis of syntenic relationships reveals strong locally conserved gene order and content, interrupted by repeat elements. P2: The three Sigatoka species display mark differences in their repertoire of transposable elements (TEs). P3: The efficacy and specificity of RIP in transposable elements and beyond differs among the three species. P4: Functional annotation and characterization of the species’ gene complement indicate abundant species- and lineage-specific adaptations. P5: Analysis of copy-number variations (CNV) reveals parallel patterns of gene family expansions and contractions between P. fijiensis and P. eumusae. P6: CAZy annotations and characterization of plant cell wall degrading enzymes (PCWDEs) suggest small differences among the three species but also more similar profiles for P. eumusae and P. fijiensis as compared to P. musae.P7: Annotation of the core enzymes involved in the biosynthesis of secondary metabolites (SMs) reveals that the three Sigatoka species potentially produce a diverse but only partially overlapping array of SMs. P8: Effector characterization indicates that the three pathogens exhibit overlapping but still very dissimilar repertoires of candidate effectors. P9: Supporting References. (PDF) [file pgen.1005904.s038.pdf]

S1 Text: Supporting Results

Analysis of syntenic relationships reveals a pattern of "broken" macrosynteny among the genomes of the three species.

To identify genome-wide structural variation among the three species that constitute the Sigatoka disease complex, the *P. musae* and *P. eumusae* scaffolds were aligned and ordered using the *P. fijiensis* scaffolds as a reference. Whole genome alignment between *P. musae* and *P. eumusae* was not informative, due to the highly fragmented assemblies of these two species. Of the 56 scaffolds that constitute the genome assembly of *P. fijiensis*, only scaffolds 1-10, scaffold 12 and scaffold 19 were found to hold syntenic relations with scaffolds in *P. musae* and/or *P. eumusae*. These scaffolds could thus represent the core chromosomes in *P. fijiensis*, while the remaining scaffolds are possibly derived from dispensable chromosomes, in agreement with what has been previously proposed based on an analysis of syntenic relations between *P. fijiensis* and *Z. tritici* [1].

A total of 366 syntenic blocks were identified between *P. musae* and *P. fijiensis*, covering 34% (20.3 Mb) and 55% (40.8 Mb) of the genome assembly in *P. musae* and *P. fijiensis*, respectively. Nearly all of the syntenic blocks are rather small in size (9.1-279.9 Kb, median 41 Kb) and, consequently, each contains only a small number of orthologous gene pairs (average 20, median 15, total 7306). To get a better picture of the syntenic relationships between the two species we performed dot-plot analysis of the 11 scaffolds in *P. musae* that were larger than 200 kb in size than those in *P. fijiensis*. The analysis revealed the presence of segmental and tandemly repeated blocks of synteny between the two species that was occasionally combined with intra-chromosomal inversions (S3B Fig.). This pattern can be potentially interpreted as "broken" or "segmented" macrosynteny. Along the same lines, alignment of *P. eumusae* and *P. fijiensis* genomes identified 259 syntenic blocks indicating that, despite their small size (8.9-750.8 Kb, median 57.4 Kb) and number of orthologous gene pairs contained in them (average 32, median 21, total 8388), they share a high degree of localized co-linearity and synteny. Moreover, syntenic blocks between these two species accounted for 49% of the genomic content in *P. eumusae* and 64% in *P. fijiensis.* Thus, as compared to *P. musae*, *P. eumusae* shares a higher degree of conservation to *P. fijiensis* in terms of coverage of syntenic sequences. The dot plot analysis of the 38 scaffolds in *P. eumusae* that were larger

than 200 kb in size compared to those in *P. fijiensis* again revealed a pattern of putatively broken macrosynteny between the two species (S3C Fig.).

Taken together, analysis of syntenic relations indicated that, as compared to other species of Dothideomycetes [1], the three primary agents of the Sigatoka disease complex share a higher portion of localized conservation of gene order that further extents to segmental and tandemly repeated blocks of macrosynteny, most likely as a result of the lineage-specific proliferation of repetitive elements in the three species and other genomic rearrangements.

The three Sigatoka species display mark differences in their repertoire of transposable elements (TEs).

Transposable elements (TEs) are a major component of fungal genomes, the dynamics of which largely define variations in genome sizes among different fungal species, thus contributing to their evolution. Genome-wide annotation and comparative analysis of transposable elements in *P. musae*, *P. eumusae* and *P. fijiensis* indicated that, as with other Dotyhideomycetes [2-4], Class I TEs account for the majority of the repetitive content in each genome (*Pm:* 20.4/29.2 Mb, 69.8%; *Pe:* 7.7/12.6 Mb, 61.1%; 62.3% *Pf*: 23.5/37.3 Mb), followed by Class II elements, which occupy a considerably higher fraction of the repetitive fraction in *P. fijiensis* (6.4/37.3 Mb, 15.9%) as compared to *P. musae* (1.1/29.2 Mb, 3.8%) and *P. eumusae* (0.6/12.6 Mb, 4.7%).

Next to the different representation of Class I and Class II elements in the repetitive fraction of the three species, differences were also observed when considering variations in the repertoire of TEs within each of the two major Classes. For example, in spite of the fact that Class I elements occupy a similar fraction of the repetitive content in each species, marked differences in fractions of Long Terminal Repeat (LTR) and non-LTR retrotransponsons, the two major subclasses that make up Class I elements, were present among the three species. LTR retrotransposons, more specifically, are the most numerous retroelements in all three genomes, but their fraction is much higher in *P. fijiensis* (21.5 Mb, 57.7%) as compared to *P. musae* (12.5 Mb, 42.8%) and *P. eumusae* (5.2 Mb, 41.3%) (Fig. 2B; S2 Table). Among LTR retrotransposons, elements of the *Ty3/Gypsy* family are the most abundant in both *P. fijiensis* (18.5 Mb, 48.9%) as well as in *P. musae* (7.8 Mb, 26.7%) and *P. eumusae* (4.1 Mb, 32.3%), followed in much lower proportion by elements of the Ty1/Copia family (*Pm*: 4.2 Mb, 14.3%; *Pe*: 0.6 Mb, 5.3%; *Pf*: 1.6 Mb, 4.1%)

(S2 Table). With respect to non-LTR retrotransposons, the long interspersed elements (LINEs) were the dominant repeat class and were particularly enriched in *P. musae* (7.9 Mb, 27%) and *P. eumusae* (2.4 Mb, 19%). In contrast, LINE elements make up just 5.1% (2 Mb) of the repetitive fraction in *P. fijiensis* (Fig. 2B; S2 Table). Among Class II elements, a large expansion of the TIR/hAT transposase is apparent in the genome of *P. fijiensis* (4.2 Mb, 11.1%) but this subfamily is only limitedly present in *P. eumusae* (0.02 Mb, 0.2%) and *P. musae* (0.1 Mb, 0.4%) (S2 Table). Finally, several cases of species-specific gains and losses of particular types of Class I and Class II elements were observed. For example, the Class I DIRS Nagro and Penelope-like (PLE) elements were absent from *P. eumusae* and *P. musae*, respectively, although they were both present in *P. fijiensis*. Also, the Class II PiggyBac element was found present only in *P. eumusae* and *P. musae*, while the Helitron element was only in *P. fijiensis* (S2 Table). Next to the well characterized Class I and Class II TEs, a considerable amount of unclassified repeat elements that represent newly evolved, species-specific repeats were also discovered in the genomes of the three species. Notably, unclassified repeats occupied a higher fraction of the repetitive content in *P. eumusae* (3.7/12.6 Mb, 29.7%), as compared to *P. musae* (6.1/29.2 Mb, 21.1%) and *P. fijiensis* (7.4 Mb/37.3, 19.8%), consistent with the later historical appearance of this species (Fig. 2B; S2 Table).

The efficacy and specificity of RIP in transposable elements and beyond differs among the three species.

In addition to impacting genome evolution, differences in the repertoire of TEs among the three species also imply differences in TE activity and possibly genome defenses against mobile genetic elements. A major defense mechanism against TE activity in fungi is mediated by repeat-induced point mutation (RIP) [5, 6], a homology-based process that causes C:G to T:A transitions to duplicated regions of DNA during meiosis, thus rendering TEs inactive through mutation [7]. RIP can also affect other types of DNA elements, including gene duplicates that are frequently inactivated by the RIP-mediated introduction of stop codons [8].

Analysis by RIPCAL [9] indicated that the genomes of the three species are all subject to RIP (S3 Table). Overall, *P. musae* has a higher number of RIP loci predicted in its genome (5070), followed by *P. eumusae* (3820) and *P. fijiensis* (2591). However, given the differences in genome sizes, a larger fraction of the *P. fijiensis* (60.2%, 44.58 Mb) and *P. musae* (53.5%, 31.97 Mb) genomic sequences are under RIP as

compared to *P. eumusae* (37.2%, 17.06 Mb). In all three genomes RIP occurred mainly on large repeat sequences (> 500 bp) as the vast majority (~98% on average) shows signs of RIP. Such high levels of RIP in repeat sequences are comparable to the levels reported for other Dothideomycetes, including *F. fulva* (97.2%), *P. lingam* (99.8%), *Z. tritici* (97.9%), and others [2-4]. Notably, they are also inconsistent with the high density of TEs in the genomes of the three Sigatoka complex species, perhaps suggesting that RIP cannot effectively defend them against TE activity. Furthermore, while the majority (2449/2591, 94.5%) of the putative RIP loci in *P. fijiensis* are co-localized with the identified repeat elements, in contrast, 10% (518/5070) of the RIP loci in *P. musae* and an impressive 33% (1257/3820) in *P. eumusae* are not associated with repeat elements. This could suggest abundant spillage of RIP in *P. eumusae* outside the duplicated target sequence into the adjoining non-duplicated sequences and/or that multicopy genes in *P. eumusae* may be more sensitive to RIP than in *P. musae, P. fijiensis,* and other Dothideomycetes [2, 3, 10]. Furthermore, 2163, 3164, and 4539 of putative protein-coding genes that represent 19.4%, 29.8%, and 34.6% of the total genes in *P. eumusae, P. musae,* and *P. fijiensis,* respectively, were found located within a 2 kb region flanking the RIP loci in each species. These included 32 (28.6% of the total predicted effectors), 35 (31.8% of the total predicted effectors), and 37 (35.2% of the total predicted effectors) of putative effectors present in *P. eumusae*, *P. musae*, and *P. fijiensis*, respectively (S3 Table). Slippage of RIP in the coding regions of effectors could impact pathogenicity, as has been demonstrated in the pathogenic fungus *Pl. lingam* [11].

Functional annotation and characterization of the species' gene complement indicate abundant species- and lineage-specific adaptations.

To understand similarities and differences in the functional properties of the three species, we annotated each species proteome, by assignment into the functional categories of the eukaryotic orthologous groups (KOG) database [12]. KOG has four major functional categories, i.e. cellular processing and signaling, information storage and processing, metabolism, and poorly characterized proteins with unknown functions. A total of 6292 (59.7%), 6783 (61.3%), and 7323 (55.9%) of the predicted proteins in *P. musae*, *P. eumusae*, and *P. fijiensis*, respectively were assigned to KOGs. A KOG-based breakdown of the species' proteomes indicated that the percentage of proteins allocated to each of the four higher functional categories of KOG was comparable among the three species, although the absolute total number of

proteins assigned to the same KOG category could be different among them (S6 Fig.). For example, a total of 1873 proteins from *P. musae*, 2066 proteins from *P. eumusae* and 2164 from *P. fijiensis,* corresponding to 17.8%, 18.7% and 16.5% of their proteomes, respectively, were assigned KOGs in the cellular processes and signaling category. Similar results were also obtained when examining the distribution of KOGs from each species within the functional categories of metabolism (*Pm*: 1946 proteins, 18.45%; *Pe*: 2026 proteins, 18.31%; *Pf*: 2235 proteins, 17.05%), information storage and processing (*Pm*: 1188 proteins, 11.26%; *Pe*: 1312 proteins, 11.85%; *Pf*: 1413 proteins, 10.78%), and the category of poorly characterized ones (*Pm*: 1285 proteins, 12.18%; *Pe*: 1379 proteins, 12.46%; *Pf*: 1511 proteins, 11.53%) (S6 Fig.). The pattern was also conserved when the proteomes were annotated based on the 25 subcategories of KOG, in which case *P. fijiensis* generally exhibited the highest number of proteins annotated in all but three of the sub-categories (i.e. N: Cell wall/membrane envelope biogenesis, Y: Posttranslation modification, protein turnover, chaperones, and B: Replication, recombination and repair). Proportionally to their proteome sizes, however, the three species do not exhibit any significantly large differences in the percentage of proteins distributed across the 25 KOG subcategories (S6 Fig.), indicating that, based on their KOG profiles, they execute a fairly similar spectrum of biological activities.

Further orthology-based comparative analysis of the species' gene and proteome complements indicated that a total of 6307 protein-coding gene families containing at least one gene copy in each of the three species were shared by all three species that represent their core proteome complement (Fig. 4A). KOG-based functional annotations revealed that nearly a third (2076) of the core gene families encode hypothetical proteins that could not be assigned to any of the four higher-level categories of KOG. A total of 4782 KOG terms could be assigned to the remaining 4231 families, with the ones involved in "cellular processing and signaling" (1508), and "metabolism" (1311) being more abundant as compared to families involved in "information storage and processing" (1008) or are "poorly characterized" families with unknown function (955) (S7 Fig.). Note that some gene families receiving KOG annotations could be assigned to more than one functional category of KOG. Thus, the distribution of KOG annotations for the core proteome of the three species follows a pattern similar to that of their individual full proteomes. Perhaps not surprisingly based on the phylogenetic relationships of the three species, the proportion of the *P. fijiensis* proteome (6975/13 107, 53.21%) included within the core proteome of the three species is smaller as compared to *P. eumusae* (6886/11 064, 62.23%) and *P. musae* (6834/10 548, 64.78%),

indicating that the overall gene complement of this species is more divergent as compared to the gene complements of the other two pathogens. Consequently, the number of species-specific protein-coding genes retrieved from *P. fijiensis* (3442/13 107, 26.2%) was higher as compared to *P. eumusae* (1759/11 064, 15.9%) and *P. musae* (1867/10 548, 17.7%) (Fig. 4A), which is in line with the earlier branching of *P. fijiensis* from the last common ancestor of all three species [13]. The KOG-based functional annotations of the species-specific genes revealed that the overwhelming majority of these genes in *P. musae* (1652), *P. eumusae* (1460), and *P. fijiensis* (2842) encode for hypothetical proteins with unknown function (S7 Fig.). Such genes may have been acquired after speciation events and perform novel functions, potentially contributing significantly to the genome evolution and pathogenic diversification of the three species [14].

A BLAST-based search (e-value: 1e-5, alignment coverage > 50%) against all currently available fungal genomes in the JGI database revealed that from the 6307 core protein-coding gene families, 234 are lineage-specific to the Sigatoka species (Fig. 4B). Such gene families could embrace genes that are important for virulence on the banana host, and are thus suitable candidates for follow-up functional analyses or for use as molecular markers. Functional annotations showed that only 55 of the lineage-specific gene families could be assigned to one of the functional categories of KOG, while 17 of the families were predicted to encode for secreted proteins, including 6 putative effectors that could be required for virulence specifically on the banana host (S8 Fig.). Similarly, of the species-specific genes, 2176, 1403, and 1120 genes in *P. fijiensis, P. musae* and *P. eumusae,* respectively, are putative orphans, as no homologs could be identified in none other species (Fig. 4B). The overwhelming majority of orphan genes (~95%) in the three pathogens encode for hypothetical proteins without any protein domains or putative functional roles assigned to them (S8 Fig.), suggesting that they may promote micro-evolutionary divergence of the three species and likely virulence on the banana host as well.

Analysis of copy-number variations (CNV) reveals parallel patterns of gene family expansions and contractions between *P. fijiensis* and *P. eumusae*.

Analysis of CNV among *P. musae, P. eumusae*, and *P. fijiensis* indicated that clustering of the species based on the pattern of expansions and reductions in core gene families, and especially the ones related to metabolism, is more respectful of the species virulence profiles rather than their evolutionary

relationships, suggesting that *P. fijiensis* and *P. eumusae* exhibit somewhat concerted patterns of CNV (Fig. 6).

To investigate whether the pattern of parallel changes in size in gene families shared between *P. fijiensis* and *P. eumusae* extended beyond core gene families, the analysis was expanded to include gene families that are shared by at least two of the species but not necessarily the third one (i.e. it could be absent in the third species). The number of equally sized gene families between either two of the three species was then enumerated and further classified into three major groups, based on the pair of species that were sharing the equivalent family sizes. For example, in group *Pe*/*Pf*, *P. eumusae* and *P. fijiensis* share equal family sizes but different than *P. musae,* in group *Pe*/*Pm*, *P. eumusae* and *P. musae* share equal family sizes but different than *P. fijiensis*, and finally in group *Pm*/*Pf*, *P. musae* and *P. fijiensis* share equal family sizes but different than *P. eumusae*. To avoid uncertain CNV status, species-specific genes were not considered in this analysis. Once more, the pairwise comparisons showed that a significantly higher number of the gene families had exactly the same copy number shared between *P. eumusae* and *P. fijiensis* (*Pe*/*Pf group*: 1742 gene families), rather than between *P. musae* and *P. fijiensis* (*Pm*/*Pf* group: 1127 gene families) or between *P. musae* and *P. eumusae* (*Pe*/*Pm* group: 945 gene families) (S9 Fig.). This result reinforces the confidence that *P. eumusae* and *P. fijiensis* share a more similar pattern of gene family expansions and contractions as compared to the other two pathogen pairs. KOG-based functional annotations showed that of the gene families with CNV that can be assigned to a functional category of KOG (2363 families), the majority are again mainly related to metabolism (33.0%, 780/2363 families), followed by cellular processes and signaling (29.3%, 692/2363 families) and information storage and processing (16.7%, 395/2363 families). Furthermore, for the families with CNV, *P. eumusae* and *P. fijiensis* retain comparable copy numbers in almost all categories and subcategories of KOG as compared to the other two species pairs.

Although clustering of *P. fijiensis* together with *P. eumusae,* when considering variations in gene families related to metabolism, could be due to convergent expansions and contractions in these two species, an alternative hypothesis is that it might be caused by changes that have taken place in *P. musae.* To investigate this possibility, we followed an approach of observing in the nine Capnodiales species that were previously used for phylogenetic reconstruction and estimation of divergence times (Fig. 3, S5 Fig.),

changes in gene copy numbers of their metabolic families. In specific, we first identified, based on KOG annotations, gene families associated with metabolism in the nine Capnodiales species, and then using the CNV for each family among the different species, we performed hierarchical clustering in order to elucidate the pattern of copy number changes that has emerged during evolution. Hierarchical clustering of the species based on copy number changes in the 1503 metabolic gene families that were identified in at least one of the nine species, clustered *P. eumusae* together with *P. fijiensis*, supporting the occurrence of parallel evolution (S10A Fig.). In addition, *P. musae* still clustered together with *P. eumusae* and *P. fijiensis* (bootstrap value of 68), and not in another part of the dendrogram, suggesting that changes in gene copy numbers in this species are not happening at a rate that is radically different from the other species of Capnodiales included in the analysis. Changes in copy numbers among the 9 species of Capnodiales occurred in 922 of the 1503 metabolic gene families, including 130 gene families that have changed copy number only in *P. musae,* and which consequently could have directed the clustering of *P. fijiensis* together with *P. eumusae.* However, excluding these 130 gene families from the analysis still clustered the two more virulent species together (S10B Fig.), indicating that their clustering is likely due to parallel expansions and contractions in these two species rather than changes that took place solely in *P. musae.*

To further investigate which metabolic pathways are likely to have been affected by parallel changes in the two more virulent species, we performed a GO (Gene Ontology)-based analysis and identified GO terms that support the clustering of *P. eumusae* with *P. fijiensis*. Genome-wide GO-based functional annotations were done using InterProScan. A total of 4590 (41.5%) proteins of *P. eumusae*, 4277 (40.5%) proteins of *P. musae,* and 4930 (37.6%) proteins of *P. fijiensis* could be assigned at least one GO term in one of the three higher-level ontology categories, i.e. biological process (BP), molecular function (MF) and cellular component (CC). The majority of proteins from all three species were assigned GO terms in the BP category (*P. eumusae*: 6440, *P. musae*: 6079, and *P. fijiensis*: 6775), followed by assignments in the MF (*P. eumusae*: 5094, *P. musae*: 4791, and *P. fijiensis*: 5304) and CC categories (*P. eumusae*: 3776, *P. musae*: 3561, and *P. fijiensis*: 4035). In the BP category, GO terms for metabolic (GO: 0008152) and cellular processes (GO: 0009987) were particularly enriched in the genomes of the three species, while in the MF and CC categories, GO terms for catalytic activity (GO: 0003824) and binding (GO: 0005488), and GO terms for cell (GO: 0005623) and organelles (GO: 0043226), respectively, were the ones mostly enriched

(S11A Fig.). Hierarchical clustering of the species, based on the GO-distribution profiles (i.e. by enumerating the number of genes assigned to each category of GO) of their entire proteomes, produced the expected tree topology that was congruent with the species phylogenetic relationships (S11B Fig.). To identify GO terms that define a tree topology that is respective of the species virulence profiles, we used a random forest (RF) approach, a statistical method that can be used for an unbiased ranking and filtering from large datasets of biomarkers (e.g. genes) that are associated with a given molecular signature or pattern [15-17]. Using this approach, a total of 24 GO terms were identified that could possibly underlay the clustering of *P. eumusae* together with *P. fijiensis*, when considering changes in the predicted proteomes of the three species (S11C Fig.). Consequent mapping of these GO terms on a directed acyclic graph (DAG) that illustrates the connections among the different terms in the form of parent-to-child relationships, indicated that the majority of identified GO terms (16/24) are associated with metabolic processes (GO: 0008152) (S12 Fig.). Included as child nodes are five terms that are directly related to regulation of metabolic processes (GO: 0019222) such as regulation of primary metabolic processes (GO: 0080090), regulation of nitrogen compounds (GO: 0051171) and others, and six terms that are associated with cellular metabolic processes (GO: 0044237) such as pyridine (GO: 0019507) and receptor (GO: 0043112) metabolic processes. Interestingly, among the identified GO terms that contribute to the clustering of *P. eumusae* together with *P. fijiensis* is the one referring to pigmentation (GO: 0043473) (S12 Fig.), a feature that is known to play a role in virulence of fungi (Liu and Nizet 2009). Overall, the GO analysis corroborated results from the KOG-based analysis that changes related to metabolism, such as those related to the regulation of metabolic processes, have played an important role in the evolution of virulence in the sigatoka disease complex.

CAZy annotations and characterization of plant cell wall degrading enzymes (PCWDEs) suggest small differences among the three species but also more similar profiles for *P. eumusae* and *P. fijiensis* as compared to *P. musae*.

Cellulose, hemicelluloses, and pectins are the main constituents of plant cell wall polysaccharides, but their amount can vary significantly in different plant species. To enable their efficient degradation, fungi produce an array of carbohydrate-active enzymes (CAZymes) that consequently play a major role in defining their aggressiveness as plant pathogens and their ability to acquire different sources of organic

matter for their nutrition [18]. Traditionally, CAZymes have been organized into five major superfamilies, comprised of glycoside hydrolases (GHs), glycosyltransferases (GTs), polysaccharide lyases (PLs), carbohydrate esterases (CEs), and carbohydrate-binding modules (CBMs) [19]. Of these, CBMs do not possess any catalytic properties but rather promote the interaction of the enzyme with the target polysaccharide substrate, thus increasing the efficiency of catalysis. A sixth superfamily of alternative enzymatic partners with auxiliary activities (AAs) has been recently added to the original five, which incorporates lytic polysaccharide monooxygenases and redox enzymes that enhance the activity of the original GH, PL and CE enzymes by promoting the oxidation of cell wall components [20].

To assess their ability to degrade and metabolize different polysaccharides, we annotated and compared the repertoire of putative CAZymes present in *P. musae, P. eumusae* and *P. fijiensis*, with an emphasis on the characterization of enzymes involved in the breakdown of plant cell walls (PCWs). To identify any features specific to the three banana pathogens, we also contrasted their CAZyme profiles to those of 16 other Dothideomycetous fungi with different nutritional lifestyles and host specificities [2, 3]. Our CAZy annotations revealed a total of 490, 501, and 516 CAZyme modules from all six major superfamilies in the predicted proteomes of *P. musae*, *P. eumusae* and *P. fijiensis*, respectively (S13 Fig.). Of these, GHs are the most abundant, exhibiting also the greatest variability in numbers and diversity at the individual family level (S4 Table). More specifically, a total of 222, 236 and 244 putative GHs from 61, 60 and 59 different GH families were recovered from the genomes of *P. musae, P. eumusae,* and *P. fijiensis*, respectively, representing 45.3%, 47.1%, and 47.2% of their total CAZyomes. In contrast, GTs, CEs, PLPs, CBMs and AAs for the three species accounted on average for approximately 21.3%, 12.4%, 1.4%, 14.8% and 12.4% of their CAZyomes, respectively.

The high number of GHs encoded in genome of the three species and the relative proportions of the rest of the CAZy superfamilies are in agreement to the numbers reported previously in other Dothideomycetous fungi (S13 Fig.) [2, 3]. A Mann-Whitney U test indicated that the medians of the sums of GHs present in the *P. eumusae, P. musae* and *P. fijiensis* lineage*,* on one hand, and the group of 16 Dothideomycetes, on the other, are not significantly different to each other (P = 0.433). In a similar manner, the Mann-Whitney U test also failed to detect any significant differences between these two groups, when considering the rest of the CAZy superfamilies (GTs: P = 0.081, CEs: P = 0.217, AAs: P = 0.157, CBMs: P = 0.157, PLPs:

P = 0.217). However, at the individual family level many differences can be present between the two groups (S4 Table; S14 Fig.). Family GH25, for example, which includes enzymes with lysozyme activities that cleave the bacterial cell-wall polymer peptidoglycan and are produced by many organisms as a defensive mechanism against bacteria [21], is only present in the three banana pathogens and the pine tree pathogen *D. septosporum* but is absent in any of the other Dothideomycetes. Overall, the Mann-Whitney U test indicated that 15 GH, 3 CE, 8 GT, 2 AA, and 5 CBM families are over- or under-represented in the genomes of the three banana pathogens as compared to the other 16 species of Dothideomycetes (S4 Table). Such differences in the specific enzymatic repertoire of different fungi are not uncommon and are likely a reflection of their diverse nutritional strategies and adaptation to different hosts and the host environment [18].

To further investigate whether there is an association between the arsenal of CAzymes present in each species and their nutritional lifestyles, we performed hierarchical clustering based on the total number of CAZymes present in each individual family. The clustering pattern of the species broadly followed their taxonomic division into Capnodiales, Hysteriales and Pleosporales rather than their nutritional lifestyle, although within each individual order many deviations from the expected species phylogeny could be observed (Fig. 7). Of marked importance, *P. eumusae* clustered together once more with *P. fijiensis* rather than *P. musae,* as expected based on the phylogenetic placement of the three species, indicating that *P. eumusae* and *P. fijiensis* share additional complementary patterns of expansions and contractions in CAZyme families. Such similarities in changes in family sizes between the two more aggressive on *Musa* host species, could thus reflect adaptive expansions or contractions that would suggest that the CAZyomes of these two species have converged towards a better exploitation of their banana host.

To look for functional bias in the pattern of expansions and contractions, as well as compare the relative abundance of CAZymes in the three species, we inspected the putative biological roles that these enzymes might have by assigning each CAZy family to its broader substrate preference and function. A closer examination in the functional diversity of the CAZymes present in *P. musae*, *P. eumusae* and *P. fijiensis*, revealed that cell wall degrading enzymes (CWDEs) constitute the majority of CAZymes encoded in their genomes, representing 44.9% (220/490), 46.9% (235/501) and 47.1% (243/516) of their total CAZyomes, respectively (S15 Fig.). Plant cell wall degrading enzymes (PCWDEs), in particular, are most numerous in

the three species, accounting for about a quarter of their CAZyomes (i.e. *Pm*: 119/490, 24.3%; *Pe:* 125/501, 25.0%; *Pf:* 130/516, 25.2%) (S5 Table). Moreover, within the group of PCWDEs, the ones directed towards the degradation of hemicellulose (*Pm:* 54.6%, *Pe:* 55.2%, *Pf:* 53.1%) are found in higher numbers as compared to enzymes involved in the decomposition or hemicellulose-pectin complexes (*Pm:* 21.0%, *Pe:* 22.4%, *Pf:* 21.5%), pectin (*Pm:* 21.0%, *Pe:* 20.8%, *Pf:* 22.3%), and cellulose (*Pm:* 3.4%, *Pe:* 1.6%, *Pf:* 3.1%) (S5 Table). The higher number of hemicellulases in these species is not unusual among plant pathogenic fungi, which in general have a much higher and more diverse arsenal of lignocellulolytic (i.e. decomposition of cellulose, hemicellulose, and lignin) enzymes as compared to pectinases [3, 18]. It should be noted that from the above calculations we excluded families such as GH1, GH3, GH5, and GH9, whose members can act both on plant and fungal cell walls, and which have been placed in the generic category of cell wall degraders.

Despite the fact that the three banana pathogens share similar numbers in PCWDEs, overall, they display some differences at the individual family level. Such differences can be both in the diversity of families present in each species as well as in the number of members in each family (S6 Table; S16 Fig.). More specifically, from the total number of 34 CAZy families present in at least one of the three species and associated with PCW decomposition, 29 of these families are found in all three species, two (GH11 and GH95) are present in only two species, and three (GH74, GH39 and GH88) are present in just one of the species. Also, 8 of the 35 families differ by two or more members among the three species (S6 Table; S16 Fig.). For example, when considering the GH43 family, one of the most abundant GH families in fungi that includes enzymes for the enzymatic breakdown of hemicellulose-pectin complexes, *P. fijiensis* has 16 members encoded in each genome, while *P. eumusae* and *P. musae* have 19 and 14 members, respectively. Also in this case, hierarchical clustering of the species based on their PCWDE distribution profiles demonstrated that *P. eumusae* and *P. fijiensis* form a single group with strong bootstrap support, thus revealing that they share more similar patterns of average family sizes for PCWDEs as compared to *P. musae* (Fig. 7).

When compared to other Dothideomycetes, the median numbers of hemicellulases and other types of PCWDEs present in the three species are not, based on Mann-Whitney U tests, significantly different from the corresponding median numbers in the group of 16 Dothideomycetes or the subset of five

hemibiotrophic fungi from the Capnodiales clade (S6 Table) [2, 3]. Along the same lines, we could not distinguish any clear differences in the overall arsenal of PCWDEs between monocot and dicot infecting species. At the individual family level, however, the Mann-Whitney U test indicated that family GH74 involved in the degradation of cellulose is again underrepresented (or entirely missing) in the genomes of *P. musae, P. eumusae,* and *P. fijiensis* as compared to the five hemibiotrophic species of Capnodiales. In contrast, families CE1 and CE3 of esterases, whose members display hemicellulolytic activity, family GH51, whose members are involved in the degradation of hemicellulose-pectin complexes, and families GH78 and GH115, which include many enzymes with pectinolytic activity, are overrepresented in the genomes of *P. musae, P. eumusae,* and *P. fijiensis* as compared to the five hemibiotrophic Capnodiales (S6 Table). Such differences at the individual family level could be the result of adaptation of *P. musae, P. eumusae,* and *P. fijiensis* to their banana host, as they are frequently observed among species adapted on different host species. Similar observations at the individual family level could also be made when the three species were compared to the larger group of 16 Dothideomycetes included in this study. Family GH78, for example, which includes enzymes with α-L-rhamnosidase activity involved in the removal of L-rhamnose from PCWs [22], is largely expanded in the genomes of *P. musae, P. eumusae* and *P. fijiensis* as compared to the other 16 Dothideomycetes. The most prominent observation in comparisons with the group of the 16 Dothideomycetes is that cellulolytic enzymes, in general, are clearly underrepresented in the genomes of the three banana pathogens, as they are in the genomes of the other five Capnodiales that were included in these comparisons (S6 Table).

Annotation of the core enzymes involved in the biosynthesis of secondary metabolites (SMs) reveals that the three Sigatoka species potentially produce a diverse but only partially overlapping array of SMs.

Filamentous fungi secrete numerous secondary metabolites (SMs) to alter and adapt to their environment [23]. These low-molecular-weight metabolic products display a wide range of chemical structures that translate into diverse biological functions and important ecological roles, including parasitic infection of the host, antagonistic interactions with other microorganisms, and several others [23, 24]. For plant pathogenic fungi, SMs are commonly produced during infection of the host and are important determinants of fungal pathogenicity, also frequently defining the host range of the producing pathogen.

Host-specific toxins (HSTs), in particular, which are almost exclusively produced by plant-pathogenic species of Dothideomycetes, are most frequently the products of secondary metabolism and play a fundamental role in host-specialization of these pathogenic fungi [25]. In addition, many phytopathogenic Dothideomycetes also produce an array on highly toxic SMs that function as non-HSTs against a wide variety of plant species [23-25].

Inventory of the genes encoding for the four core enzyme types that catalyze the first committed step in the biosynthesis of the major SM classes found in fungi (i.e. polyketide synthases:PKSs, non-ribosomal peptide synthases: NRPSs, terpene synthases:TSs, and dimethylallyl tryptophan synthases:DMATSs) [23], identified a total of 28, 27, and 21 genes in the genomes of *P. musae*, *P. eumusae*, and *P. fijiensis,* respectively. The majority of core enzymes in all three species are predicted as PKSs (7 in *Pm*: PksA-to-PksG, 10 in *Pe*: Pks1-to-Pks10, and 7 in *Pf*: PksI-to-PksVII), followed by NRPSs (10 in *Pm*: NpsA-to-NpsK, 7 in *Pe*: Nps1-to-Nps6, and 8 in *Pf*: NpsI-to-NpsVII) or hybrid PKS-NRPSs (1 in *Pm*: PksNpsA, 2 in *Pe:* PksNps1 and PksNps2, and 2 in *Pf*: PksNpsI and PksNpsII), and finally TSs (5 in *Pm*: TsA-to-TsG, 5 in *Pe:* Ts1-to-Ts5, and 4 in *Pf*: TsI-to-TsIV) (S7 Table). No DMATs were detected in any of the three species. The number and type of core SM genes predicted in the genomes of the three banana pathogens are comparable to those reported previously for other species of Capnodiales, including the close-related tomato pathogen *F. fulva*, the wheat pathogen *Z. tritici,* and the poplar pathogen *S. populicola* [2, 3]. Unfortunately, a complete and reliable annotation of the full biosynthetic gene clusters in which the identified core SM genes are embedded was not possible, mainly due to the highly fragmented genome assemblies, especially for *P. eumusae* and *P. musae*.

Analysis by AntiSMASH and manual curations of the domain architectures of the core enzymes indicated that nearly all of the PKSs present in the predicted proteome of the three species belong to the subcategory of interative type I PKSs, with a higher number predicted in *P. eumusae* (*n*=10) than in *P. musae* (*n*=7) and in *P. fijiensis* (*n*=6) (S7 Table). As expected, no type II PKSs were found in any of the three species, while one type III was identified in *P. eumusae*, in agreement with the rare presence of these two PKS types in fungi. Moreover, with the exception of Pks5 from *P. eumusae*, all other type I PKSs from the three species contain at least the minimum set of ketosynthase (KS), acyltransferase (AT), and acyl carrier protein (ACP) domains and are thus likely to be functional. Inspection of their predicted domain architectures also

indicated that 4 of the PKSs from *P. musae* (PksB, PksC, PksE, PksF), 5 from *P. eumusae* (Pks2, Pks3, Pks7, Pks8, Pks10), and 3 from *P. fijiensis* (PksII, PksIII, PksVI) can be further classified as non-reducing type (NR), suggesting that they are involved in the biosynthesis of aromatic compounds. The remaining 3 PKSs from *P. musae* (PksA, PksD, PksG), 4 from *P. eumusae* (Pks1, Pks4, Pks6, Pks9), and 3 from *P. fijiensis* (PksI, PksIV, PksV) can accordingly be classified as highly (HR) or partially-reducing (PR) PKSs (S7 Table), alluding to the production of aliphatic compounds or reduced polyketide chains, respectively.

To better understand the type of SMs that might be produced by the three species, we performed a phylogenetic analysis with other fungal core PKS enzymes that are involved in the biosynthesis of well characterized SMs, such as aflatoxins, fumonisins, and others (S17 Fig.). A comprehensive list and annotation of these SMs is included in the recent publications by Collemare et al (2014) [26] and Gallo et al (2013) [27]. Our phylogenetic analysis also included PKS-NRPSs, as these enzymes produce related hybrid polyketide structures. To avoid biasing the results, only the highly conserved KS and AT domains were used for tree construction. The analysis showed that most of the NR-PKSs from the three banana pathogens could be clustered with high support (ML bootstrap values ≥80%) with enzymes that are involved in the biosynthesis of known phyto- and mycotoxins in other fungi, and could be involved in the production of structural analogs with matching backbones. More specifically, PksE from *P. musae* as well as Pks6 and Pks7 from *P. eumusae* were found clustered with the Pks13 from *Fusarium graminearum* (ML bootstrap of 84%), which together with the reducing Pks4 from the same species are involved in the production of zearalenone, a notorious mycotoxin produced by species of *Fusarium* spp. with estrogenic activity in animals [28]. However, despite the fact that the *P. eumusae* Pks6 is annotated as a reducing type, both *P. eumusae* and *P. musae* seem to lack orthologues of Pks4 from *F. graminearum*, and thus, they are unlikely to produce zearalenone [29]. Moreover, the orthologous PksC, Pks3, and PksIII enzymes from *P. musae, P. eumusae,* and *P. fijiensis*, respectively were clustered (bootstrap value of 99%) with core enzymes that mediate the biosynthesis of the anthraquinone endocrocin in species of *Aspergillus* spp. Anthraquinones are well-known for their array of industrial and medical uses but also as precursors to the synthesis of aflatoxin intermediates [30]. Clustered with the orthologous PksB, Pks2, and PksII (bootsrap of 100%) from *P. musae, P. eumusae,* and *P. fijiensis*, respectively, was EfPks1, which is involved in the biosynthesis of elsinochromes in the citrus pathogen *Elsinoë fawcettii*, a group of light-activated, non-host specific toxins with a role in pathogenesis for this Dothideomycete species [31]. Along the same lines,

Pks10 from *P. eumusae* was orthologous to Ctb1 from *Cercospora nicotinanae,* which is required for the biosynthesis of the light-activated, non-HST cercosporin in this fungal species [32]. Although cercosporin production has, to the best of our knowledge, not yet been reported outside the genus of *Cercospora*, the production of photoactivated phytotoxins by the three pathogens that constitute the Sigatoka disease complex has been frequently observed under various conditions [33-38]. Thus, our analysis of core SM genes corroborates these earlier experimental findings, suggesting the possible involvement of light-activated phytotoxins in the pathogenesis of the three species. Finally, PksF and Pks8 from *P. musae* and *P. eumusae*, respectively, are likely orthologous to core enzymes involved in the biosynthesis of azaphilones, a structurally diverse class of fungal metabolites that exhibit a wide range of biological activities, including antimicrobial, antifungal and antioxidant activities [39]. Similarly to NR-PKSs, analysis of orthologous relationships among HR-PKSs and PR-PKSs from the three banana pathogens and other fungi showed strong clustering (bootstrap value of 100%) of the Pks4 and PksIV enzymes from *P. eumusae* and *P. fijiensis*, respectively, with Fum1 from *Fusarium oxysporum*, the key enzyme involved in the biosynthesis of fumonisin. Fumonisins are non-HSTs with a role in virulence of *Fusarium* spp. and are particularly notorious for the diverse mycotoxicoses that they can cause in animals and humans [40]. Strong clustering (bootstrap value of 100%) was also seen between Pks9 from *P. eumusae* and PksN from *Altermatia solani*, an enzyme involved in the biosynthesis of the decaketide compound alternapyrone [41], while the *P. musae* PksD and PksV enzymes were grouped with the *Alternatia alternata* DEP5, which is involved in the biosynthesis of depudecin, an inhibitor of histone deacetylase (HDAC) with a minor role in virulence of *A. brassicicola* on cabbage [42]. Finally, none of the hybrid PKS-NRPS enzymes from the three species were strongly clustered with any of the known fungal core enzymes included in this study. Taken together, the phylogenetic analysis indicates that although the three pathogens share some orthologous core PKS enzymes, they still exhibit considerable variation in the arsenal of SMs that they potentially produce, some of which may bare structurally similarity, at least in their backbone structure, to already characterized phyto- and mycotoxins.

Similar overall results were extracted by an analysis of the NRPSs present in the three banana pathogens (S18 Fig.). Like PKSs, NRPSs are megasynthases consisting of several enzymatic modules that elongate the backbone amino acid chain according to the collinearity rule. The minimal set of core domains required for a functional NRPSs module are an amino acid adenylation domain (A), a thiolation (T) or peptidyl carrier

protein (PCP) domain, and a condensation domain (C), while additional optional domains maybe present as well. Domain annotations indicated that the majority of NRPSs predicted in the genome of the three banana pathogens are putatively functional, consisting of one or several repeats of the three elemental domains present in NRPSs. Exception are two NRPSs in *P. musae* (NpsH and NpsK), one in *P. eumusae* (Nps6), and one in *P. fijiensis* (NpsVII), which lack at least one of the three core domains and which thus may be non-functional. Phylogenetic analysis based on the A domains of the fungal NRPS and NRPS-like enzymes included in the recent study by Collemare et al. (2014) [26] and the A domains of the NRPS enzymes encoded in the genomes of the three banana pathogens, indicated mostly weak clustering among the various NRPSs and the presence of only a handful of clearly identifiable orthologs. More specifically, the analysis showed that NPRSs from the three banana pathogens are mainly present within three of the nine subfamilies in which fungal NRPSs can be classified [43], i.e. the subclasses of siderophore synthases (SID), cyclosporine synthases (CYCLO) and Euascomycete-only synthases (EAS). Within the SID subgroup, the three A domains of the multimodular NpsA, Nps1, and NpsI enzymes from *P. musae*, *P. eumusae,* and *P. fijiensis*, respectively, were seen clustered with high to median support (ML bootstrap values of 71%, 83% and 98%) with the corresponding A domains of the SSM1 and Nps2 siderophore synthetases from *Magnaporthe oryzae* and *Cochiobolus heterostrophus*, respectively, which catalyze the first step in the biosynthesis of the intracellular storage siderophore ferricrocin. This hexa-peptide was shown to be essential for several biological processes, including conidiation (*Aspergillus nidulans* and *Neurospora crassa*) and germination of conidiospores (*A. nidulans*), sexual development (*A. nidulans*), oxidative stress resistance (*A. nidulans*), and virulence on host plants (*Pa. oryzae*) [44-46]. Within the CYCLO subgroup, NpsV from *P. fijiensis* is a monomodular enzyme with no homologs in the other two species, while the orthologous NpsB, Nps2, and NpsII from *P. musae, P. eumusae,* and *P. fijiensis*, respectively, are hybrid multimodular enzymes with two A domains, one of which is clustered within the CYCLO clade and the other within the EAS clade. Within the EAS subgroup, week clustering (bootsrap of 69%) was seen between NpsG from *P. musae* with SidD and Nps6 from *Aspergilus fumigatus* and *Bipolaris maydis*. Homologues of SidD and Nps6 are broadly conserved among siderophore-producing ascomycetes and are virulence determinants in many plant pathogenic fungi, most likely being involved in the biosythensis of coprogen-type siderophores [47]. Also, a blast search of the published NRPSs (NPS1-12) in *B. maydis* [46] [48] against the three genomes, next to the homologs mentioned above,

yielded a homolog of *B. maydis* Nps10 in all three species (i.e. NpsJ, Nps7 and NpsVIII). Although the function of this encoded product of this gene is unknown [48], it is the most conserved NRPS in all Dothideomycetes genomes examined so far [36]. Finally, a phylogenetic tree of the fungal TSs orthologous to the ones present in the three species that constitute the Sigatoka disease complex is also presented at S19 Fig.

Effector characterization indicates that the three pathogens exhibit overlapping but still very dissimilar repertoires of candidate effectors.

The secretome, or extracellular proteome, constitutes a dynamic part of the proteome, and secretome analysis offers the means to understanding microbial pathogenicity and host-adaptation. Effectors, in particular, are low molecular weight proteins that are secreted by microbes during pathogenesis to suppress or evade the host immune system and thus, aid the proliferation of disease [49, 50]. The use of comparative genomics within a phylogenetic framework has revealed large differences in effector repertoires among plant pathogens specializing on the same or different hosts, which contributes to, and even sometimes defines, the underlying differences in virulence and host specificity. Thus, differences in effector repertoires can be indicative of changes in virulence and evolutionary adaptations on specific hosts, whereas similarities can reveal the pathogenic core utilized by microbes to infect their hosts. We have recently shown, for example, that homologs of the Avr4 and Ecp2 effector proteins from the tomato pathogen *F. fulva* are present in *P. fijiensis* and other Dothideomycetes, and despite the low levels of sequence homology shared among them, their intrinsic function is mostly conserved [2, 51, 52].

To gain a deeper insight into the pathogenic potential of the three species that constitute the Sigatoka disease complex, we characterized their secretomes, placing an emphasis on identifying and comparing their arsenal of candidate effector repertoires. We broadly defined as effectors the subgroup of secreted proteins that were shorter than 250 amino acids in length with a cysteine content that was at least two-fold higher than the average cysteine content of the full proteome in the individual species [3]. We used the arbitrary chosen length of 250 amino acids and not 200 used previously in other comparative genomics studies within Dothideomycetes [2, 3, 46] as some already characterized effectors (e.g Ecp2-3 of *P. fijiensis,* 239 amino acids) are larger than 200 amino acids in length [51, 52]. By reciprocal BlastP best hit (e-value: 1e-5) analysis implemented in OrthoMCL, we also retrieved the set of candidate effector proteins

shared by the three species, while BlastP (e-value: 1e-5, alignment coverage > 50%) against the NCBI nr database and the JGI fungal genome database was used to identify putative homologs in other fungal species and beyond. As for the full proteome, we defined "*core*", as those effectors shared by the three species and other fungal species as well, while we classified "*lineage-specific*" as the subset of core effectors that are present only in the three pathogens that constitute the Sigatoka disease complex. We also considered effectors that are found in only one of the three pathogens but not in the other two as "*species-specific*", while we classified "*orphans*" as the subcategory of species-specific effectors that do not have homologs in any other fungal species.

Secretome and effector identification was performed using the bioinformatics workflow presented in S20 Fig., which consisted of a number of filtering steps that progressively increased the probability of identifying truly secreted proteins and effectors [53]. A total of 612, 638, and 584 secreted proteins were predicted in the genomes *P. musae, P. eumusae*, and *P. fijiensis*, respectively, indicating that the three species employ secretome arsenals of comparable size to the secretomes of most other hemi-biotrophic fungi, but smaller as compared to necrotrophic pathogens (Mann-Whitney U test, *P*-value = 0.01) (S8 Table). Using the criteria listed above, a total of 110, 112, and 105 putative effector proteins could be retrieved from the predicted secretomes of *P. musae, P. eumusae,* and *P. fijiensis*, respectively. Pfam and homology-based functional annotations of the effector arsenal of the three species indicated that included among the core effectors shared by the three banana pathogens and other fungi are three paralogs of Ecp2 (i.e. Ecp2-1, Ecp2-2, and Ecp2-3) [52] and homologs of the *F. fulva* Ecp6 [54] and Avr4 [55] chitin-binding effectors (S9 Table). Notably, a second paralog of Avr4, which we termed Avr4-2, could be found in the genome of the three banana pathogens and other Dothideomycetous fungi as well. Whether Avr4-2 has a function similar to Avr4 is currently unknown. Almost all other core effectors have hits to hypothetical proteins in other fungi and do not contain any functional domains based on Pfam annotations. Also none of the lineage-specific effector families matched to Pfam domains, while Pfam-based functional annotations of the orphan effectors in each species indicated that except for one effector from *P. musae* that has a Pfam hit to a Rapid ALkalinization Factor (RALF) domain (PF05498), all others could not be assigned a specific function and thus represent novel effectors (S9 Table). Notably, RALFs are a family of ubiquitous plant-derived secreted peptides that induce rapid apoplast alkalization upon pathogen infection and regulate other important aspects of plant growth and development [56]. Although RALFs are

presumably restricted only to plants [57], a functional RALF-like protein has been recently reported in the plant pathogenic fungus *Fusarium oxysporum* in which it promotes pathogenicity on tomato plants and has assumingly been acquired from plants through horizontal gene transfer [58]. Thus, the presence of a RALF-like secreted protein in *P. musaea* could represent a similar case of a horizontally derived effector from plants with a yet unknown role in virulence of the fungus. Of the species-specific effectors present in each species, four could be functionally annotated in *P. eumusae*, including a homologue of the Ecp1 effector protein from *F. fulva* [50] and of the MgSM1 cerato-platanin protein family effector from *Pa. oryzae* [59] (S9 Table). Both these effectors are shown to be virulence factors in their respective species and thus their homologs in *P. eumusae* might have a similar role in virulence as well. The third effector from *P. eumusae* had a hit to MD-2-related lipid-recognition (ML) domain (PF02221, which is implicated in lipid recognition. Similarly, six of the species-specific effectors *P. musae* could receive Pfam-based functional annotations with hits to a Rare lipoprotein A (RlpA) domain (PF03330), a Lipocalin-like domain (PF08212, a multicopper oxidase (PF07731), a fungal hydrophobin (PF06766), and finally a putative Ecp2-like necrosis-inducing factor domain (PF14856) (S9 Table). This could potentially represent a 4th paralog of the Ecp2 effector family present in *P. musae,* although its sequence is highly diverse as compared to the other three Ecp2 paralogs present in this species. Finally, search for Pfam domains in the species-specific effectors of *P. fijiensis* identified one effector with a hit to the Ser-Thr-rich glycosyl-phosphatidyl-inositol-anchored membrane family (PF10342), two effectors with a hit to a cutinase (PF01083) and a peptidase (PF13933) enzyme, respectively and two more effectors containing putative RALF domains (PF05498) (S9 Table). Neither of these two effectors, however, shared significant homology over the entre protein to the RALF-like effector identified in *P. musae*.

The clustering analysis suggests that the three pathogens, despite their very close evolutionary relationships, common infection biology and host range, exhibit a considerably diverse arsenal of effector proteins that could have contributed to their differences in virulence. However, caution needs to be taken regarding the numbers listed above, as when the effector repertoire of each species was used as query in Blastp searches (e-value: 1e-5, alignment coverage > 50%) against the entire proteome of the other two species, then additional putative homologs could be identified that were not annotated as effectors, either because they were larger than 250 amino acids in length or because they were not predicted as secreted proteins in the other species (S9 Table; S21B-D Fig.). For example, BlastP-based query of the *P. eumusae*

effector repertoire against the predicted effectorome and proteome of *P. musae*, returned 39 and 75 protein hits, respectively, indicating that the true number of effectors shared by the two species could be considerably higher. From the additional 36 proteins that were retrieved as blast hits against the entire proteome of *P. musae*, 7 were larger than 250 amino acids, while the remaining 29 were missing a signal peptide. Manual curation of a randomly selected set of six of these non-secreted proteins indicated that in two cases they represented misannotations and an alternative ORF could be found that corresponded to a putatively homologous secreted protein. When considering all the blastp hits of a species effectorome against the entire predicted proteome of the other two species, then the number of species-specific and orphan effectors is reduced to 33 and 20 in *P. musae*, 27 and 17 for *P. eumusae,* and 43 and 36 in *P. fijiensis,* respectively (S9 Table; S21B-D Fig.). This, however, still remains a relatively high number of species-specific and orphan effectors in each species, while *P. eumusae* and *P. musae* continue to share a larger number of putative homologous effectors as compared to putative effectors shared between *P. fijiensis* and *P. eumusae* or between *P. fijiensis* and *P. musae*.

Supplementary References

1. Hane JK, Rouxel T, Howlett BJ, Kema GHJ, Goodwin SB, Oliver RP. A novel mode of chromosomal evolution peculiar to filamentous Ascomycete fungi. Genome Biology. 2011;12(5). doi: Artn R45 Doi 10.1186/Gb-2011-12-5-R45. PubMed PMID: ISI:000295732700009.
2. de Wit PJGM, van der Burgt A, Okmen B, Stergiopoulos I, Abd-Elsalam KA, Aerts AL, et al. The genomes of the fungal plant pathogens *Cladosporium fulvum* and *Dothistroma septosporum* reveal adaptation to different hosts and lifestyles but also signatures of common ancestry. Plos Genetics. 2012;8(11). doi: ARTN e1003088 DOI 10.1371/journal.pgen.1003088. PubMed PMID: ISI:000311891600067.
3. Ohm RA, Feau N, Henrissat B, Schoch CL, Horwitz BA, Barry KW, et al. Diverse lifestyles and strategies of plant pathogenesis encoded in the genomes of eighteen Dothideomycetes fungi. Plos Pathogens. 2012;8(12). doi: ARTN e1003037 DOI 10.1371/journal.ppat.1003037. PubMed PMID: ISI:000312907100009.
4. Rouxel T, Grandaubert J, Hane JK, Hoede C, van de Wouw AP, Couloux A, et al. Effector diversification within compartments of the *Leptosphaeria maculans* genome affected by Repeat-Induced Point mutations. Nature Communications. 2011;2. doi: Doi 10.1038/Ncomms1189. PubMed PMID: ISI:000288225900031.
5. Selker EU, Cambareri EB, Jensen BC, Haack KR. Rearrangement of duplicated DNA in specialized cells of Neurospora. Cell. 1987;51(5):741-52.
6. Clutterbuck AJ. Genomic evidence of repeat-induced point mutation (RIP) in filamentous ascomycetes. Fungal Genetics and Biology. 2011;48(3):306-26.
7. Cambareri EB, Jensen BC, Schabtach E, Selker EU. Repeat-induced GC to AT mutations in Neurospora. Science. 1989;244(4912):1571-5.
8. Cambareri E, Singer M, Selker E. Recurrence of repeat-induced point mutation (RIP) in *Neurospora crassa.* Genetics. 1991;127(4):699-710.
9. Hane JK, Oliver RP. RIPCAL: a tool for alignment-based analysis of repeat-induced point mutations in fungal genomic sequences. BMC Bioinformatics. 2008;9(1):478.

10. Goodwin SB, Ben M'Barek S, Dhillon B, Wittenberg AHJ, Crane CF, Hane JK, et al. Finished genome of the fungal wheat pathogen *Mycosphaerella graminicola* reveals dispensome structure, chromosome plasticity, and stealth pathogenesis. Plos Genetics. 2011;7(6). doi: ARTN e1002070 DOI 10.1371/journal.pgen.1002070. PubMed PMID: ISI:000292386300008.

11. Fudal I, Ross S, Brun H, Besnard A-L, Ermel M, Kuhn M-L, et al. Repeat-induced point mutation (RIP) as an alternative mechanism of evolution toward virulence in *Leptosphaeria maculans.* Molecular Plant-Microbe Interactions. 2009;22(8):932-41.

12. Tatusov RL, Fedorova ND, Jackson JD, Jacobs AR, Kiryutin B, Koonin EV, et al. The COG database: an updated version includes eukaryotes. BMC Bioinformatics. 2003;4. doi: Artn 41 Doi 10.1186/1471-2105-4-41. PubMed PMID: ISI:000186341900001.

13. Arzanlou M, Crous PW, Zwiers L-H. Evolutionary dynamics of mating-type loci of *Mycosphaerella* spp. occurring on banana. Eukaryotic Cell. 2010;9(1):164-72.

14. Kaessmann H. Origins, evolution, and phenotypic impact of new genes. Genome Research. 2010;20(10):1313-26. Epub 2010/07/24. doi: 10.1101/gr.101386.109. PubMed PMID: 20651121; PubMed Central PMCID: PMC2945180.

15. Tamura K, Stecher G, Peterson D, Filipski A, Kumar S. MEGA6: Molecular evolutionary genetics analysis version 6.0. Molecular Biology and Evolution 2013;30(12):2725-9. doi: 10.1093/molbev/mst197. PubMed PMID: 24132122; PubMed Central PMCID: PMCPMC3840312.

16. Chen X, Ishwaran H. Random forests for genomic data analysis. Genomics. 2012;99(6):323-9. doi: 10.1016/j.ygeno.2012.04.003. PubMed PMID: 22546560; PubMed Central PMCID: PMCPMC3387489.

17. Touw WG, Bayjanov JR, Overmars L, Backus L, Boekhorst J, Wels M, et al. Data mining in the Life Sciences with Random Forest: a walk in the park or lost in the jungle? Briefings in Bioinformatics. 2013;14(3):315-26. doi: 10.1093/bib/bbs034. PubMed PMID: 22786785; PubMed Central PMCID: PMCPMC3659301.

18. Zhao Z, Liu H, Wang C, Xu J-R. Comparative analysis of fungal genomes reveals different plant cell wall degrading capacity in fungi. BMC Genomics. 2013;14(1):274.

19. Cantarel BL, Coutinho PM, Rancurel C, Bernard T, Lombard V, Henrissat B. The Carbohydrate-Active EnZymes database (CAZy): an expert resource for glycogenomics. Nucleic Acids Research. 2009;37(suppl 1):D233-D8.

20. Levasseur A, Drula E, Lombard V, Coutinho PM, Henrissat B. Expansion of the enzymatic repertoire of the CAZy database to integrate auxiliary redox enzymes. Biotechnology of Biofuels. 2013;6(1):41.

21. Martinez-Fleites C, Korczynska JE, Davies GJ, Cope MJ, Turkenburg JP, Taylor EJ. The crystal structure of a family GH25 lysozyme from *Bacillus anthracis* implies a neighboring-group catalytic mechanism with retention of anomeric configuration. Carbohydrate Research. 2009;344(13):1753-7.

22. Mutter M, Beldman G, Schols HA, Voragen AGJ. Rhamnogalacturonan [alpha]-L-Rhamnopyranohydrolase (A Novel Enzyme Specific for the Terminal Nonreducing Rhamnosyl Unit in Rhamnogalacturonan Regions of Pectin). Plant Physiology. 1994;106(1):241-50.

23. Keller NP, Turner G, Bennett JW. Fungal secondary metabolism - From biochemistry to genomics. Nature Reviews Microbiology. 2005;3(12):937-47. doi: Doi 10.1038/Nrmicro1286. PubMed PMID: ISI:000233668900013.

24. Fox EM, Howlett BJ. Secondary metabolism: regulation and role in fungal biology. Current Opinion in Microbiology. 2008;11(6):481-7. doi: DOI 10.1016/j.mib.2008.10.007. PubMed PMID: ISI:000261866200002.

25. Stergiopoulos I, Collemare J, Mehrabi R, De Wit PJGM. Phytotoxic secondary metabolites and peptides produced by plant pathogenic Dothideomycete fungi. FEMS Microbiology Reviews. 2013;37(1):67-93. doi: DOI 10.1111/j.1574-6976.2012.00349.x. PubMed PMID: ISI:000312302100005.

26. Collemare J, Griffiths S, Iida Y, Jashni MK, Battaglia E, Cox RJ, et al. Secondary metabolism and biotrophic lifestyle in the tomato pathogen *Cladosporium fulvum*. Plos One. 2014;9(1). doi: ARTN e85877 DOI 10.1371/journal.pone.0085877. PubMed PMID: ISI:000330237000070.

27. Gallo A, Ferrara M, Perrone G. Phylogenetic study of polyketide synthases and nonribosomal peptide synthetases involved in the biosynthesis of mycotoxins. Toxins. 2013;5(4):717-42. doi: DOI 10.3390/toxins5040717. PubMed PMID: ISI:000318037500008.

28. Lysoe E, Klemsdal SS, Bone KR, Frandsen RJN, Johansen T, Thrane U, et al. The *PKS4* gene of *Fusarium graminearum* is essential for zearalenone production. Applied and Environmental Microbiology. 2006;72(6):3924-32. doi: Doi 10.1128/Aem.00963-05. PubMed PMID: ISI:000238620100015.

29. Saruwatari T, Praseuth AP, Sato M, Torikai K, Noguchi H, Watanabe K. A comprehensive overview on genomically directed assembly of aromatic polyketides and macrolide lactones using fungal megasynthases. Journal of Antibiotics. 2011;64(1):9-17. doi: Doi 10.1038/Ja.2010.130. PubMed PMID: ISI:000287072300003.

30. Lim FY, Hou YP, Chen YM, Oh JH, Lee I, Bugni TS, et al. Genome-based cluster deletion reveals an endocrocin biosynthetic pathway in *Aspergillus fumigatus*. Applied and Environmental Microbiology. 2012;78(12):4117-25. doi: Doi 10.1128/Aem.07710-11. PubMed PMID: ISI:000304788500007.

31. Liao HL, Chung KR. Genetic dissection defines the roles of elsinochrome phytotoxin for fungal pathogenesis and conidiation of the citrus pathogen *Elsinoe fawcetti*i. Molecular Plant-Microbe Interactions. 2008;21(4):469-79. doi: Doi 10.1094/Mpmi-21-4-0469. PubMed PMID: ISI:000254055600010.

32. Choquer M, Dekkers KL, Chen HQ, Cao LH, Ueng PP, Daub ME, et al. The *CTB1* gene encoding a fungal polyketide synthase is required for cercosporin biosynthesis and fungal virulence of *Cercospora nicotianae*. Molecular Plant Microbe Intercations. 2005;18(5):468-76. doi: Doi 10.1094/Mpmi-18-0468. PubMed PMID: ISI:000228575500010.

33. Cruz-Cruz CA, Garcia-Sosa K, Escalante-Erosa F, Pena-Rodriguez LM. Physiological effects of the hydrophilic phytotoxins produced by *Mycosphaerella fijiensis*, the causal agent of black sigatoka in banana plants. Journal of General Plant Pathology. 2011;77(2):93-100. doi: DOI 10.1007/s10327-010-0288-4. PubMed PMID: ISI:000287925400004.

34. Harelimana G, Lepoivre P, Jijakli H, Mourichon X. Use of *Mycosphaerella fijiensis* toxins for the selection of banana cultivars resistant to black leaf streak. Euphytica. 1997;96(1):125-8. doi: Doi 10.1023/A:1002960902950. PubMed PMID: ISI:A1997XU40800014.

35. Upadhyay RK, Strobel GA, Coval SJ, Clardy J. Fijiensin, the 1st Phytotoxin from *Mycosphaerella fijiensis*, the causative agent of black sigatoka disease. Experientia. 1990;46(9):982-4. doi: Doi 10.1007/Bf01939396. PubMed PMID: ISI:A1990EC20800018.

36. Mohan Jain S, Swennen R. Banana improvement: cellular, molecular biology, and induced mutations: Science Publishers Inc.; 2004.

37. Okole BN. Selection of banana and plantain (*Musa* spp.) tissues resistant to toxins produced by *Mycosphaerella* species using tissue culture techniques1995.

38. Strobel G, Stierle A, Upadhyay R, Hershenhorn J, Molina G. The phytotoxins of *Mycosphaerella fijiensis*, the causative agent of black sigatoka disease, and their potential use in screening for disease resistance. Biotechnology applications for banana and plantain improvement. 1992:93.

39. Osmanova N, Schultze W, Ayoub N. Azaphilones: a class of fungal metabolites with diverse biological activities. Phytochemistry Reviews. 2010;9(2):315-42. doi: DOI 10.1007/s11101-010-9171-3. PubMed PMID: ISI:000280577600009.

40. Desjardins AE, Plattner RD, Nelsen TC, Leslie JF. Genetic-analysis of fumonisin production and virulence of *Gibberella fujikuroi* mating population a (*Fusarium moniliforme*) on Maize (*Zea mays*) seedlings. Applied and Environmental Microbiology. 1995;61(1):79-86. PubMed PMID: ISI:A1995PY86700014.

41. Fujii I, Yoshida N, Shimomaki S, Oikawa H, Ebizuka Y. An iterative type I polyketide synthase PKSN catalyzes synthesis of the decaketide alternapyrone with regio-specific octa-methylation. Chemistry & Biology. 2005;12(12):1301-9. doi: DOI 10.1016/j.chembiol.2005.09.015. PubMed PMID: ISI:000234358400008.

42. Wight WD, Kim KH, Lawrence CB, Walton JD. Biosynthesis and Role in Virulence of the Histone Deacetylase Inhibitor Depudecin from *Alternaria brassicicola*. Molecular Plant-Microbe Interactions. 2009;22(10):1258-67. doi: Doi 10.1094/Mpmi-22-10-1258. PubMed PMID: ISI:000269657400007.

43. Bushley KE, Turgeon BG. Phylogenomics reveals subfamilies of fungal nonribosomal peptide synthetases and their evolutionary relationships. BMC Evolutionary Biology. 2010;10. doi: Artn 26 Doi 10.1186/1471-2148-10-26. PubMed PMID: ISI:000274623900001.

44. Hof C, Eisfeld K, Welzel K, Antelo L, Foster AJ, Anke H. Ferricrocin synthesis in *Magnaporthe grisea* and its role in pathogenicity in rice. Molecular Plant Pathology. 2007;8(2):163-72. doi: Doi 10.1111/J.1364-2007.00380.X. PubMed PMID: ISI:000244279400003.

45. Eisendle M, Schrettl M, Kragl C, Muller D, Illmer P, Haas H. The intracellular siderophore ferricrocin is involved in iron storage, oxidative-stress resistance, germination, and sexual development in Aspergillus nidulans. Eukaryotic Cell. 2006;5(10):1596-603. doi: Doi 10.1128/Ec.00057-06. PubMed PMID: ISI:000241344300003.

46. Condon BJ, Leng YQ, Wu DL, Bushley KE, Ohm RA, Otillar R, et al. Comparative genome structure, secondary metabolite, and effector coding capacity across *Cochliobolus* pathogens. Plos Genetics. 2013;9(1). doi: ARTN e1003233 DOI 10.1371/journal.pgen.1003233. PubMed PMID: ISI:000314651500070.

47. Oide S, Moeder W, Krasnoff S, Gibson D, Haas H, Yoshioka K, et al. NPS6, encoding a nonribosomal peptide synthetase involved in siderophore-mediated iron metabolism, is a conserved virulence determinant of plant pathogenic ascomycetes. Plant Cell. 2006;18(10):2836-53. doi: DOI 10.1105/tpc.106.045633. PubMed PMID: ISI:000241818300031.

48. Lee BN, Kroken S, Chou DYT, Robbertse B, Yoder OC, Turgeon BG. Functional analysis of all nonribosomal peptide synthetases in *Cochliobolus heterostrophus* reveals a factor, NPS6, involved in virulence and resistance to oxidative stress. Eukaryotic Cell. 2005;4(3):545-55. doi: Doi 10.1128/Ec.4.3.545-555.2005. PubMed PMID: ISI:000227781300006.

49. De Wit PJ, Mehrabi R, Van den Burg HA, Stergiopoulos I. Fungal effector proteins: past, present and future. Molecular Plant Pathology. 2009;10(6):735-47. Epub 2009/10/24. doi: 10.1111/j.1364-3703.2009.00591.x. PubMed PMID: 19849781.

50. Stergiopoulos I, de Wit PJ. Fungal effector proteins. Annual Review Phytopathology. 2009;47:233-63. Epub 2009/04/30. doi: 10.1146/annurev.phyto.112408.132637. PubMed PMID: 19400631.

51. Stergiopoulos I, van den Burg HA, Okmen B, Beenen HG, van Liere S, Kema GHJ, et al. Tomato Cf resistance proteins mediate recognition of cognate homologous effectors from fungi pathogenic on dicots and monocots. Proceedings of the National Academy of Sciences of the United States of

America. 2010;107(16):7610-5. doi: DOI 10.1073/pnas.1002910107. PubMed PMID: ISI:000276892300089.

52. Stergiopoulos I, Kourmpetis YAI, Slot JC, Bakker FT, de Wit PJGM, Rokas A. *In silico* characterization and molecular evolutionary analysis of a novel superfamily of fungal effector proteins. Molecular Biology and Evolution. 2012;29(11):3371-84. doi: DOI 10.1093/molbev/mss143. PubMed PMID: ISI:000310167700012.

53. Torto TA, Li S, Styer A, Huitema E, Testa A, Gow NA, et al. EST mining and functional expression assays identify extracellular effector proteins from the plant pathogen Phytophthora. Genome Research. 2003;13(7):1675-85.

54. Bolton MD, Van Esse HP, Vossen JH, De Jonge R, Stergiopoulos I, Stulemeijer IJ, et al. The novel *Cladosporium fulvum* lysin motif effector Ecp6 is a virulence factor with orthologues in other fungal species. Molecular Microbiology. 2008;69(1):119-36.

55. van den Burg HA, Harrison SJ, Joosten MH, Vervoort J, De Wit PJ. *Cladosporium fulvum* Avr4 protects fungal cell walls against hydrolysis by plant chitinases accumulating during infection. Molecular Plant-Microbe Interactions. 2006;19(12):1420-30.

56. Pearce G, Moura DS, Stratmann J, Ryan CA. RALF, a 5-kDa ubiquitous polypeptide in plants, arrests root growth and development. Proceedings of the National Academy of Sciences. 2001;98(22):12843-7.

57. Cao J, Shi F. Evolution of the RALF gene family in plants: gene duplication and selection patterns. Evolutionary Bioinformatics online. 2012;8:271.

58. Masachis S, Turrà D, El Ghalid M, Felix G, Richards AT, A DP, editors. Fusarium Rapid ALkalinization Factor (f-ralf) encodes a secreted virulence effector acquired by horizontal gene transfer from plants. 28th Fungal Genetics Conference; 2015 March 17-22; Asilomar, USA: The Genetics Society of America.

59. Yang Y, Zhang H, Li G, Li W, Wang Xe, Song F. Ectopic expression of MgSM1, a Cerato-platanin family protein from *Magnaporthe grisea*, confers broad-spectrum disease resistance in Arabidopsis. Plant Biotechnology Journal. 2009;7(8):763-77.
